# **Genome-wide characterization of peptidyl-prolyl *cis***–***trans *isomerases in *Penicillium* and their regulation by salt stress in a halotolerant *P. oxalicum***

**DOI:** 10.1038/s41598-021-91602-8

**Published:** 2021-06-10

**Authors:** Mangaljeet Singh, Kirandeep Kaur, Avinash Sharma, Rajvir Kaur, Dimple Joshi, Megha Chatterjee, Iman Dandapath, Amarjeet Kaur, Harpreet Singh, Prabhjeet Singh

**Affiliations:** 1 Department of Biotechnology, Guru Nanak Dev University, Amritsar, Punjab 143005 India; 2 Department of Microbiology, Guru Nanak Dev University, Amritsar, Punjab 143005 India; 3 Department of Bioinformatics, Hans Raj Mahila Maha Vidyalaya, Jalandhar, Punjab 144008 India

**Keywords:** Biochemistry, Biotechnology, Computational biology and bioinformatics, Evolution, Microbiology, Molecular biology

## Abstract

Peptidyl-prolyl *cis–trans* isomerases (PPIases) are the only class of enzymes capable of *cis–trans* isomerization of the prolyl peptide bond. The PPIases, comprising of different families viz., cyclophilins, FK506-binding proteins (FKBPs), parvulins and protein phosphatase 2A phosphatase activators (PTPAs), play essential roles in different cellular processes. Though PPIase gene families have been characterized in different organisms, information regarding these proteins is lacking in *Penicillium* species, which are commercially an important fungi group. In this study, we carried out genome-wide analysis of PPIases in different *Penicillium *spp*.* and investigated their regulation by salt stress in a halotolerant strain of *Penicillium oxalicum.* These analyses revealed that the number of genes encoding cyclophilins, FKBPs, parvulins and PTPAs in *Penicillium* spp*.* varies between 7–11, 2–5, 1–2, and 1–2, respectively. The halotolerant *P. oxalicum* depicted significant enhancement in the mycelial PPIase activity in the presence of 15% NaCl, thus, highlighting the role of these enzymes in salt stress adaptation. The stress-induced increase in PPIase activity at 4 and 10 DAI in *P. oxalicum* was associated with higher expression of *PoxCYP18.* Characterization of PPIases in *Penicillium spp.* will provide an important database for understanding their cellular functions and might facilitate their applications in industrial processes through biotechnological interventions.

## Introduction

The peptide bonds not preceding proline are almost always *trans* in folded proteins, but about 10–15% of all Xaa-Pro (Xaa: other bulky amino groups preceding proline) peptide bonds show *cis* conformation^1,2^. Conversion of Xaa-Pro bond from *cis* to *trans* conformation, imperative for the correct folding of proteins, is a slow rate-limiting step and requires intervention of PPIases^3^. The PPIases are categorized into different classes viz., cyclophilins, FKBPs, parvulins and PTPAs. While cyclophilins bind cyclosporin A (CsA), the FKBPs show affinity for FK506 and rapamycin. The PPIase activity of parvulins is sensitive only to juglone and is not affected by either CsA or FK506^4^. The PPIases that contain both cyclophilin and FKBP domains have also been reported^5^. The PTPAs also exhibit PPIase activity, but are structurally and biochemically distinct from cyclophilins, FKBPs and parvulins and, hence categorized as a different class^6^.

The cyclophilins, defined by a conserved cyclophilin-like domain (CLD), are ubiquitously observed in bacteria to higher organisms^7^, and are encoded by large gene families, with the number ranging from eight in *Saccharomyces cerevisiae* to 19 in humans, 89 in wheat and 91 in *Brassica napus*^8–12^. The FKBPs also constitute a multigene family. Compared to four in *S. cerevisiae*, up to 18 and 29 different family members have been reported in humans and rice, respectively^13–15^. A characteristic feature of FKBPs is the presence of an approximately 110 amino acid (AA) long FK506-binding domain (FKBD) that acts as a receptor of FK506 and rapamycin. The repertoires of parvulins and PTPAs are limited, with only one and three parvulins reported in *Escherichia coli* and humans, respectively^4,16–18^. Likewise, only single PTPA gene was observed in humans, compared to two in S. *cerevisiae*^6,19^.

Besides being implicated in several essential cellular processes, such as receptor complex stabilization, plant growth and development, RNA processing, etc., several cyclophilins have also been implicated in abiotic stress adaptation^7,20–26^. Role in abiotic stress response has also been demonstrated for FKBPs such as wFKBP*77* and *VfFKBP15* in wheat and *Vicia faba,* respectively, and *Sce.FKBP12* in *Scenedesmus* sp.^27–29^. These observations imply that PPIase genes may serve as suitable candidates for enhancing the abiotic stress tolerance of microbes and plants for industrial processes and agricultural applications and, thus, warrant further investigations.

Eco-friendly solutions for industrial production of different biomolecules entail the application of microbial cells. To this purpose, *Penicillium* has long been used for the production of specialized cheese, antibiotics, enzymes and a wide range of other biologically active metabolites^30–32^. However, exposure to NaCl, an integral component of the media used for growth during various fermentation processes, often results in salt stress and adversely affects the growth and metabolism of microbial cells due to Na^2+^ toxicity and osmotic stress. Further, the sensitivity to salt stress also limits the use of seawater as a sustainable substitute for freshwater in industrial processes^33^. Therefore, to develop viable bioprocesses under high salt conditions, it is imperative that the role of PPIases in salt stress tolerance be investigated in the microbial strains.

Halotolerance in fungi is attributed to several different mechanisms viz., maintenance of plasma membrane fluidity and Na^+^ homeostasis, accumulation of compatible osmolytes, and expression of genes implicated in mitochondrial biogenesis and metabolism^34–37^. Synthesis of chaperones such as heat shock proteins and PPIases also provides protection against stress-induced damage to the cell^7,25,26,38,39^. Though PPIases have been characterized in several fungi^40^, and implicated in stress response in *Aspergillus* and *Geobacillus*^41–43^, information about these proteins is lacking in *Penicillium*. Therefore, in the present study, we carried out in silico characterization of cyclophilin, FKBP, parvulin and PTPA gene families in *Penicillium* spp. and analysed the effect of salt stress on intracellular PPIase activity and expression of these genes in a halotolerant strain of *P. oxalicum* that is able to grow in the presence of up to 15% salt (NaCl). These findings revealed that the number of cyclophilins, FKBPs, parvulins, and PTPA in different Penicillium species vary between 7–11, 2–5, 1–2 and 1–2, respectively. The halotolerant isolate of *P. oxalicum* exhibited significant enhancement in the mycelial PPIase activity under salt stress which was also accompanied by a substantial increase in the expression of a cyclophilin gene, *PoxCYP18*. These studies are the first to identify and characterize different PPIase gene families in *Penicillium* and their possible role in salt stress response. The results of these investigations will provide an important database for further elucidation of the role of PPIases in different aspects of growth and development in *Penicillium* which may lead to their potential exploitation for different commercial processes.

## Results

**Figure 1:**
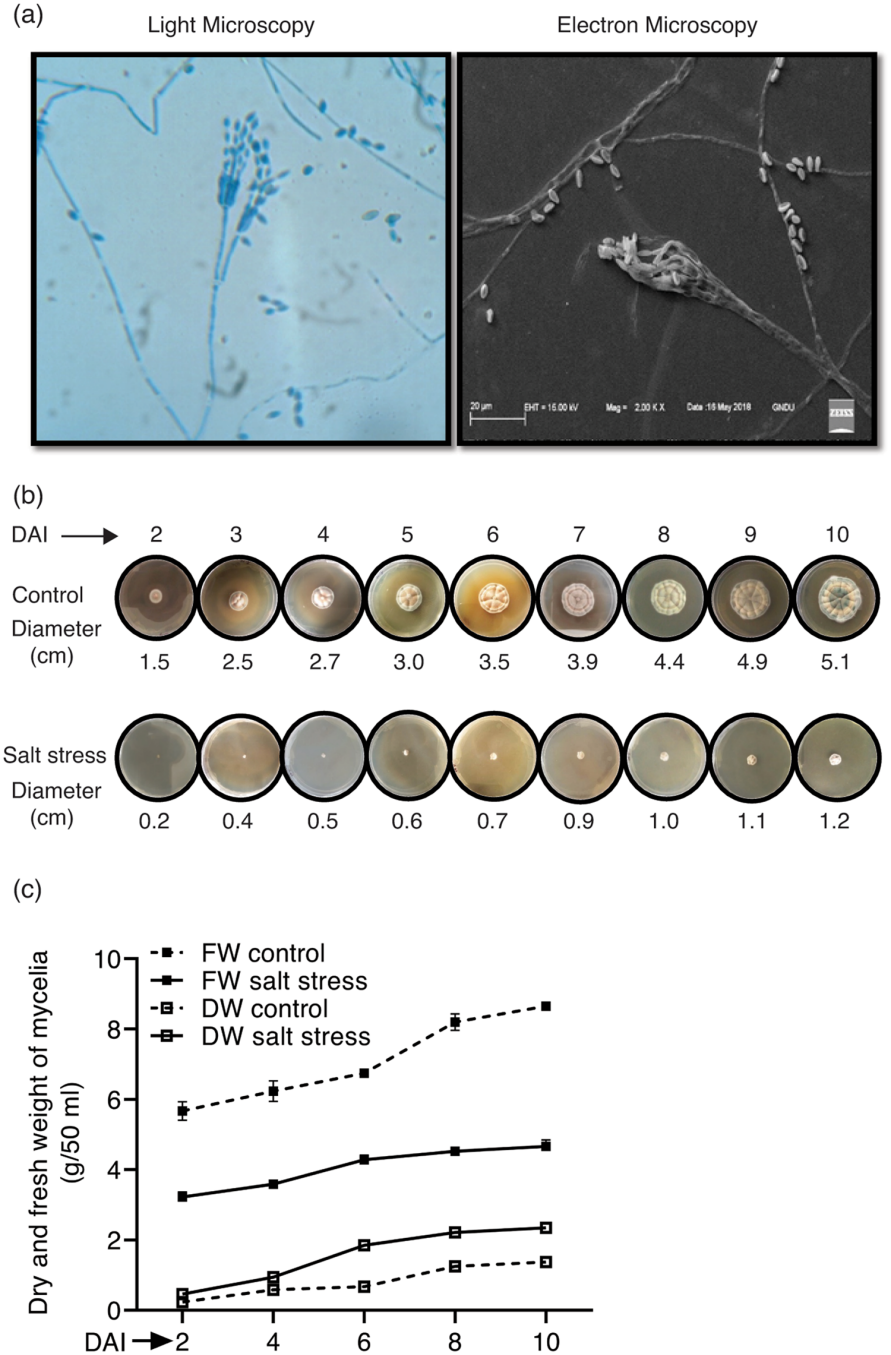
**(a)** Light and scanning electron microscopic observations of *Penicillium oxalicum* conidiophores. (**b)** Growth of *P. oxalicum* on sabouraud agar medium in the presence (lower lane) or absence (upper lane) of salt stress (15% NaCl). **(c)** Effect of salt stress (15% NaCl) on fresh and dry weights of mycelia at different growth stages in *P. oxalicum*. The values depict the mean of three biological replicates ± standard error. (DAI: days after inoculation). The figures were drawn in Adobe Illustrator (v25.2.3) (https://adobe.com/products/illustrator).

The halotolerant endophytic fungal strain used in the present study for expression analysis of PPIase genes was identified as *Penicillium oxalicum*. Microscopic observations revealed fungal hyphae to be highly branched with long brush-like branched conidiophores producing phialides with a short narrow neck. Conidia were smooth-walled, cylindrical to ellipsoidal and produced in chains in long parallel columns (Fig. 1a). Though this strain was able to grow in the presence of up to 15% NaCl, the growth was substantially higher in the medium lacking salt (Fig. 1b) since the colony diameter after ten days of incubation at 30ºC was higher (5.1 cm) compared to salt stress (1.2 cm). Further, relative to the unamended medium, the colonies obtained in the presence of salt were compact (Fig. 1b) and depicted reduced sporulation, since the spore count from equal-sized discs (5 mm) taken from the colonies was lower (8.83 ± 0.76 × 10^5^) in the presence of salt as compared to control (4.51 ± 0.15 × 10^7^). The mycelial fresh and dry weights of the culture were affected differently by salt stress. While the fresh weight was higher in the medium lacking salt, the mycelial dry weight was significantly greater in the presence of NaCl (Fig. 1c), which is in agreement with similar observations reported earlier for other fungi^44^. However, ultrastructure studies and the estimation of compatible solutes are required to understand the mechanism responsible for the salt-induced increase in the mycelial biomass of this strain.

### Bioinformatics analysis

#### Cyclophilins

**Figure 2:**
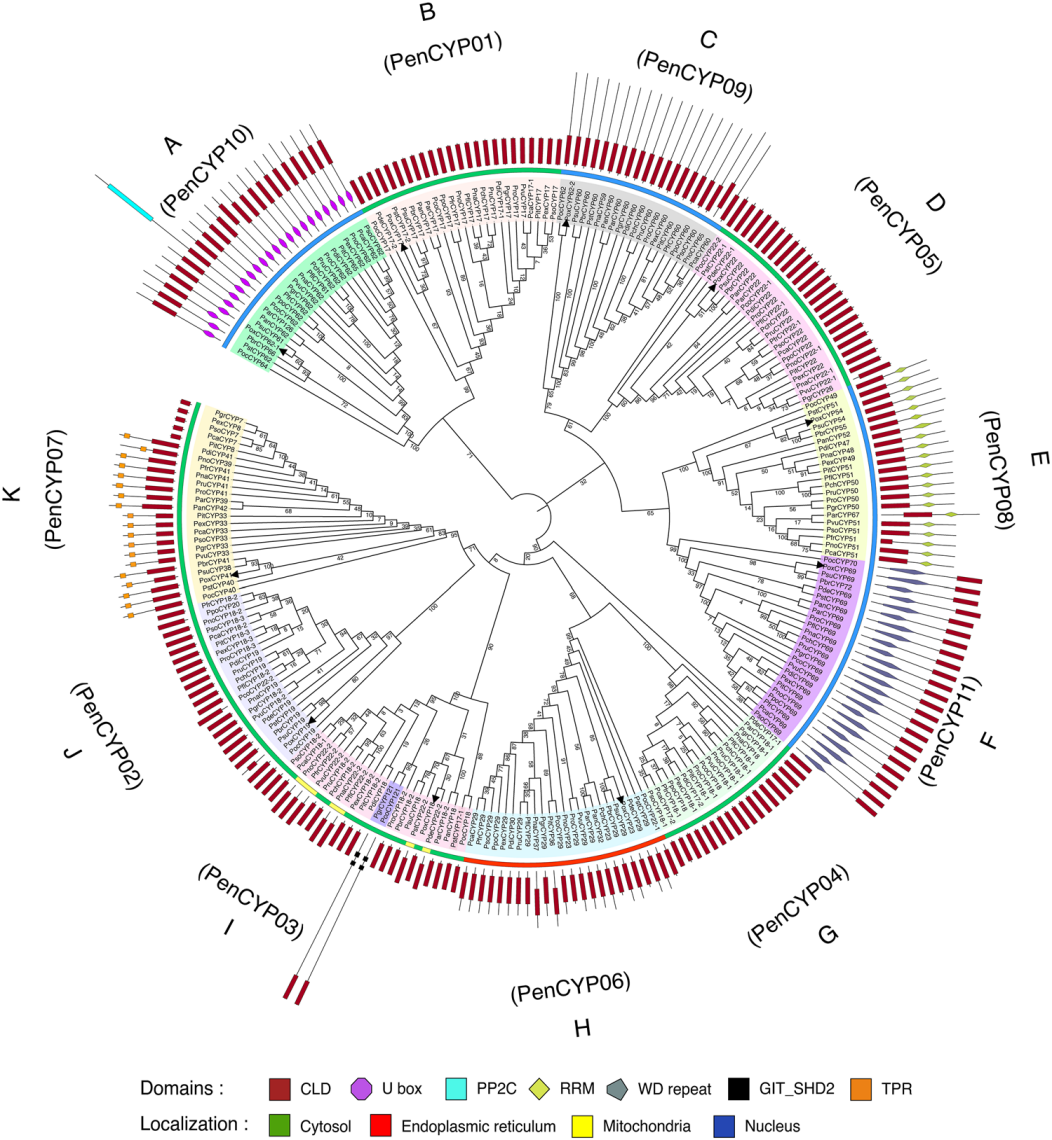
The phylogenetic relationship of different *Penicillium* cyclophilins. The unrooted tree was generated using the neighbor-joining method in MEGA X (v10.1.7) software (http://www.megasoftware.net). The constructed tree was further annotated with the Iterative Tree of Life (http://itol.embl.de/) and redrawn in Adobe Illustrator (v25.2.3) (https://adobe.com/products/illustrator). Bootstrap values from 1000 replicates are indicated at each branch. The localization of each member is represented in different colors. Domain architecture of each protein is also shown in the outermost layer. Black triangles represent the *Penicillium oxalicum*.

In silico analysis revealed 237 full-length CLD-containing putative cyclophilin proteins in different species of *Penicillium* (Table 1). The number of cyclophilins in different *Penicillium* spp. ranges between 7–11, with the *P. oxalicum* genome encoding ten cyclophilins (Supplementary Table S1). Based on homology, the *Penicillium* cyclophilins were clustered into 12 different orthogroups viz., PenCYP01-PenCYP12 (Table 2, Supplementary Table S2a-l), which was also validated by their phylogenetic clustering (Fig. 2). Genes encoding different cyclophilins of the same orthogroup depicted conservation in their intron–exon architecture (Supplementary Fig. S1). While the genes for PenCYP09 cyclophilins (Group C) showed the absence of introns, the genes of orthogroup PenCYP06 (Group H) depicted up to seven introns (Supplementary Fig. S1). The PenCYP01, PenCYP05, PenCYP06 and PenCYP11 members were observed in all *Penicillium* spp., suggesting their essential role (Table 2). The PenCYP12 orthogroup comprises of only two cyclophilins, PcoCYP121 (121.93 kDa) and PgrCYP121 (121.9 kDa), that were observed only in *P. coprophilum* and *P. griseofulvum,* respectively (Supplementary Table S2l). Variable homology was noticed among cyclophilins of different orthogroups, with the interspecific divergence being higher in the members of PenCYP06 and PenCYP10, that showed a minimum similarity of 51.6% and 41.3%, respectively (Supplementary Table S3f, j). On the contrary, the orthogroups PenCYP01, PenCYP11 and PenCYP12 demonstrated greater conservation, with the minimum similarity being 90.7%, 84.2% and 97.1%, respectively (Supplementary Table S3a, k, l).

**Table 1:** Genome-wide analysis of cyclophilin proteins in different *Penicillium* spp*.*

S. no.	*Penicillium *spp.	Genes	Proteins	AA residues	MW (kDa)	pI	SD	MD	Subcellular localization
1	*P. antarcticum*	09	09	162–627	17.76–69.22	5.59–8.26	05	04	C, ER, N
2	*P. arizonense*	10	10	162–1183	17.77–126.82	5.32–8.70	06	04	C, ER, N
3	*P. brasilianumn*	10	10	162–657	17.85–72.46	5.28–8.93	06	04	C, ER, N
4	*P. camemberti*	10	10	73–627	17.73–69.56	5.5–8.450	07	03	C, ER, N
5	*P. chrysogenum*	10	10	162–627	17.70–69.51	5.76–8.59	07	03	C, ER, N
6	*P. coprophilum*	08	08	162–1110	17.77–121.93	5.78–8.61	05	03	C, ER, N
7	*P. decumbens*	07	07	159–629	17.76–69.56	5.93–9.13	06	01	C, ER, M, N
8	*P. digitatum*	11	11	162–627	17.67–69.60	4.68–8.60	07	04	C, ER, N
9	*P. expansum*	10	10	73–627	17.72–69.48	4.56–8.91	07	03	C, ER, N
10	*P. flavigenum*	09	09	162–627	17.71–69.48	5.49–9.14	06	03	C, ER, M, N
11	*P. freii*	11	11	162–627	17.70–69.52	5.42–8.96	07	04	C, ER, M, N
12	*P. griseofulvum*	10	10	73–1108	17.74–121.89	5.68–8.63	06	04	C, ER, N
13	*P. italicum*	10	10	73–627	17.70–69.55	4.66–8.40	07	03	C, ER, N
14	*P. nalgiovense*	11	11	162–627	17.68–69.61	4.63–8.96	07	04	C, ER, M, N
15	*P. nordicum*	11	11	162–627	17.90–69.30	5.42–8.96	07	04	C, ER, M, N
16	*P. occitanis*	10	10	162–631	17.69–70.39	4.72–8.63	06	04	C, ER, N
17	*P. oxalicum*	10	10	162–627	17.79–69.82	5.93–8.87	06	04	C, ER, N
18	*P. polonicum*	08	08	162–627	17.69–69.49	5.54–7.93	06	02	C, ER, N
19	*P. roqueforti*	11	11	162–627	17.74–69.41	5.65–8.89	07	04	C, ER, N
20	*P. rubens*	11	11	162–627	17.70–69.51	5.76–8.59	07	04	C, ER, N
21	*P. solitum*	10	10	73–627	17.72–69.52	5.49–8.45	07	03	C, ER, N
22	*P. steckii*	11	11	161–629	17.63–69.81	5.73–9.32	07	04	C, ER, M, N
23	*P. subrubescens*	10	10	162–629	17.87–69.57	5.48–8.89	06	04	C, ER, N
24	*P. vulpinum*	09	09	162–627	17.76–69.47	5.76–8.95	06	03	C, ER, M, N

AA: amino acids; C: cytoplasm; ER: endoplasmic reticulum; kDa: kilodalton; M: mitochondria; MD: multi-domain; MW: molecular weight; N: nucleus; pI: isoelectric point; SD: single domain.

**Table 2:** Representation of cyclophilin, FK506-binding protein (FKBP), parvulin and protein phosphatase 2A activator (PTPA) orthogroups in different *Penicillium* spp.

		Cyclophilins													FKBPs					Parvulins			PTPAs		
S. No	*Penicillium spp.*	Cyclophilins	PenCYP01	PenCYP02	PenCYP03	PenCYP04	PenCYP05	PenCYP06	PenCYP07	PenCYP08	PenCYP09	PenCYP10	PenCYP11	PenCYP12	FKBPs	PenFKBP01	PenFKBP02	PenFKBP03	PenFKBP04	Parvulins	PenPAR01	PenPIN01	PTPAs	PenPTPA01	PenPTPA02
1	*P. antarcticum*	9	✓	×	✓	×	✓	✓	✓	✓	✓	✓	✓	×	5	✓	✓*	✓	✓	1	✓	×	2	✓	✓
2	*P. arizonense*	10	✓	×	✓	✓	✓	✓	✓	✓	✓	✓	✓	×	4	✓	✓	✓	✓	1	✓	×	2	✓	✓
3	*P. brasilianumn*	10	✓	✓	✓	×	✓	✓	✓	✓	✓	✓	✓	×	4	✓	✓	✓	✓	2	✓	✓	2	✓	✓
4	*P. camemberti*	10	✓	✓	✓	✓	✓	✓	×	✓	✓	✓	✓	×	4	✓	✓	✓	✓	2	✓	✓	2	✓	✓
5	*P. chrysogenum*	10	✓	✓	✓	✓	✓	✓	×	✓	✓	✓	✓	×	4	✓	✓	✓	✓	2	✓	✓	2	✓	✓
6	*P. coprophilum*	8	✓	✓	×	✓	✓	✓	×	×	×	✓	✓	✓	4	✓	✓	✓	✓	1	✓	×	2	✓	✓
7	*P. decumbens*	7	✓	✓	✓	✓	✓	✓	×	×	×	×	✓	×	2	×	✓	✓	×	2	✓	✓	2	✓	✓
8	*P. digitatum*	11	✓	✓	✓	✓	✓	✓	✓	✓	✓	✓	✓	×	4	✓	✓	✓	✓	2	✓	✓	2	✓	✓
9	*P. expansum*	10	✓	✓	✓	✓	✓	✓	×	✓	✓	✓	✓	×	4	✓	✓	✓	✓	2	✓	✓	2	✓	✓
10	*P. flavigenum*	9	✓	✓	✓	✓	✓	✓	×	✓	×	✓	✓	×	4	✓	✓	✓	✓	2	✓	✓	2	✓	✓
11	*P. freii*	11	✓	✓	✓	✓	✓	✓	✓	✓	✓	✓	✓	×	4	✓	✓	✓	✓	2	✓	✓	2	✓	✓
12	*P. griseofulvum*	10	✓	✓	×	✓	✓	✓	×	✓	✓	✓	✓	✓	4	✓	✓	✓	✓	2	✓	✓	2	✓	✓
13	*P. italicum*	10	✓	✓	✓	✓	✓	✓	×	✓	✓	✓	✓	×	4	✓	✓	✓	✓	2	✓	✓	2	✓	✓
14	*P. nalgiovense*	11	✓	✓	✓	✓	✓	✓	✓	✓	✓	✓	✓	×	4	✓	✓	✓	✓	2	✓	✓	1	✓	×
15	*P. nordicum*	11	✓	✓	✓	✓	✓	✓	✓	✓	✓	✓	✓	×	4	✓	✓	✓	✓	2	✓	✓	2	✓	✓
16	*P. occitanis*	10	✓	✓	✓	×	✓	✓	✓	✓	✓	✓	✓	×	3	×	✓	✓	✓	2	✓	✓	2	✓	✓
17	*P. oxalicum*	10	✓	✓	✓	×	✓	✓	✓	✓	✓	✓	✓	×	4	✓	✓	✓	✓	2	✓	✓	2	✓	✓
18	*P. polonicum*	8	✓	✓	×	✓	✓	✓	×	×	✓	✓	✓	×	4	✓	✓	✓	✓	2	✓	✓	2	✓	✓
19	*P. roqueforti*	11	✓	✓	✓	✓	✓	✓	✓	✓	✓	✓	✓	×	4	✓	✓	✓	✓	2	✓	✓	2	✓	✓
20	*P. rubens*	11	✓	✓	✓	✓	✓	✓	✓	✓	✓	✓	✓	×	4	✓	✓	✓	✓	2	✓	✓	2	✓	✓
21	P. solitum	10	✓	✓	✓	✓	✓	✓	×	✓	✓	✓	✓	×	4	✓	✓	✓	✓	1	✓	×	2	✓	✓
22	*P. steckii*	11	✓	✓	✓	✓	✓	✓	✓	✓	✓	✓	✓	×	3	×	✓	✓	✓	2	✓	✓	1	✓	×
23	*P. subrubescens*	10	✓	✓	✓	×	✓	✓	✓	✓	✓	✓	✓	×	4	✓	✓	✓	✓	2	✓	✓	2	✓	✓
24	*P. vulpinum*	9	✓	✓	✓	✓	✓	✓	×	✓	×	✓	✓	×	4	✓	✓	✓	✓	2	✓	✓	2	✓	✓

×: absent; ✓: present; *: two copies.

The predicted molecular weights (MWs) and pIs of the *Penicillium* cyclophilins range between 17.63 kDa (*P. steckii*) to 126.82 kDa (*P. arizonense*)*,* and 4.56 (*P. expansum*) to 9.32 (*P. steckii*), respectively (Table 1). The cyclophilins in *P. oxalicum* also showed divergence in their MWs and pIs, with the values ranging between 17.79 kDa (PoxCYP17) to 69.82 kDa (PoxCYP69), and 5.93 (PoxCYP41) to 8.87 (PoxCYP18), respectively (Supplementary Table S1). Though predominantly cytosolic, the cyclophilins in *Penicillium* were also predicted to localize to the nucleus, endoplasmic reticulum (ER) and mitochondria, highlighting functional divergence of these proteins (Table 1; Supplementary Table S2a-l). Besides cytosolic, the ER-localized (PoxCYP23) and nuclear PPIases (PoxCYP54, PoxCYP62-1, PoxCYP62-2 and PoxCYP69) were also observed in *P. oxalicum* (Supplementary Table S1). Except for PenCYP07 cyclophilins, in which the CLD ranges between 128 and 179 AAs, this domain's length is similar in cyclophilins of all other orthogroups (Supplementary Table S2a-l). The secondary structure of CLD, comprising of a typical β-barrel of eight antiparallel β-sheets with the two ends closed by α-helices and represented as βIβIIαIβIIIβIVβVβVIαIIβVIIαIIIβVIII in hCYPA (Supplementary Fig. S2)^24^, showed conservation in cyclophilins of all orthogroups except PenCYP03, PenCYP05, PenCYP11 and PenCYP12 which either lack or contain a partial β1 region. Based on the presence of domains other than CLD, the cyclophilins were further classified as single domain (SD) or multidomain (MD) proteins (Table 3). While seven orthogroups (PenCYP01-PenCYP06 and PenCYP09) consist of SD cyclophilins, five orthogroups (PenCYP07, PenCYP08, PenCYP10-PenCYP12) comprise of MD proteins that contain additional domains such as TPR, RRM, U-box, WD, PP2C, and GIT_SDH (Supplementary Table S2a-l). Both SD (6) and MD cyclophilins (4) were also observed in *P. oxalicum* (Supplementary Table S1). This study predicted 15 different motifs within and outside the CLD (Supplementary Fig. S1), the motif composition being conserved in different cyclophilins of the same orthogroup. Comparative analysis with hCYPA revealed that all the active site residues corresponding to Arg (55), Phe (60), Met (61), Gln (63), Ala (101), Phe (113), Trp (121), Leu (122) and His (126), essential for PPIase activity and CsA interaction, are conserved in all cyclophilins of orthogroups PenCYP03, PenCYP06, PenCYP11 and PenCYP12 (Table 4, Supplementary Fig. S2). In *P. oxalicum* also, the PoxCYP17, PoxCYP18, PoxCYP23 and PoxCYP69 proteins showed retention of all the active site residues (Supplementary Table S4). Thus, these proteins are likely to be enzymatically active and might perform different cellular functions due to their PPIase activity. On the contrary, the cyclophilins belonging to the groups PenCYP02, PenCYP04, PenCYP05 and PenCYP07-PenCYP10 exhibited several substitutions in their active site residues, with the most common being Trp (121)/His (126) replaced with other residues (Table 4, Supplementary Fig. S2). While Trp121 in hCYPA is essential for CsA binding and changes in this residue result in decreased sensitivity to this immunosuppressant, mutations in the other active site residues are known to result in alteration in the PPIase activity^45–48^. The effect of alterations in the active site residues on PPIase activity of these cyclophilins needs further evaluation by cloning and characterizing these proteins.

**Table 3:** Domain architecture and localization of different orthogroups of cyclophilins, FK506-binding proteins (FKBPs), parvulins and protein phosphatase 2A activators (PTPAs) in *Penicillium* spp.

Orthogroups	Proteins	SD	MD	Domains	Localization
**Cyclophilins**					
PenCYP01	24	24	0	CLD (24)	C
PenCYP02	22	22	0	CLD (22)	C
PenCYP03	21	21	0	CLD (21)	C (14), M (7)
PenCYP04	19	19	0	CLD (19)	C
PenCYP05	24	24	0	CLD (24)	C
PenCYP06	24	24	0	CLD (24)	ER
PenCYP07	13	0	13	CLD + TPR (13)	C
PenCYP08	21	0	21	CLD + RRM (21)	N
PenCYP09	20	20	0	CLD (20)	N
PenCYP10	23	0	23	CLD + U-box (22)	N
				CLD + U-box + PP2C (1)	
PenCYP11	24	0	24	CLD + WD REPEAT (24)	N
PenCYP12	2	0	2	CLD + GIT_SDH (2)	C
**FKBPs**					
PenFKBP01	21	21	0	FKBP (21)	C
PenFKBP02	25	25	0	FKBP (21)	C
PenFKBP03	24	24	0	FKBP (21)	ER
PenFKBP04	23	0	23	FKBP + NPL (23)	N
**PARs**					
PenPAR01	24	24	0	PPIase (24)	C (14), M (1), N (9)
PenPIN01	20	0	20	PPIase + WW (20)	C (2), N (18)
**PTPAs**					
PenPTPA01	24	24	0	PTPA (24)	C
PenPTPA02	22	22	0	PTPA (22)	C

C: cytoplasm; CLD: cyclophilin-like domain; ER: endoplasmic reticulum; FKBP: FK506-binding protein; GIT_SDH: Spa2 homology domain (SHD) of GIT [G-protein-coupled receptor (GPCR)-kinase-interacting protein]; M: mitochondria; MD: multi-domain; N: nucleus; NPL: nucleoplasmin-like domain; PP2C: Protein phosphatase 2C; PTPA: protein phosphatase 2A activator; RRM: RNA recognition motif; SD: single domain; TPR: tetratricopeptide repeat; U-box: U-box domain; WD: tryptophan-aspartate repeats; WW: Domain with 2 conserved tryptophan residues.

**Table 4:** Conservation of the active site residues with respect to the human orthologues hCYPA, hFKBP12 and hPIN1/hPAR14, respectively, in the cyclophilins, FK506-binding proteins (FKBPs) and parvulins of different orthogroups in different *Penicillium* spp.

Cyclophilins														
Orthogroups	Conserved ASRs	ARG (R) 55	PHE (F) 60	MET (M) 61	GLN (Q) 63	ALA (A) 101	PHE (F) 113	TRP (W) 121	LEU (L) 122	HIS (H) 126				
PenCYP01	8	R	F	M	Q	A	F/Y	W	L	H				
PenCYP02	8	R	F	M	Q	A	F	F	L	H				
PenCYP03	9	R	F	M	Q	A	F	W	L	H				
PenCYP04	8	R	F	M	Q	A	F	H	L	H				
PenCYP05	7	R	F	M	Q	A	F	H	L	S				
PenCYP06	9	R	F	M	Q	A	F	W	L	H				
PenCYP07	8	R	F	M	Q	A	F	H	L	H				
PenCYP08	5	R	F	T/S	Q	A	I/L	Y	L	A				
PenCYP09	5	R	F	V	Q	A	F	R	L/M	C				
PenCYP10	8	R	F	M	Q	A	F	H	L	H				
PenCYP11	9	R	F	M	Q	A	F	W	L	H				
PenCYP12	9	R	F	M	Q	A	F	W	L	H				

FKBPs														
Orthogroups	Conserved ASRs	TYR (Y) 27	PHE (F) 37	ASP (D) 38	ARG (R) 43	PHE (F) 47	PHE (F) 49	Gln (Q) 54	GLU (E) 55	ILE (I) 57	TRP (W) 60	TYR (Y) 83	HIS (H) 88	PHE (F) 100
PenFKBP01	6	Y	F	D	P	F/L	V	G	K/Q	I /-	W	Y	-	F
PenFKBP02	8	Y	F	D	R	F/L	S/T	G	R	I	W	Y	F/Y	F
PenFKBP03	9	Y	F	D	R	L	F	G	R	I	W	Y	I/V/M	F
PenFKBP04	10	Y	F	D	K	F	F	G	E	I	W	Y	L	F

Parvulins														
Parvulins	Conserved ASRs	HIS (H) 59	LYS (K) 63	ARG (R) 68	ARG (R) 69	CYS (C)/ ASP (D)* 113	LEU (L) 122	MET (M) 130	PHE (F) 134	SER (S)/ PHE (F)* 154	HIS (H) 157			
PenPAR01(vs hPAR14)	5	H	#	#	#	D	L	L	F	F/H	H			
PenPIN01 (vs hPIN1)	10	H	K	R	R	C	L	M	F	S	H			

*: residues present in hPAR14; #: residues absent in hPAR14.

The phylogenetic relationship among different cyclophilins was studied by constructing an unrooted tree based on proteins consisting of full-length or partial CLD sequences. This analysis divided the *Penicillium* cyclophilins into 11 distinct groups, A-K (Fig. 2). Interestingly, no *P. oxalicum* cyclophilin was observed in group G, suggesting that this gene might have been acquired by other species or lost from *P. oxalicum* during the course of evolution. Similar events were implicated earlier in the evolution of plant NAC gene family also^49^. A noteworthy feature of Group K, comprising of PenCYP07 orthogroup, is the presence of PcaCYP7, PexCYP8, PitCYP8, PgrCYP7, and PsoCYP7 (that contain only N-terminus CLD) along with PcaCYP33, PexCYP33, PitCYP33, PgrCYP33 and PsoCYP33 (which possess only C-terminus CLD). It is likely that PcaCYP7, PexCYP8, PitCYP8, PgrCYP7 and PsoCYP7 might be the result of deletion of N-terminus region of CLD in PcaCYP33, PexCYP33, PitCYP33, PgrCYP33 and PsoCYP33, respectively. This speculation is supported by the fact that pairwise alignment of PcaCYP7, PexCYP8, PgrCYP7, PitCYP8, and PsoCYP7 with PcaCYP33, PexCYP33, PgrCYP33, PitCYP33, and PsoCYP33, respectively, corresponded to full-length cyclophilins that are homologous to other members of the same group (Supplementary Fig. S3). Though PgrCYP121 and PcoCYP121 were clustered in Group I, pairwise comparison prompted us to designate these proteins as a separate orthogroup PenCYP12 due to the presence of a large stretch of 950 AA residues that was not observed in other members of this group. The two proteins depicted 97% and 94% similarity in their GIT_SDH and CLD domains. Interestingly, GIT_SDH domain has not been reported yet in any of the *Penicillium* cyclophilins.

#### FK506-binding proteins (FKBPs)

**Figure 3:**
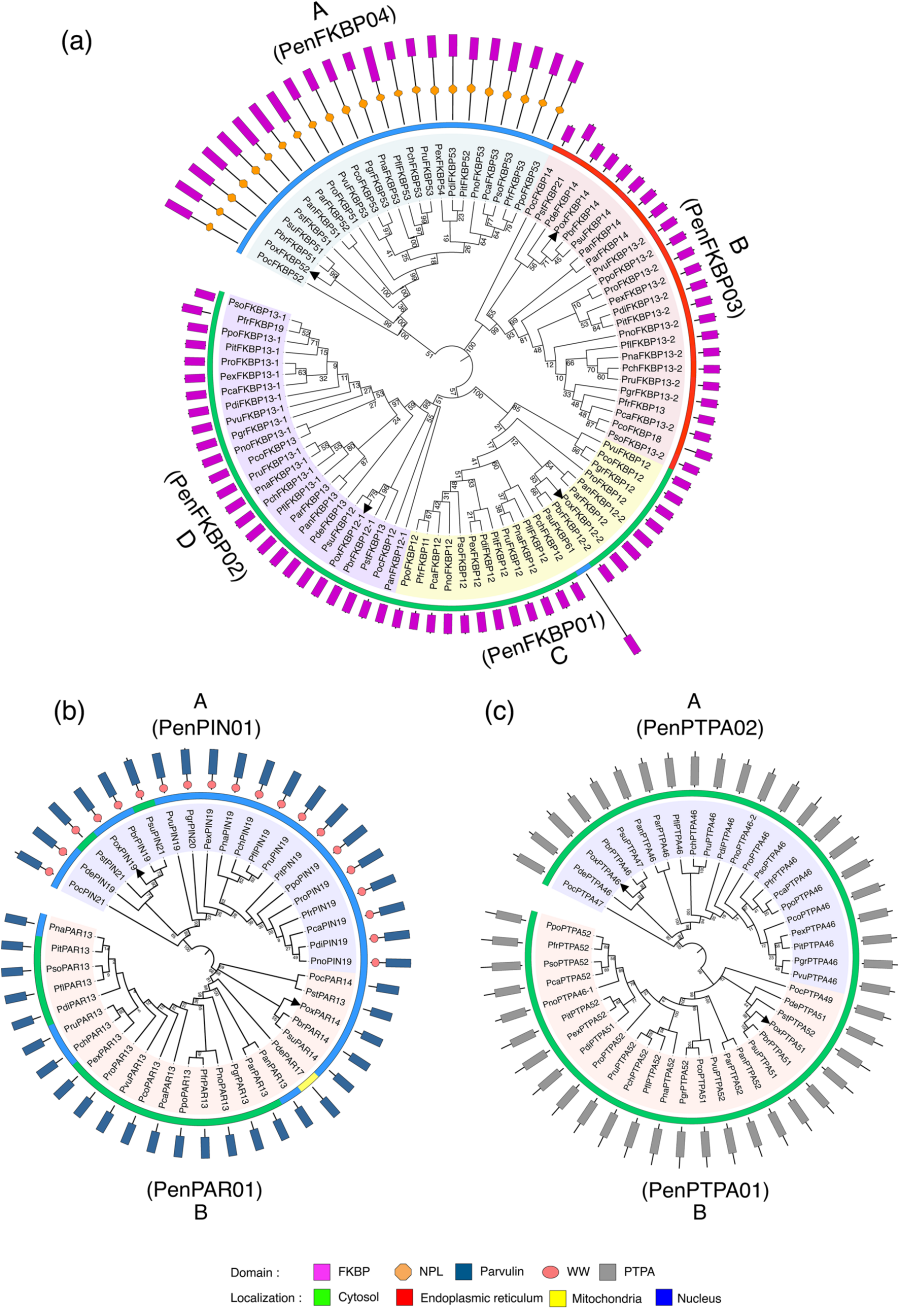
The phylogenetic relationship of different *Penicillium* FKBPs (**a**), parvulins (**b**), and protein phosphatase 2A phosphatase activators (PTPAs) (**c)**. The unrooted tree was generated using the neighbor-joining method in MEGA X (v10.1.7) software (http://www.megasoftware.net). The constructed trees were further annotated with the Iterative Tree of Life (http://itol.embl.de/) and redrawn by using Adobe Illustrator (v25.2.3) (https://adobe.com/products/illustrator). Bootstrap values from 1000 replicates are indicated at each branch. Localization of each member is represented in different colors. Domain architecture of each protein is also depicted in the outermost layer. Black triangles represent the *Penicillium oxalicum*.

Ninety-three putative FKBPs were identified in *Penicillium* spp*.* by basic local alignment search tool (BLAST) analysis using the human FKBP, hFKBP12, as a query. The hFKBP12 is the smallest member (12 kDa) of the FKBP family and contains the PPIase core domain^50,51^. Based on similarity, these proteins were categorized into four different orthogroups viz., PenFKBP01, PenFKBP02, PenFKBP03 and PenFKBP04 (Table 2). This grouping was also supported by the phylogenetic analysis, which depicted a close relationship of these proteins within a group (Fig. 3a). All *Penicillium* spp. except *P. decumbens*, *P. occitanis* and *P. steckii* depicted four different FKBPs. While *P. decumbens* contains only two FKBPs*,* both *P. occitanis* and *P. steckii* consist of three each (Table 2). Interestingly, *P. antarcticum* exhibited two different PenFKBP02 proteins, PanFKBP12-1 and PanFKBP13, that are 72.1% similar and appear to be paralogous (Supplementary Table S5b). The presence of PenFKBP02 and PenFKBP03 FKBPs in all *Penicillium* spp. underlines their essential role in the cell. The number of introns in FKBP genes varies between 1 and 5, with PsuFKBP61 of the orthogroup PenFKBP01 being the only exception with seven introns (Supplementary Fig. S4). Except for PsuFKBP61, the intron–exon architecture showed conservation in the FKBP genes of the same orthogroup. The FKBPs of orthogroup PenFKBP02 showed highest similarity with hFKBP12 (58–71.3%), followed by PenFKBP01 (13.4–61.2%), PenFKBP03 (36.4–51.1%), and PenFKBP04 (14.8–16%) (Supplementary Table S5a-d). Of the different *P. oxalicum* FKBPs, the maximum similarity with hFKBP12 was observed for PoxFKBP12-1 (64.5%), followed by PoxFKBP12-2 (61.2%), PoxFKBP14 (51.1%) and PoxFKBP52 (15.1%) (Supplementary Table S5e). The similarity among different FKBPs in *P. oxalicum* ranges between 15.3 and 73.6% (Supplementary Table S5e).

Interspecific variability observed in the MWs and pIs of FKBPs in each orthogroup in *Penicillium* suggests divergence (Table 5, Supplementary Table S6a–d). The MWs of FKBPs in *P. oxalicum* differ from 12.93 to 52.72 kDa, with a pI range of 4.38–9.36 (Table S1). The FKBPs in *Penicillium* spp. were predicted to localize to different subcellular organelles. While members of the orthogroups PenFKBP01 and PenFKBP02 might localize to the cytosol, the PenFKBP03 and PenFKBP04 FKBPs are likely to be present in the ER and nucleus, respectively (Table 3). An ER retention sequence (KDEL) (Supplementary Fig. S5) might be responsible for the likely presence of PenFKBP03 proteins in the ER. Contrary to the PenFKBP01, PenFKBP02 and PenFKBP03 proteins, which consist of only FKBP domain, the PenFKBP04 members also exhibited a nucleoplasmin like (NPL) domain (Table 3). The FKBP domain, consisting of four to six antiparallel beta-sheets surrounding the alpha-helix and represented as βIβIIβIIIαIβIVαIIβVαIIIβVI in hFKBP12^24^, is conserved in all *Penicillium* FKBPs except for few members of orthogroup PenFKBP01 that lack the β1-sheet (Supplementary Fig. S6). Of the 15 different motifs observed in *Penicillium* FKBPs, the motifs 1 and 3, part of the FKBP domain, were observed in all the proteins. (Supplementary Fig. S4). Comparison of the 13 key residues which are implicated in FK506-binding^52^ revealed that relative to hFKBP12, the members of orthogroups PenFKBP01, PenFKBP02, PenFKBP03 and PenFKBP04 showed conservation at six, eight, nine and ten positions respectively (Table 4, Supplementary Fig. S6).

**Table 5:** Genome-wide analysis of FK506-binding proteins (FKBPs) in different *Penicillium* spp.

S. No	*Penicillium spp.*	Genes	Proteins	AA residues	MW (kDa)	pI	SD	MD	Subcellular localization
1	*P. antarcticum*	05	05	115–478	12.33–51.91	4.34–9.36	04	01	C, ER, N
2	*P. arizonense*	04	04	121–479	12.89–52.05	4.34–9.36	03	01	C, ER, N
3	*P. brasilianumn*	04	04	121–478	12.87–51.85	4.35–9.36	03	01	C, ER, N
4	*P. camemberti*	04	04	121–487	12.92–53.24	4.40–9.36	03	01	C, ER, N
5	*P. chrysogenum*	04	04	121–488	12.90–53.24	4.38–9.36	03	01	C, ER, N
6	*P. coprophilum*	04	04	122–487	12.88–53.22	4.39–9.52	03	01	C, ER, N
7	*P. decumbens*	02	02	122–130	13.09–14.15	5.59–6.72	02	00	C, ER
8	*P. digitatum*	04	04	121–497	12.98–54.37	4.49–9.36	03	01	C, ER, N
9	*P. expansum*	04	04	121–494	12.95–54.12	4.44–9.36	03	01	C, ER, N
10	*P. flavigenum*	04	04	121–486	12.90–53.04	4.37–9.36	03	01	C, ER, N
11	*P. freii*	04	04	109–489	11.53–53.43	4.42–9.43	03	01	C, ER, N
12	*P. griseofulvum*	04	04	121–491	12.90–53.56	4.33–9.52	03	01	C, ER, N
13	*P. italicum*	04	04	121–483	12.98–52.83	4.83–9.36	03	01	C, ER, N
14	*P. nalgiovense*	04	04	121–489	12.87–53.25	4.35–9.36	03	01	C, ER, N
15	*P. nordicum*	04	04	121–491	12.95–53.58	4.43–9.36	03	01	C, ER, N
16	*P. occitanis*	03	03	119–478	12.77–52.10	4.33–6.57	02	01	C, ER, N
17	*P. oxalicum*	04	04	121–484	12.93–52.72	4.38–9.36	03	01	C, ER, N
18	*P. polonicum*	04	04	121–489	12.92–53.44	4.42–9.36	03	01	C, ER, N
19	*P. roqueforti*	04	04	121–475	12.89–51.70	4.43–9.36	03	01	C, ER, N
20	*P. rubens*	04	04	121–488	12.90–53.24	4.38–9.36	03	01	C, ER, N
21	*P. solitum*	04	04	121**–**492	12.92**–**53.85	4.40–9.36	03	01	C, ER, N
22	*P. steckii*	03	03	123–475	13.17–51.56	4.31–7.09	02	01	C, ER, N
23	*P. subrubescens*	04	04	121–553	12.87–61.22	4.36–6.06	02	02	C, ER, N
24	*P. vulpinum*	04	04	121–491	12.89–53.64	4.43–9.40	03	01	C, ER, N

AA: amino acids; C: cytoplasm; ER: endoplasmic reticulum; kDa: kilodalton; MD: multi-domain; MW: molecular weight; N: nucleus; pI: isoelectric point; SD: single domain.

#### Parvulins

On the basis of homology with human parvulins hPIN1 (Protein Interacting with NIMA) and hPAR14 (human parvulin 14), the *Penicillium* parvulins were grouped into two different orthogroups viz., PenPIN01 and PenPAR01, respectively (Table 2). Phylogenetic analysis also provided evidence for the evolutionary relationship of these proteins within each orthogroup (Fig. 3b). The genes encoding PenPIN01 (except PsuPIN21) and PenPAR01 showed one and two introns, respectively (Supplementary Fig. S7). Whereas, PenPAR01 proteins were observed in all *Penicillium* spp. analyzed, the PenPIN01 parvulins were not detected in *P. antarcticum, P. arizonense*, *P. coprophilum* and *P. solitum* (Table 2). The PenPAR01 and PenPIN01 parvulins showed 53.7%-68.5%, and 57.7%-66.7%, similarity with hPAR14 and hPIN1, respectively (Supplementary Table S7a, b). In *P. oxalicum* also, the PoxPAR14 and PoxPIN1 shared 66.9% and 65.7% similarity with their human orthologues hPAR14 and hPIN1, respectively. High similarity among members of PenPAR01 (65.4–100%) and PenPIN01 (76.5–100%) groups implies conservation of parvulins among different species of *Penicillium* (Supplementary Table S7a, b). Except for PdePAR17 (17.67 kDa) in *P. decumbens*, the MWs of PenPAR01 proteins differed between 13.62 and 14.75 kDa, and that of PenPIN01 members between 19.30 and 21.88 kDa (Table 6). The pI values in orthogroups PenPAR01 and PenPIN01 varied between 9.41 to 9.68, and 5.72 to 6.46, respectively. The larger size of PdePAR17 is attributed to an extended N terminal 36 amino acid sequence containing mitochondrial localization signal^53^. Except for PdePAR17, which might be a mitochondrial protein, all members of the PenPAR01 orthogroup were predicted to localize to either cytosol or nucleus. Majority of the PenPIN01 proteins, on the contrary, appeared to be nuclear, with only *P. steckii* (PstPAR13) and *P. subrubescens* (PsuPAR14) depicting localization in the cytoplasm. Contrary to the PenPAR01 parvulins, that contain only the PPIase domain, the PenPIN01 members also exhibited an additional conserved N-terminal WW domain (Table 3). The PenPAR01 and PenPIN01 parvulins contain ten different motifs, with the motifs 1, 4 and 5 present in all members (Supplementary Fig. S7). Whereas all the ten active site residues relative to hPIN1 are conserved in PenPIN01 parvulins, only five active site residues in PenPAR02 members showed conservation relative to hPAR14 (Table 4). As observed in hPAR14 and hPIN1^54^, all *Penicillium* parvulins exhibited the presence of β1α1α2α3β2α4β3β4 elements in their PPIase domain (Supplementary Fig. S7a, b), suggesting that the secondary structure of these proteins is conserved across taxa. Though conservation of these proteins underlines their fundamental role in the cell, the absence of PenPIN01 members in *P. antarcticum, P. arizonense, P. coprophilum* and *P. solitum* also suggests redundancy in their functions.

**Table 6:** Genome-wide analysis of parvulins in different species of *Penicillium*.

		Orthogroup PenPAR01						Orthogroup PenPIN01					
S. No	*Penicillium spp.*	Protein name	Protein accession no	Total AAs/ (Rotamase domain)	MW (kD)	pI	Loc	Protein name	Protein accession no	Total AAs/ (WW domain)/ (PPIase domain)	MW (kDa)	pI	Loc
1	*P. antarcticum*	PanPAR13	OQD87118.1	127/(35–127)	13.62	9.52	N	–	–	–	–	–	–
2	*P. arizonense*	ParPAR13	XP_022493562.1	127/(35–127)	13.64	9.52	C	–	–	–	–	–	–
3	*P. brasilianumn*	PbrPAR14	CEJ54268.1	130/(38–130)	14.17	9.45	N	PbrPIN19	CEJ61044.1	176/(5–38)/(67–175)	19.44	6.14	N
4	*P. camemberti*	PcaPAR13	CRL17938.1	129/(37–129)	13.85	9.49	C	PcaPIN19	CRL25274.1	176/(5–38)/(67–175)	19.58	5.72	N
5	*P. chrysogenum*	PchPAR13	KZN91971.1	129/(37–129)	13.80	9.49	N	PchPIN19	KZN92659.1	176/(5–38)/(67–175)	19.55	5.89	N
6	*P. coprophilum*	PcoPAR13	OQE46467.1	129/(37–129)	13.74	9.49	C	–	–	–	–	–	–
7	*P. decumbens*	PdePAR17	OQD75108.1	129/(70–162)	17.67	9.68	M	PdePIN19	OQD67334.1	176/(5–38)/(67–175)	19.52	6.32	N
8	*P. digitatum*	PdiPAR13	XP_014531496.1	129/(37–129)	13.79	9.49	C	PdiPIN19	XP_014532624.1	176/(5–38)/(67–175)	19.56	5.72	N
9	*P. expansum*	PexPAR13	XP_016601110.1	129/(37–129)	13.79	9.49	C	PexPIN19	XP_016603179.1	176/(5–38)/(67–175)	19.62	5.72	N
10	*P. flavigenum*	PflPAR13	OQE30197.1	129/(37–129)	13.79	9.49	C	PflPIN19	OQE19709.1	176/(5–38)/(67–175)	19.55	5.89	N
11	*P. freii*	PfrPAR13	KUM64167.1	128/(36–128)	13.66	9.52	C	PfrPIN19	KUM58880.1	176/(5–38)/(67–175)	19.60	5.72	N
12	*P. griseofulvum*	PgrPAR13	KXG47026.1	129/(37–129)	13.79	9.49	C	PgrPIN20	KXG54614.1	181/(5–38)/(68–175)	20.13	5.90	N
13	*P. italicum*	PitPAR13	KGO71764.1	129/(37–129)	13.79	9.49	C	PitPIN19	KGO73822.1	176/(5–38)/(67–175)	19.52	5.72	N
14	*P. nalgiovense*	PnaPAR13	OQE86291.1	129/(37–129)	13.77	9.49	N	PnaPIN19	OQE93697.1	174/(5–38)/(67–174)	19.30	5.82	N
15	*P. nordicum*	PnoPAR13	KOS48877.1	128/(36–128)	13.67	9.52	C	PnoPIN19	KOS38539.1	176/(5–38)/(67–175)	19.56	5.72	N
16	*P. occitanis*	PocPAR14	PCH00892.1	133/(37–133)	14.75	9.48	N	PocPIN21	PCH00362.1	194/(6–39)/(67–174)	21.88	6.46	N
17	*P. oxalicum*	PoxPAR14	EPS27836.1	128/(36–128)	14.00	9.57	N	PoxPIN19	EPS29250.1	175/(5–38)/(67–174)	19.43	5.98	N
18	*P. polonicum*	PpoPAR13	OQD72015.1	128/(36–128)	13.66	9.52	C	PpoPIN19	OQD71617.1	176/(5–38)/(67–175)	19.59	5.72	N
19	*P. roqueforti*	ProPAR13	CDM33780.1	129/(37–129)	13.79	9.49	C	ProPIN19	CDM27042.1	176/(5–38)/(67–175)	19.59	5.72	N
20	*P. rubens*	PruPAR13	XP_002562081.1	129/(37–129)	13.80	9.49	N	PruPIN19	KAF3031078.1	176/(5–38)/(67–175)	19.55	5.89	N
21	*P. solitum*	PsoPAR13	OQD86406.1	129/ (37–129)	13.77	9.41	C	–	–	–	–	–	–
22	*P. steckii*	PstPAR13	OQE27721.1	128/(36–128)	13.77	9.49	N	PstPIN21	OQ E20301.1	196/(26–59)/(88–195)	21.82	5.90	C
23	*P. subrubescens*	PsuPAR14	OKO93703.1	129/(37–129)	14.17	9.57	N	PsuPIN21	OKP10946.1	194/(10–43)/(72–181)	21.40	6.20	C
24	*P. vulpinum*	PvuPAR13	OQE10874.1	129/(37–129)	13.79	9.49	C	PvuPIN19	OQE02824.1	177/(5–38)/(68–176)	19.61	5.90	N

AA: amino acids; C: cytoplasm; kDa: kilodalton; Loc: subcellular localization; M: mitochondria; MW: molecular weight; N: nucleus; pI: isoelectric point; WW: Domain with 2 conserved tryptophan residues.

#### Protein phosphatase 2A phosphatase activators (PTPAs)

The members of PenPTPA01 and PenPTPA02 orthogroups in *Penicillium* spp. were identified by BLAST analysis based on their similarity with their yeast orthologues YPA1 and YPA2, respectively. This analysis revealed that except for *P. nalgiovense* and *P. steckii*, which lack PTPA02 gene, all other *Penicillium* species contain both the PTPAs (Tables 2, 7). Phylogenetic analysis also supported a close evolutionary relationship among proteins of each orthogroup (Fig. 3c). In silico studies further revealed that while all the genes encoding PenPTPA01 proteins contain two introns, the same is lacking in the PenPTPA02 genes (Supplementary Fig. S9). The YPA1 exhibited 44.3–49.2% similarity with PenPTPA01 orthologues, compared to 53.1–57.4% for YPA2 with PenPTPA02 members (Supplementary Table S8a, b). The molecular weights of PenPTPA01 and PenPTPA02 vary between 46.43 to 52.97 kDa, and 46.05 to 47.55 kDa, respectively, while the pI values for the two PPIases range between 5.81–7.20 and 5.84–6.44, respectively (Table 7). The PenPTPA01 and PenPTPA02 proteins in *Penicillium* spp. were predicted to localize to the cytosol, and consist of only PTPA domain of 283–331 and 293–295 amino acid residues, respectively. The two PTPA orthogroups revealed the presence of 15 different motifs, of which six (1–3, 6 and 9) are common to all members (Supplementary Fig. S9). High similarity among PenPTPA01 (69.3–100%) and PenPTPA02 (77.1–100%) members in *Penicillium* spp. suggests conservation, indicating an essential role for these proteins in the cell (Supplementary Table S8a, b).

**Table 7:** Genome-wide analysis of protein phosphatase 2A activators (PTPAs) in different species of *Penicillium*.

		Orthogroup PenPTPA01						Orthogroup PenPTPA02					
S. No	*Penicillium spp.*	Protein name	Protein accession no	Total AAs/ (PTPA domain)	MW (kDa)	pI	Loc	Protein name	Protein accession no	Total AAs / (PTPA domain)	MW (kDa)	pI	Loc
1	*P. antarcticum*	PanPTPA52	OQD82836.1	484/(25–356)	52.43	6.43	C	PanPTPA46	OQD82187.1	415/(54–347)	46.05	6.19	C
2	*P. arizonense*	ParPTPA52	XP_022485488.1	484/(25–356)	52.33	6.38	C	ParPTPA46	XP_022491893.1	415/(54–349)	46.13	6.19	C
3	*P. brasilianumn*	PbrPTPA51	OOQ86678.1	480/(25–356)	51.87	5.97	C	PbrPTPA46	OOQ83450.1	421/(59–354)	46.79	6.07	C
4	*P. camemberti*	PcaPTPA52	CRL24247.1	485/(22–352)	52.54	6.22	C	PcaPTPA46	CRL22781.1	419/(58–353)	46.37	6.25	C
5	*P. chrysogenum*	PchPTPA52	KZN85308.1	487/(24–354)	52.96	6.85	C	PchPTPA46	KZN84036.1	419/(58–353)	46.30	6.19	C
6	*P. coprophilum*	PcoPTPA51	OQE46369.1	476/(24–354)	51.84	6.61	C	PcoPTPA46	OQE41359.1	420/(59–354)	46.54	6.39	C
7	*P. decumbens*	PdePTPA51	OQD67240.1	473/(25–356)	51.20	5.81	C	PdePTPA46	OQD78624	415/(57–351)	46.03	6.00	C
8	*P. digitatum*	PdiPTPA51	XP_014532126.1	474/(22–352)	51.49	7.20	C	PdiPTPA46	XP_014536235.1	419/(58–353)	46.34	6.16	C
9	*P. expansum*	PexPTPA52	XP_016598742.1	485/(22–352)	52.82	6.85	C	PexPTPA46	XP_016596123.1	419/(58–353)	46.31	6.44	C
10	*P. flavigenum*	PflPTPA52	OQE29046.1	487/(24–354)	52.90	6.43	C	PflPTPA46	OQE18147.1	419/(58–353)	46.26	6.11	C
11	*P. freii*	PfrPTPA52	KUM61112.1	485/(22–352)	52.80	7.10	C	PfrPTPA46	KUM59294.1	419/(58–353)	46.39	6.34	C
12	*P. griseofulvum*	PgrPTPA52	KXG48326.1	487/(24–354)	52.78	6.38	C	PgrPTPA46	KXG49099.1	419/(58–353)	46.30	6.31	C
13	*P. italicum*	PitPTPA52	KGO74783.1	485/(22–352)	52.97	6.85	C	PitPTPA46	KGO75264.1	419/(58–353)	46.46	6.34	C
14	*P. nalgiovense*	PnaPTPA52	OQE84955.1	488/(24–354)	52.93	6.61	C	–	–	–	–	–	–
15	*P. nordicum*	PnoPTPA46-1	KOS46416.1	431/(15–298)	46.43	6.33	C	PnoPTPA46-2	KOS43152.1	419/(58–353)	46.60	6.04	C
16	*P. occitanis*	PocPTPA49	PCH02323.1	449/(19–350)	49.74	6.08	C	PocPTPA47	PCG91093.1	424/(61–354)	47.55	5.84	C
17	*P. oxalicum*	PoxPTPA51	EPS34045.1	478/(25–356)	51.78	5.97	C	PoxPTPA46	EPS25240.1	422/(60–355)	46.80	5.92	C
18	*P. polonicum*	PpoPTPA52	OQD61354.1	485/(22–352)	52.68	6.28	C	PpoPTPA46	OQD61354.1	419/(58–353)	46.35	6.44	C
19	*P. roqueforti*	ProPTPA52	CDM34519.1	482/(23–353)	52.40	6.19	C	ProPTPA46	CDM28648.1	417/(58–353)	46.14	6.25	C
20	*P. rubens*	PruPTPA52	XP_002557947.1	487/(24–354)	52.96	6.85	C	PruPTPA46	XP_002559612.1	419/(58–353)	46.30	6.19	C
21	*P. solitum*	PsoPTPA52	OQE03232.1	485/(22–352)	52.76	6.23	C	PsoPTPA46	OQD95247.1	419/(22–352)	46.38	6.34	C
22	*P. steckii*	PstPTPA52	OQE16418.1	483/(25–356)	52.62	5.87	C	–	–	–	–	–	–
23	*P. subrubescens*	PsuPTPA51	OKP02397.1	479/(25–356)	51.86	6.34	C	PsuPTPA47	OKP06461.1	420/(58–353)	47.00	6.11	C
24	*P. vulpinum*	PvuPTPA52	OQE05701.1	485/(24–354)	52.88	6.33	C	PvuPTPA46	OQE00347.1	419/(58–353)	46.48	6.13	C

AA: amino acids; C: cytoplasm; kDa: kilodalton; Loc: subcellular localization; MW: molecular weight; pI: isoelectric point.

### Estimation of PPIase activity and expression analysis of PPIases genes in *P. oxalicum*

**Figure 4:**
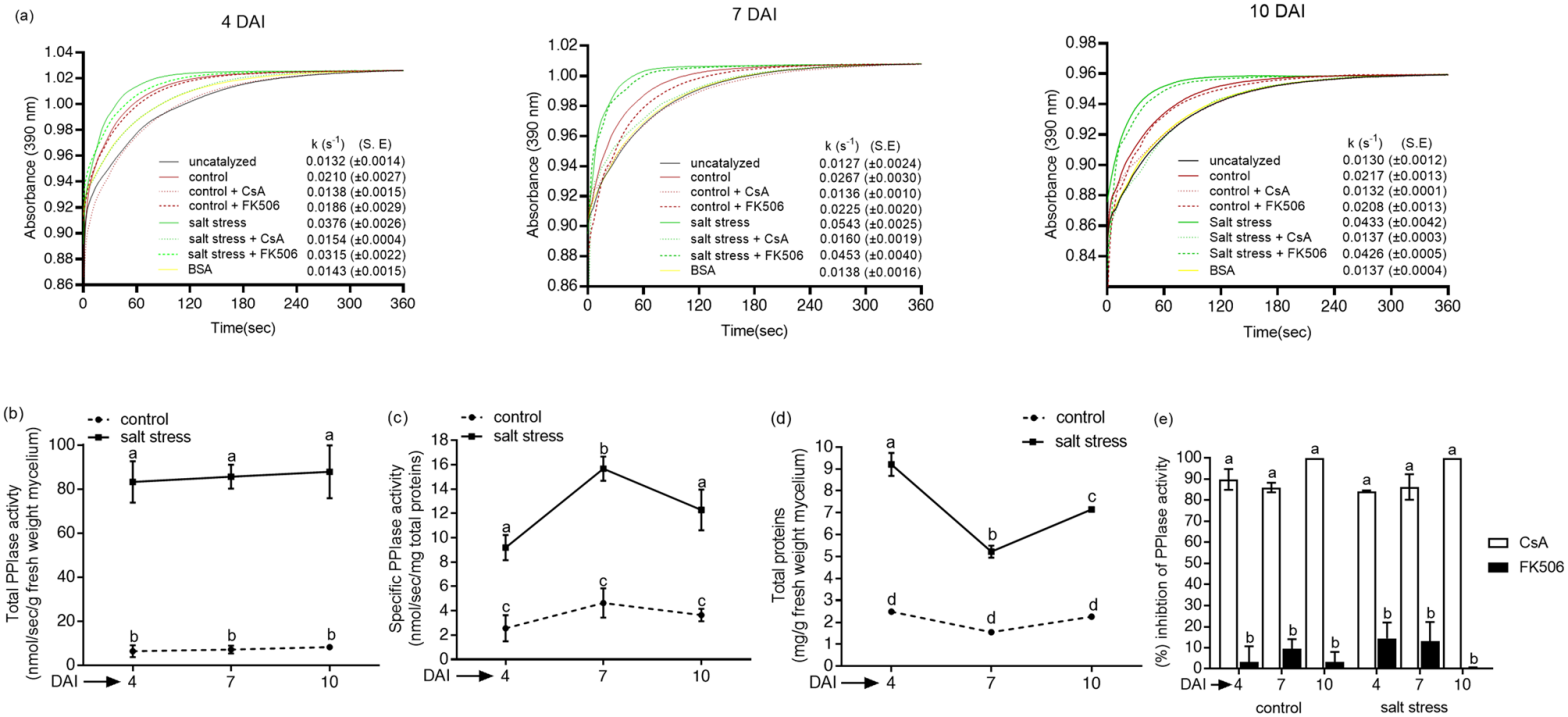
**(a)** Representative spectra showing the effect of salt stress (15% NaCl) on the mycelial peptidyl-prolyl *cis–trans* isomerase (PPIase) activity of *Penicillium oxalicum* at 4, 7 and 10 days after inoculation (DAI). The PPIase activity in the crude mycelial extracts was estimated by studying the rate of chymotrypsin catalyzed cleavage of the test peptide, and expressed as the first-order rate constant, k (s^–1^). Higher first-order rate constant signified greater PPIase activity in the extract. 20 μg of total proteins was used for this assay, with bovine serum albumin (BSA) as a negative control. The cyclophilin- and FK506-binding protein (FKBP)-associated PPIase activity was estimated by the extent of inhibition in the presence of 100 nM cyclosporin A (CsA) and 2 μM FK506, respectively. Control refers to the cultures grown in Sabouraud medium without supplementing with NaCl. (**b)** Changes in the total PPIase activity (nmol/sec/g fresh weight mycelium) and **(c)** specific PPIase activity (nmol/sec/mg total proteins), and **(d)** total soluble proteins (mg/g fresh weight mycelium) in the mycelia of *Penicillium oxalicum* grown in the presence or absence of salt. **(e)** Effect of specific inhibitors of cyclophilins (CsA) and FKBPs (FK506) on mycelial PPIase activity. The percent inhibition of PPIase activity is expressed with respect to the uninhibited control. The values depict the mean of three biological replicates ± standard error and are significant at P ≤ 0.001 (Tukey-HSD test; α = 0.05). The values with distinct letters are significantly different. (DAI: days after inoculation).

**Figure 5:**
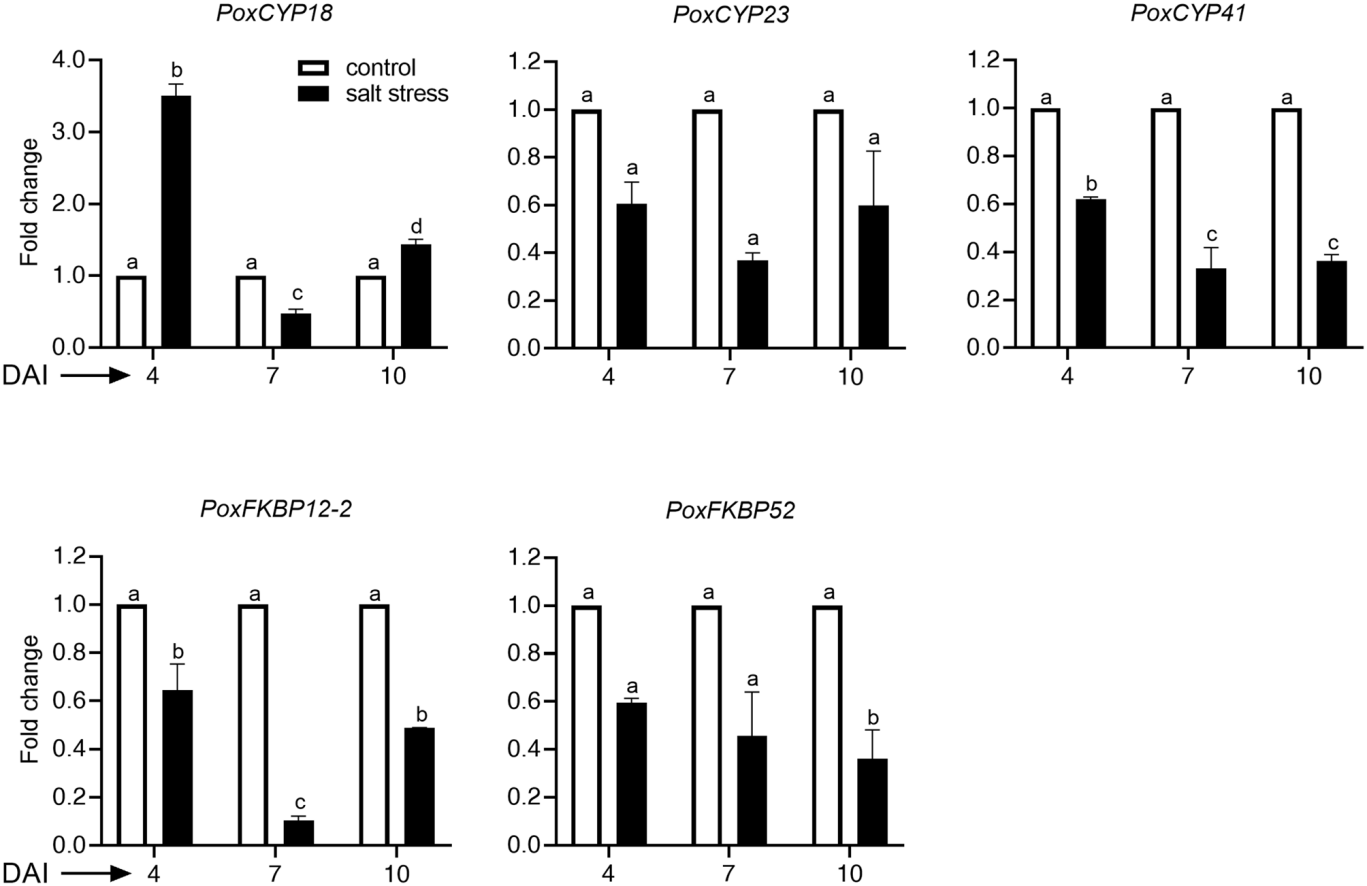
Effect of salt stress (15% NaCl) on expression of cyclophilin and FKBP genes in the mycelia of *Penicillium oxalicum.* Changes in the transcript levels were estimated by Real-time PCR analysis using *ACTIN* gene as reference. The fold change presented is with respect to the control lacking NaCl. The values depict the mean of three biological replicates ± standard error and are significant at P ≤ 0.01 (Tukey-HSD test; α = 0.05). The values with distinct letters are significantly different. (DAI: days after inoculation).

The total (nmol/s/g fresh weight mycelium) and specific (nmol/s/mg total proteins) PPIase activities under salt stress were significantly higher than control at all the three stages of growth in *P. oxalicum* (Fig. 4a–c). Further, the PPIase activity under control conditions was not regulated temporally since no significant difference in the mycelial catalytic activity was observed at different growth stages**.** On the contrary, substantial enhancement in the specific PPIase activity was noticed between 4 and 7 days after inoculation (DAI) under salt stress, that appeared to be due to the induction of PPIases since decrease in total protein content during this duration was 40.3% (from 9.04 to 5.4 mg/g fresh weight mycelium) compared to 88.7% (from 8.3 to 15.68 nmol/s/mg total proteins) increase in specific PPIase activity (Fig. 4c,d). FKBPs and cyclophilins' contribution to PPIase activity in *P. oxalicum* was evaluated by the extent of inhibition by their specific inhibitors FK506 and CsA, respectively. Whereas PPIase activity under control conditions was almost completely inhibited by CsA at all the growth stages, the CsA-induced inhibition in the presence of salt at 4 and 7 DAI was about 85% and 87% respectively, compared to almost total abrogation at 10 DAI (Fig. 4e). These observations imply that PPIase activity in the mycelia of *P. oxalicum* was predominantly contributed by the cyclophilins. However, the FK506-induced inhibition by FK506 at 4 (15%) and 7 DAI (13%) under salt stress, though statistically insignificant, was observed consistently, indicating the contribution of FKBPs to enzyme activity at these stages. We carried out real-time PCR analysis to further analyze the contribution of different PPIase genes to the mycelial PPIase activity in *P. oxalicum*. This analysis revealed that out of 18 PPIase genes, only three cyclophilin (*PoxCYP18, PoxCYP23* and *PoxCYP41*) and two FKBP genes (*PoxFKBP12-2* and *PoxFKBP52*) were expressed at all stages of growth under both control and salt stress conditions (Fig. 5). Whereas the expression of *PoxCYP18* at 4 and 10 DAI increased significantly under salt stress, the transcript levels of *PoxCYP23*, *PoxCYP41, PoxFKBP12-2* and *PoxFKBP52* at all stages of growth decreased substantially. However, the transcripts corresponding to rest of the 13 genes, including parvulins and PTPAs, could not be detected at any of the stages analysed. We carried out PCR using genomic DNA as template to further validate that the lack of amplification of rest of the 13 PPIase genes was not due to the absence of these genes in this strain. These results revealed the presence of amplicons corresponding to all the PPIase genes implying their presence in the genome (Supplementary Fig. S10).

## Discussion

The present study reports in silico characterization of PPIase gene families in *Penicillium,* and their regulation by salt tress in the mycelia of a halotolerant strain of *P. oxalicum*. This analysis revealed that the number of cyclophilins in *Penicillium* spp. varies between 7 and 11 (Table 1), which is comparable to that reported in other fungi^8,40,55,56^. The cyclophilins in *Penicillium* spp*.* were predicted to localize to different cellular compartments. The cytoplasmic cyclophilins PenCYP01 and PenCYP05, the ER-targeted PenCYP06 and the nuclear predicted PenCYP11 were observed in all *Penicillium* spp. (Table 3)*,* suggesting their indispensable role in the cell. The cyclophilins also play an essential role in mitochondria, with CPR3 and CyP-D in yeast and humans, respectively, implicated in the regulation of mitochondrial permeability transition pore^57–59^. However, contrary to the widespread prevalence of these proteins in mitochondria of different fungi^40^, our analysis revealed the presence of mitochondrial cyclophilins only in seven species of *Penicillium* (Table 1). The absence of mitochondrial cyclophilins in majority (17) of the *Penicillium* spp., therefore, needs to be validated by analyzing the CsA-inhibitable PPIase activity in these subcellular organelles.

The occurrence of different functional domains points towards the acquisition of novel roles by these cyclophilins, since domains such as TPR and WD repeats facilitate protein–protein interactions, while RRM and U-box containing proteins have been implicated in RNA stabilization and ubiquitination, respectively^60–63^. The presence of RRM and nuclear localization signal in the cyclophilins of orthogroup PenCYP08 (Table 3) suggests that these proteins may have a role in RNA processing or regulation of transcription^64,65^**.** A noteworthy feature of this study was that cyclophilins with GIT_SHD2 domain (orthogroup PenCYP12) were observed only in *P. coprophilum* and *P. griseofulvum* (Supplementary Table S2l). To our knowledge, the GIT_SHD2 domain has not been reported in cyclophilins of other fungi studied yet. Therefore, this novel combination of domains indicates species-specific neofunctionalization of cyclophilins in *Penicillium* and warrants in depth investigations to understand their cellular implications.

The FKBP repertoire in *Penicillium* varies from two in *P. decumbens* to five in *P. antarcticum* (Table 2)*,* which is consistent with similar findings in other fungi^40^. Further, the localization of *Penicillium* FKBPs to the cytosol, ER and nucleus, and the presence of a single FKBP domain in these proteins (Table 3) is also in accordance with the earlier studies^40^. The role of different FKBPs in *Penicillium* has not been analyzed yet but studies with *Schizosaccharomyces pombe* demonstrated that though not essential, the SpFKBP12 plays a vital role in the early steps of sexual development pathway^66^. Since SpFKBP12 is orthologous to PenFKBP02 members, the latter may also be performing a similar role in the cell which needs to be confirmed by further experimentation. The ER-localized FKBPs of the orthogroup PenFKBP03 (Table 3) showed a high degree of homology (66.9%) with human FKBP13 (Supplementary Table S9), a membrane-associated protein localized to the lumen of the ER^67–69^. As suggested for hFKBP13^70^, the PenFKBP03 FKBPs may also be involved in protein folding in the ER that is imperative for the survival of the cells and explains the presence of these proteins in all *Penicillium* spp. On the contrary, the ability of *P. decumbens, P. occitanis and P. steckii* to complete growth and development despite lacking either PenFKBP01 or both PenFKBP01 and PenFKBP04 members (*P. decumbens*) (Table 2) indicates redundancy in the functions of these proteins. Owing to the NPL domain (Table 3), the PenFKBP04 Group FKBPs in *Penicillium* might be involved in nucleosome assembly and regulation of gene expression, as demonstrated for the NPL containing yeast FKBP, Fpr4^71,72^, with which these proteins show considerable similarity (52.5–56.4%) (Supplementary Table S10). The existence of NPL domain suggests the acquisition of novel roles by these proteins that may be enabling the cells to respond to different developmental and environmental cues.

Parvulins have been implicated in post-phosphorylation control of diverse cellular processes such as cell division, gene expression, immune response, etc.^73–75^. The identification of parvulins in *Penicillium* with and without WW domain (Table 3) supports the earlier findings in *E. coli*, yeast and *Arabidopsis*^8,76^. Though the N-terminal WW domain in PIN1-type parvulins facilitates specific binding to phosphorylated Thr/Ser-Pro motifs and their subsequent *cis–trans* isomerization by the C-terminal PPIase domain^77^, the PIN1-type PPIases in *Arabidopsis* and *Malus domestica* plants, despite lacking this domain, exhibited no difference in substrate specificity^78^. Therefore, further studies are required to understand the significance of the WW domain in regulating the parvulin activities in *Penicillium*.

The occurrence of genes encoding PenPTPA01 and PenPTPA02 in all *Penicillium* spp., except *P. nalgiovense* and *P. steckii* that lack the PenPTPA02 proteins (Table 2), signifies their indispensable role in the cell. These proteins may be involved in the activation of PP2A-like phosphatases, as reported for their yeast orthologues YPA1 and YPA2^6^. Though information about the active site residues required for PPIase activity in PTPAs is elusive, deletion of a conserved domain 208-GVWGLD-213 in YPA1 resulted in about 400-fold attenuation of phosphotyrosyl phosphatase activation reaction of PP2A^6^. Since the same amino acid stretch is also present in the PenPTPA01 and PenPTPA02 members in all *Penicillium* spp. (Supplementary Fig. 11), these proteins may likely have PPIase activity. Cloning and characterization of these proteins are, nevertheless, required to validate this speculation.

### Analysis of PPIase activity and expression of PPIase genes

Biochemical investigations revealed significantly higher PPIase activity under salt stress in the halotolerant *P. oxalicum*, which was predominantly inhibited by CsA, underscoring the contribution of cyclophilins (Fig. 4a–e). It is likely that the stress induced PPIase activity may be conferring protection against salt induced damage, as reported for other PPIases^7,25,26^. Of the three cyclophilins (*PoxCYP18, PoxCYP23* and *PoxCYP41*) and two FKBP genes (*PoxFKBP12-2* and *PoxFKBP52*) expressed in the mycelia, the salt-induced increase was observed only for *PoxCYP18* (Fig. 5), implying its contribution to the mycelial PPIase activity. Relative to 7 DAI, significantly higher expression of *PoxCYP18* at 4 and 10 DAI under salt stress implies biphasic or multiphasic regulation by stress which was also reported earier for a NAK group plant protein kinase, F8A24.12, in Arabidopsis^79^. Though the maximum increase in transcript levels of *PoxCYP18* was observed at 4 DAI, the total PPIase activity was higher at 10 DAI. It is likely that this gene may be regulated at the post-transcriptional level and/or the expressed protein is highly stable and gets accumulated with time, thereby, leading to enhanced activity at later stages of growth. Estimation of the PPIase proteins by immunoblotting is, nonetheless, required to understand the molecular basis of this observation. Since protection by cyclophilins and FKBPs against stress-induced damage to the cell is attributed to their PPIase activity and/or chaperone functions^80^, further biochemical characterization of these proteins in *Penicillium* is imperative to elucidate their precise mechanism of action. Although expression of other PPIases was not observed at the stages studied, their role in specific developmental processes and adaptation to other stresses cannot be ruled out as previous studies have demonstrated the role of these genes in the regulation of several developmental process^80^. Further, the possibility that some of these genes are pseudogenes can also not be dismissed. Therefore, detailed studies on the expression of PPIase genes at different developmental stages are required to enhance our understanding of the role of these genes in halotolerance of *P. oxalicum*.

## Conclusions

To conclude, this study has shown that the number of genes encoding PPIases varies between 7–11, 2–5, 1–2 and 1–2 for cyclophilins, FKBPs, parvulins and PTPAs, respectively, in different species of *Penicillium*. Though cyclophilins, FKBPs and parvulins have been characterized earlier in several fungal species, this is the first study to characterize all the PPIases in *Penicillium* spp. Despite conservation of the secondary structure of the CLD and FKBP domains, the cyclophilins and FKBPs in *Penicillium* spp. have undergone divergence by the acquisition of novel domains such as PP2C and GIT_SDH, implying neofunctionalization. In addition to the cytoplasm, the localization of PPIases in *Penicillium* to other subcellular compartments viz., ER, mitochondria and nucleus, suggests their specific roles. This study further demonstrated that the mycelial PPIase activity in a halotolerant strain of *P. oxalicum* is induced significantly under salt stress and is primarily contributed by the cyclophilins, signifying the role of these genes in stress response.

## Materials and methods

The halotolerant fungal strain HP1 used in this study was isolated from the leaves of healthy plants of C*itrus limon* and identified on molecular basis by the Microbial Type Culture Collection (MTCC) Chandigarh, India. The culture was also characterized microscopically. The slide culturing technique was performed to determine the microscopic characters of the fungus and its morphological characterization according to the standard taxonomic key characters. Identification of the culture on the molecular basis was carried out by amplification of the internal transcribed spacer (ITS) region containing 5.8S rRNA gene that yielded an amplicon of 338 bp. Sequencing of the amplicon and its subsequent analysis by basic local alignment search tool (BLAST) on the National Center for Biotechnology Information (NCBI) server revealed its maximum similarity with *P. oxalicum* (NR_121232.1) (Supplementary Fig. S12)*.*

The mycelial production was carried out by inoculating one plug (8 mm diameter) of actively growing fungal culture in 250 ml Erlenmeyer flasks containing 50 ml Sabouraud production medium that either lacked or contained NaCl (15%). The cultures at 4, 7 and 10 DAI were taken for analysis since the three stages represent the early exponential, late exponential and stationary phase of growth, respectively. The *P. oxalicum* cells did not lose their viability after 10 days of culture in the medium containing 15% NaCl since they resumed growth when again transferred to the unstressed medium. The inoculated flasks were incubated at 30ºC for 10 days on a rotary shaker at 180 rpm and withdrawn at different time intervals for analyses. For dry weight estimations, the cultures (50 ml) were filtered through Millipore membrane filters (0.45 mm) under vacuum and dried at 80 °C until constant weight. The growth curves were constructed from the results obtained from two independent experiments, each carried out in triplicate, and were used to determine the final fungal biomass yield. The effect of salt on colony diameter was analyzed by inoculating Sabouraud agar plates containing 15% NaCl with fungal culture, followed by incubation at 30ºC. The fungal growth was monitored daily for ten days by measuring the diameters of individual colonies.

### Estimation of peptidyl-prolyl *cis–trans* isomerase (PPIase) activity

The total soluble proteins were extracted from the filtered mycelia of *P. oxalicum* by adding lysis buffer [50 mm Tris–Cl, 150 mm NaCl, 1 mm EDTA (pH 8), 10% Glycerol, 1 mm phenylmethylsulfonyl fluoride] followed by sonication with 10 s on and 5 s off for 5 min at 4 °C. The homogenate was centrifuged at 6500 rpm for 30 min at 4 °C and the supernatant was collected and stored at – 20 °C after filtering through 0.45 um filters. The total soluble proteins were estimated according to Bradford’s method^81^ using bovine serum albumin as the standard. The PPIase activity in the crude extracts was estimated by a chymotrypsin-based coupled reaction at 15 °C for 360 s^82^. The 1 ml assay mixture contained 80 µM succinyl-ala-ala-pro-phe-p-nitroanilidine as test peptide, assay buffer [50 mM HEPES (pH 8.0), 150 mM NaCl, 0.05% Triton X-100] and different concentrations of the crude protein. The reaction was initiated by addition of chymotrypsin at a final concentration of 300 µg/ml. The absorbance change at 390 nm was monitored at 15 °C by using Spectrophotometer (Perkin-Elmer Lambda Bio 25) equipped with Peltier temperature control system. The cyclophilin- and FKBP-associated PPIase activities were determined by the extent of inhibition of reaction in the presence of the specific inhibitors CsA and FK506, respectively. The inhibitors were added to the assay mix 30 min before starting the reaction and incubated at 4 °C. The PPIase activity was calculated as the product of the difference in the catalysed and uncatalysed first-order rate constants (derived from the kinetics of the absorbance change at 390 nm) and the amount of substrate in each reaction.

### Bioinformatics analysis

The amino acid sequences of cyclophilins, FKBPs, parvulins and PTPAs of 24 different *Penicillium* spp. were retrieved from the NCBI server (http://www.ncbi.nlm.nih.gov/) on the basis of the results obtained with a series of iterative BLASTP homology searches using a variety of PPIases viz*.,* cyclophilin A (hCYPA), FKBP12 (hFKBP12) and parvulins (hPAR14/hPIN1), and *S. cerevisiae* PTPAs (YPA1/YPA2) as queries. The parameters of each BLASTP run were kept as default (for example E-value: 0.05; word size: 6; Matrix: BLOSUM62) to make it even more specific. The domains in these putative PPIase proteins were identified with CDD (https://www.ncbi.nlm.nih.gov/Structure/cdd/wrpsb.cgi), PFAM (http://pfam.xfam.org), Prosite (https://prosite.expasy.org/) and SMART (http://smart.embl-heidelberg.de) database servers using default search parameters. Based on the consensus of information obtained from these servers, the protein sequences harboring the PPIase domain were kept for subsequent analyses.

Subcellular localization were predicted with LocTree3 (https://rostlab.org/services/loctree3/), while the presence of Nuclear Localization Signals, Signal peptides and transmembrane domains was further confirmed with NLS mapper (http://nls-mapper.iab.keio.ac.jp/cgi-bin/NLS Mapper_form.cgi), SignalP4.0 (http://www.cbs.dtu.dk/services/SignalP-4.0/) and TMHMM ServerNv2.0 (http://www.cbs.dtu.dk/services/TMHMM/) servers, respectively using their default setups.

The predicted molecular weights and the isoelectric points were determined by the compute_pi server (http://web.expasy.org/computepi/) while the pairwise percentage sequence identity and similarity were calculated using the Matrix Global Alignment Tool (MatGAT) version 2.02, selecting BLOSUM50 as scoring matrix.

Multiple sequence alignments of amino acid sequences belonging to cyclophilins, FKBPs and parvulins were performed using MUSCLE algorithm in Jalview (http://www.jalview.org/) standalone package version 2.11.1.3 with default parameters. The Jpred3 server (http://www.compbio.dundee.ac.uk/www-jpred/) integrated in Jalview was used to predict secondary structure elements. This alignment setup in Jalview was specifically used to generate a graphical view of the multiple sequence alignment highlighting the active site residues as well as the secondary structure elements.

The phylogenetic tree was constructed on the Mega X platform by generating a multiple sequence alignment using the built in MUSCLE algorithm and then employing neighbor-joining (NJ) method with bootstrap value of 1000. The tree files thus obtained were visualized and annotated using Iterative Tree of Life (iTOL; https://itol.embl.de/itol.cgi). The MEME web server (http://meme-suite.org/tools/meme) was used to analyze conserved and potential motifs with the parameter settings for a minimum motif width, a maximum motif width and maximum number of motifs as 6, 150, and 15, respectively. Exon–intron organization was predicted using the Gene Structure Display Server 2.0 (GSDS 2.0) (http://gsds.cbi.pku.edu.cn/) by comparing the coding sequences with corresponding DNA sequences.

### cDNA synthesis and quantitative Real-time PCR (qRT-PCR)

The expression of different cyclophilin, FKBP, parvulin and PTPA genes in *P. oxalicum* was analyzed by the real-time PCR by designing gene-specific primers (Supplementary Table S11). Total RNA from the harvested mycelia was isolated at different stages of growth using a Trizol reagent (Invitrogen, USA) according to the manufacturer's instructions. After removing DNA by DNaseI (Sigma-Aldrich) treatment, the RNA was quantified, and its integrity was confirmed by denaturing agarose gel electrophoresis (1.4%) followed by staining with ethidium bromide. Superscript III First-strand synthesis system kit (Invitrogen) was used to synthesize cDNA from 5 μg RNA using Random Hexamer primers according to the manufacturer's instructions. The cDNA products were diluted tenfold prior to use for real-time PCR. The primers for qRT-PCR were designed using Primer-BLAST^83^ and primer 3 (https://bioinfo.ut.ee/primer3-0.4.0/). Semi-quantitative reverse transcriptase (RT)-PCR was performed to check the specificity of primers corresponding to all the 18 different PPIase genes of *P. oxalicum* prior to the qRT-PCR. The qRT-PCR was carried out using AriaMx Real-time PCR system with Brillant III ultra-Fast SYBR green QPCR master mix (Agilent Technologies, USA) according to manufacturer's protocol. The 10 μl qRT-PCR reaction consisted of 1x SYBR Green QPCR master mix, 60 ng of cDNA and 100 nM forward and reverse primers. The PCR programme comprised of initial incubation at 95 °C for 10 min, followed by 40 cycles of denaturation at 95 °C for 10 s; annealing for 15 s at 60 °C and 20 s extension at 72 °C, followed by melt curve analysis to verify the amplification specificity. To check the contamination, dimer formation and presence of genomic DNA, no template and No reverse transcriptase controls were also included. Gene encoding actin (ACTIN) was used as a reference. The Ct values were processed by 2^−ΔΔCt^ method to calculate the relative mRNA levels for different genes^84^. All analyses were performed in three biological replicates with three technical replicates. The data obtained were subjected to ANOVA. Conventional PCR with genomic DNA^85^ of *P. oxalicum* as template (100 ng) was carried out in a 20 μl reaction volume that contained 0.4 μM gene-specific forward and reverse primers (Supplementary Table S11), 1 U *Taq* polymerase, 1x standard *Taq* reaction buffer, and 0.2 mM of each dNTP under the following conditions: 95 °C for 5 min, followed by 35 cycles of 95 °C for 30 s, 60 °C for 30 s, 68 °C for 1 min, and a final extension at 68 °C for 7 min.

### Statistical analysis

All the data were presented as mean ± S.E. All the experiments were performed in triplicate unless otherwise specified. The data were analyzed by two-way analysis of variance (ANOVA) via Tukey's multiple comparison test using Graph pad prism 7 software.

## Supplementary Information

Supplementary Information
