# Supplementary material for: Genome-wide characterization of peptidyl-prolyl cis–trans isomerases in Penicillium and their regulation by salt stress in a halotolerant P. oxalicum
Source: Sci Rep. 2021 Jun 10;11:12292. doi: 10.1038/s41598-021-91602-8 (PMC8192932; doi:10.1038/s41598-021-91602-8)
Supplement: Supplementary file 1 — Supplementary Information [file 41598_2021_91602_MOESM1_ESM.pdf]

Title: Genome-wide characterization of peptidyl-prolyl *cis-trans* isomerases in *Penicillium* and their regulation by salt stress in a halotolerant *P. oxalicum*

Authors name:

Mangaljeet Singh[1], Kirandeep Kaur[1#], Avinash Sharma[2#], Rajvir Kaur[2$], Dimple Joshi[1$], Megha Chatterjee[1$], Iman Dandapath[1$], Amarjeet Kaur[2], Harpreet Singh[3], Prabhjeet Singh[1*]

[1] Department of Biotechnology, Guru Nanak Dev University, Amritsar, Punjab, India, 143005

[2] Department of Microbiology, Guru Nanak Dev University, Amritsar, Punjab, India, 143005

[3]Department of Bioinformatics, Hans Raj Mahila Maha Vidyalaya, Jalandhar, Punjab, India 144008

# Equal Contribution, $ Equal Contribution, *****Corresponding author

Email: Mangaljeet Singh- mangaljeetsingh91@gmail.com, Kirandeep Kaur- kiran.91nsr@gmail.com, Avinash Sharma- sharma91avinash@gmail.com, Rajvir Kaur- rajvir2204@gmail.com, Dimple Joshi- dimplejoshi279@gmail.com, Megha Chatterjee- meghac.321@gmail.com, Iman Dandapath- dandapath.iman1196@gmail.com, Amarjeet Kaur -amarjeet_b@rediffmail.com, Harpreet Singh- harpreetsingh05@gmail.com, Prabhjeet Singh- singhprabhjeet62@gmail.com

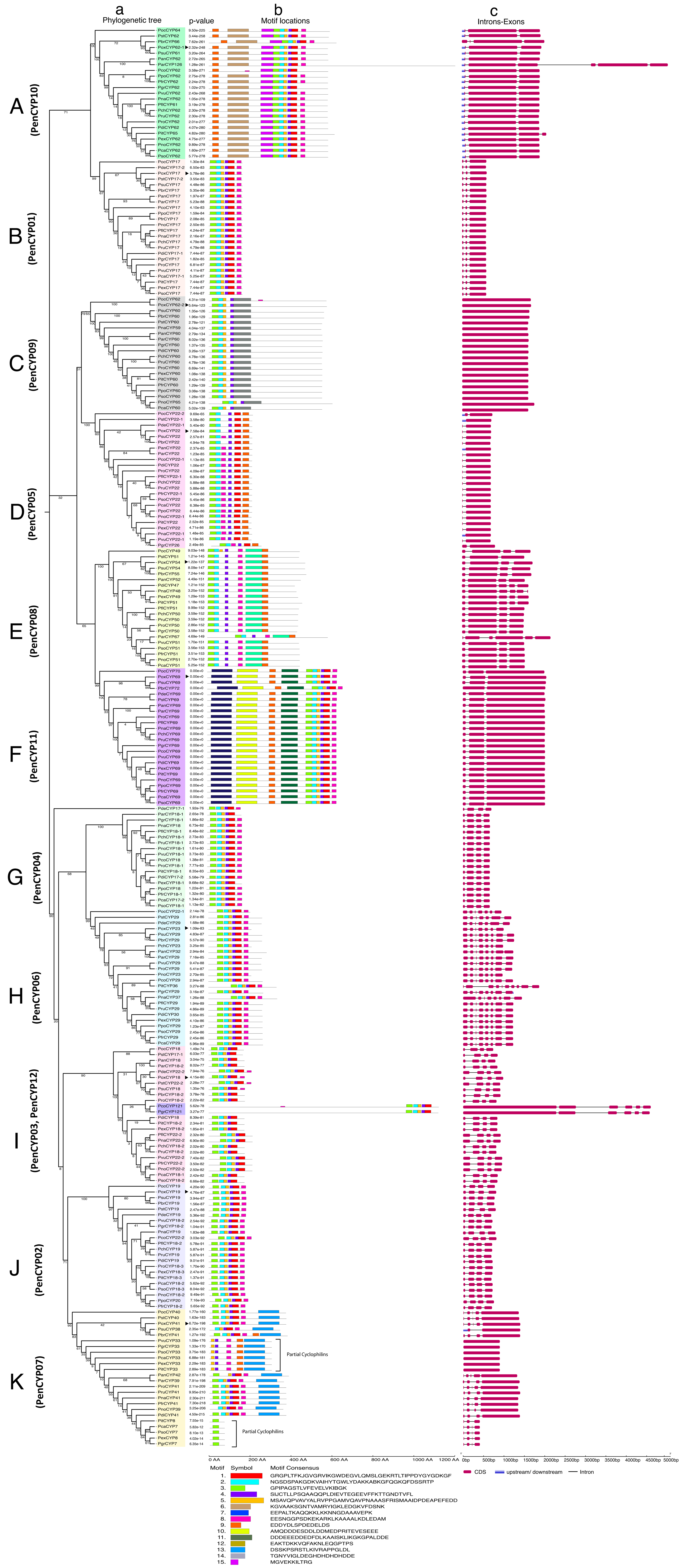

Supplementary Figure S1: Phylogenetic tree, encoded amino acid motifs and intron-exon structure of cyclophilin genes. (a) The phylogenetic tree of 248 cyclophilins was constructed using the MEGA X (v10.1.7) software (http://www.megasoftware.net) with the NJ method. The constructed tree was further annotated with the Iterative Tree of Life (http://itol.embl.de/). Percentages of bootstrap scores are indicated below the branches. Black triangles represent the cyclophilins of *Penicillium oxalicum*.
(b) The different motifs were identified through MEME analysis (http://meme-suite.org/). The different-colored boxes, named at the bottom, with their consensus sequence represent the conserved motifs. Black lines represent the non-conserved sequences.
(c) The corresponding exon-intron structures of cyclophilin genes were determined by the Gene Structure Display Server (http://gsds.cbi.pku.edu.cn/). The brownish red boxes indicate exons and single lines represent introns. The untranslated regions are indicated by blue boxes. The sizes of exons and introns can be estimated using the scale at the bottom. The figures a, b and c were redrawn by using Adobe Illustrator (v25.2.3) (https://adobe.com/products/illustrator).

Supplementary Figure S2: Multiple sequence alignment analysis of human cyclophilin A (hCYPA) and cyclophilin-like domains (CLDs) of cyclophilins of different orthogroups by using MUSCLE algorithm in Jalview software (v2.11.1.3) (http://www.jalview.org/). Secondary structure of hCYPA (PDB Id: 6i42) was aligned with *Penicillium* cyclophilins. Green arrows represent beta-sheets, and red objects signify the alpha helix. Residues believed to be important for PPIase activity and cyclosporin A interaction are highlighted in black. The substitutions with respect to hCYPA active site residues are highlighted in pink.

Orthogroup PenCYP01

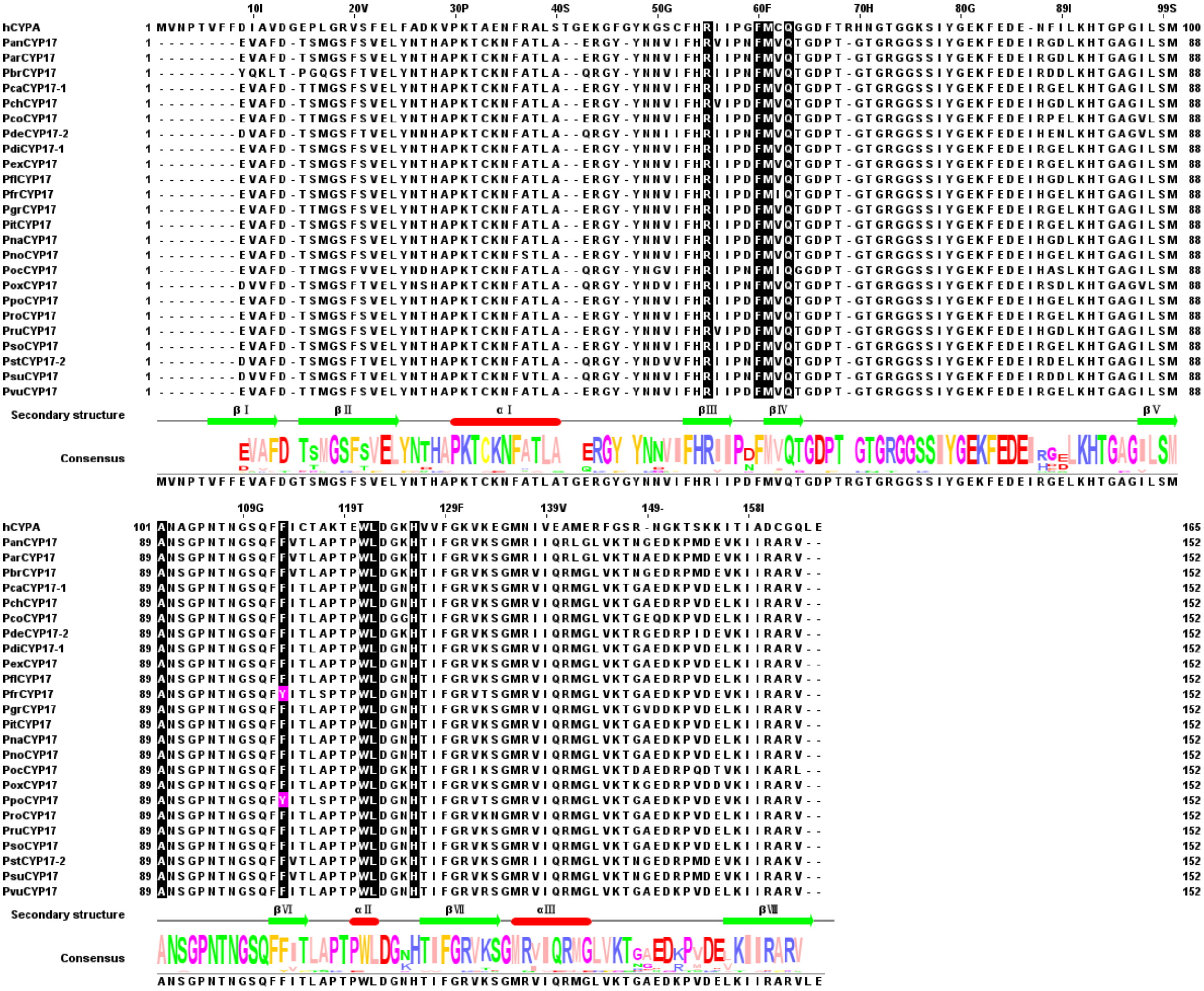

Continue on next page

Orthogroup PenCYP02

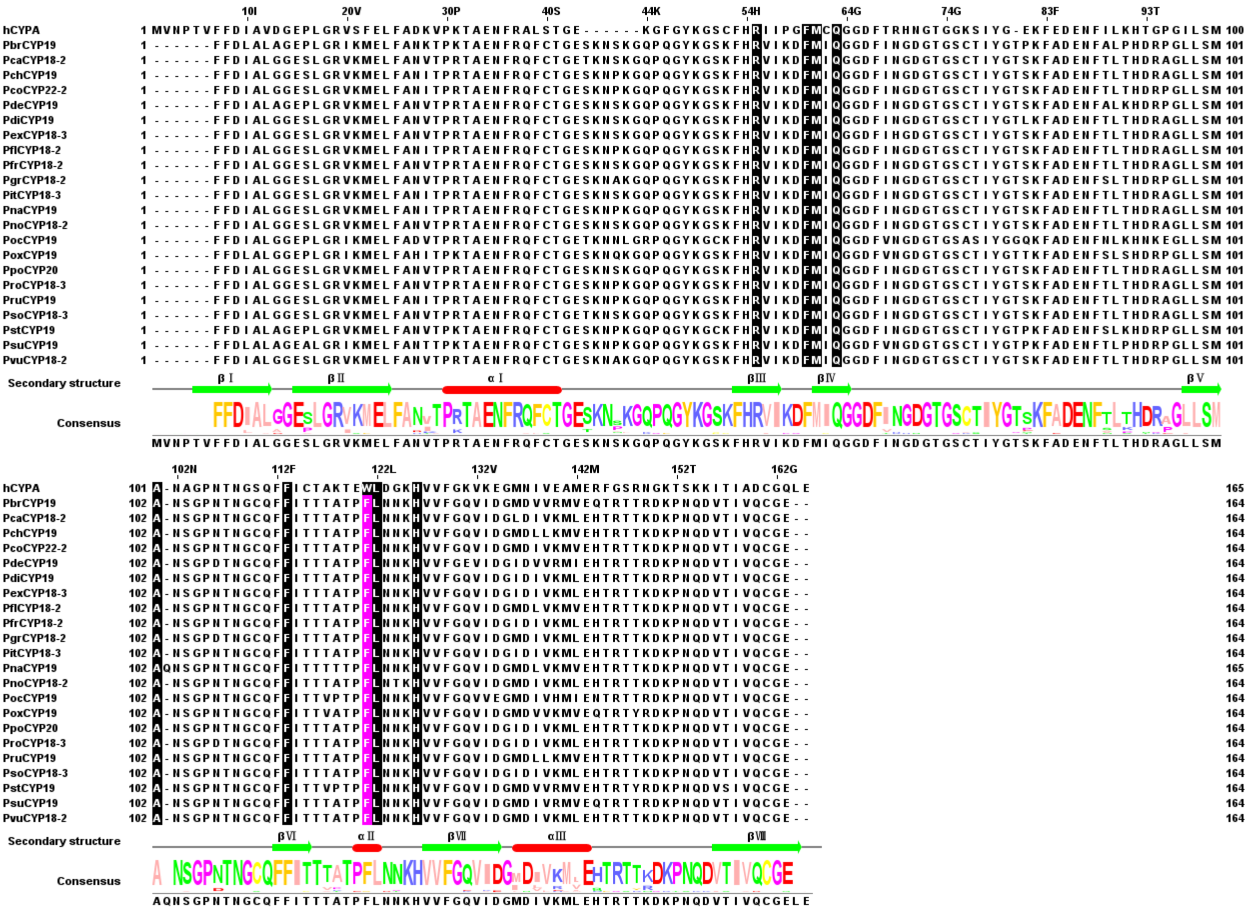

Continue on next page

Orthogroup PenCYP03

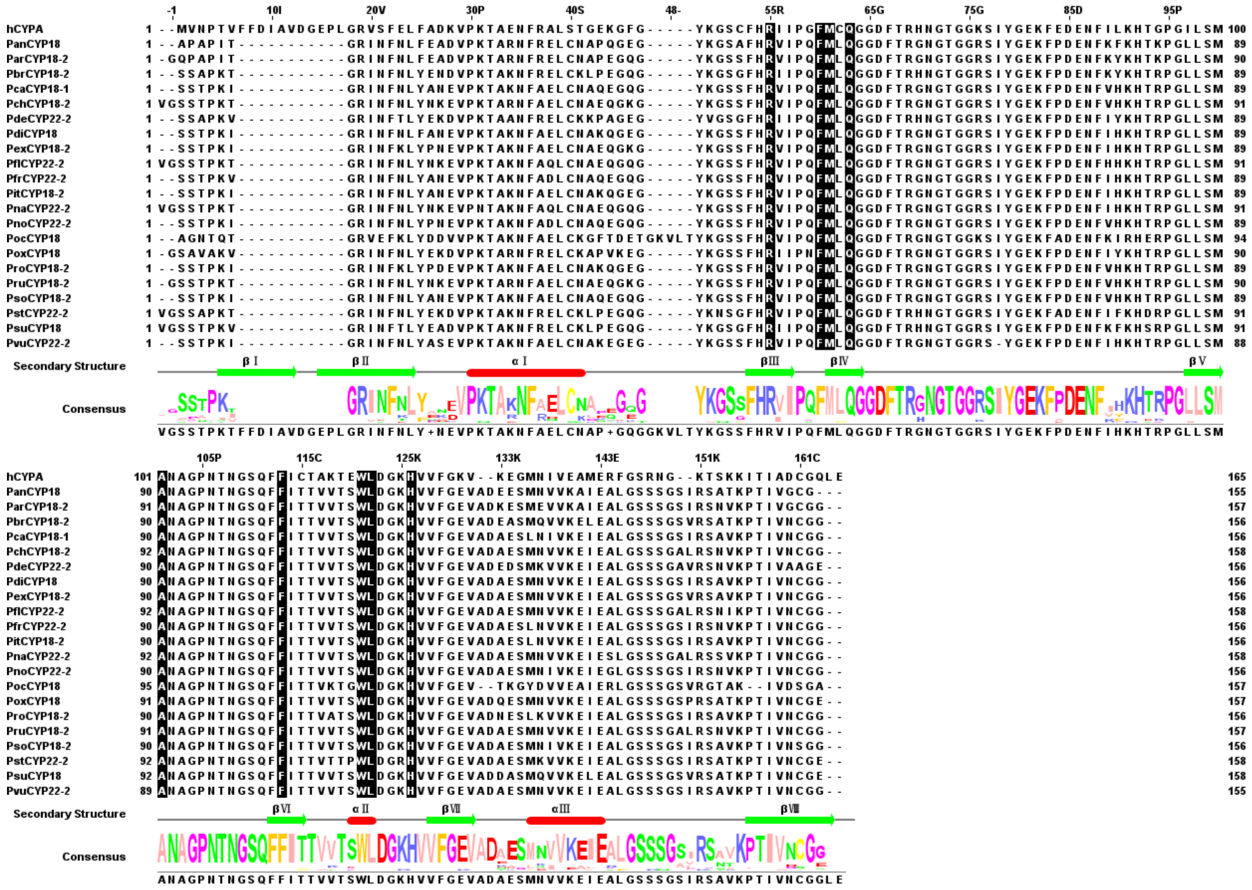

Continue on next page

Orthogroup PenCYP04

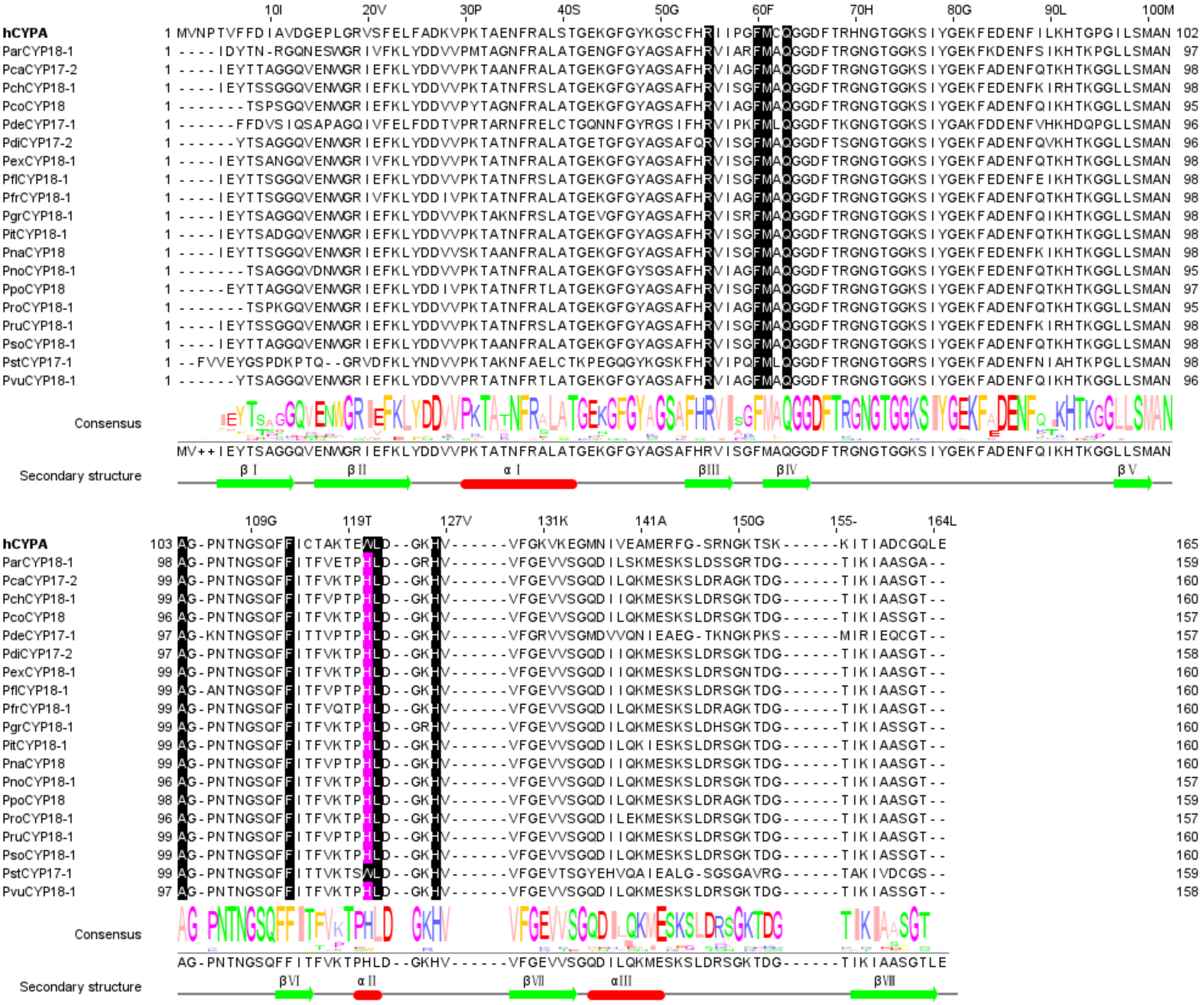

Continue on next page

Orthogroup PenCYP05

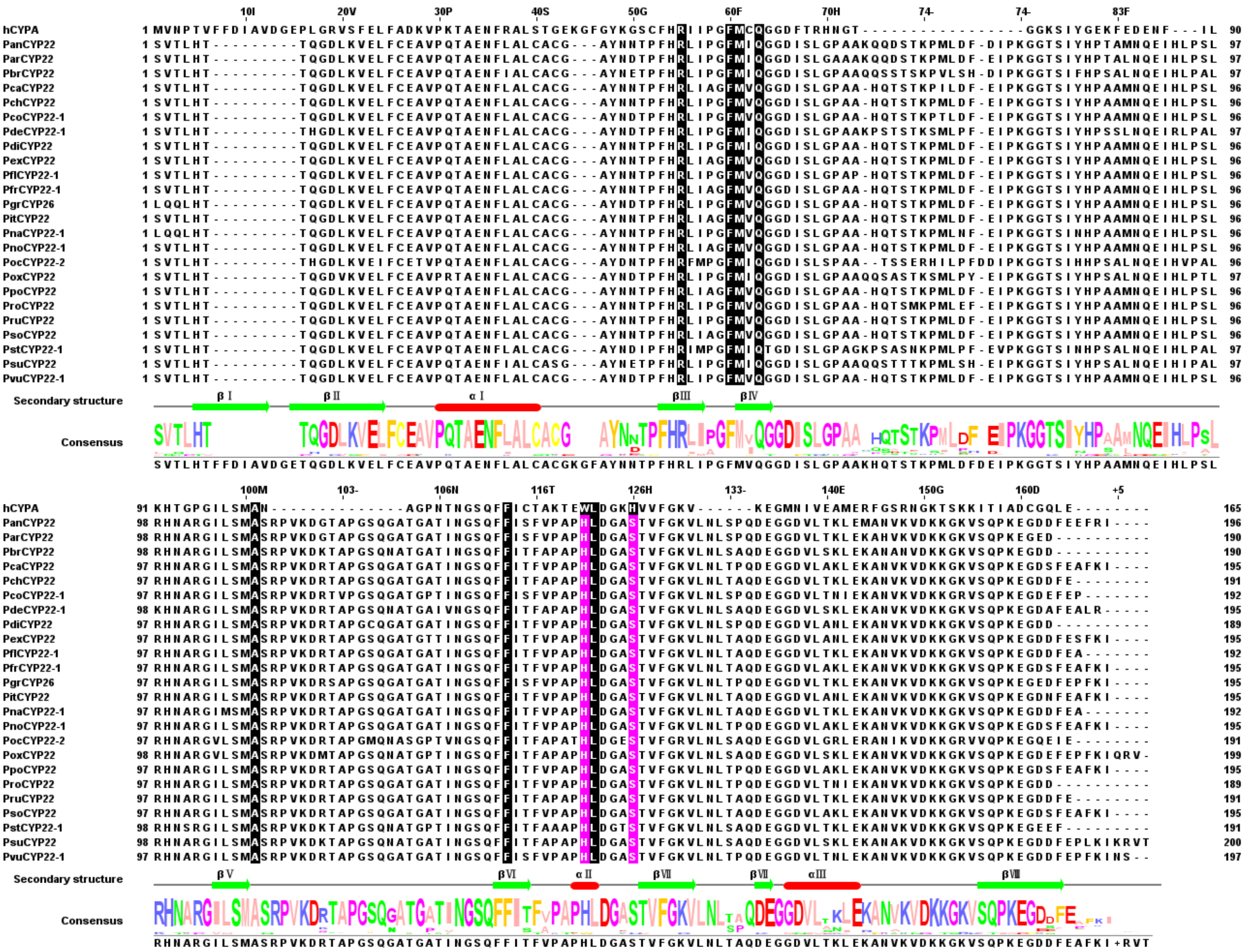

Continue on next page

Orthogroup PenCYP06

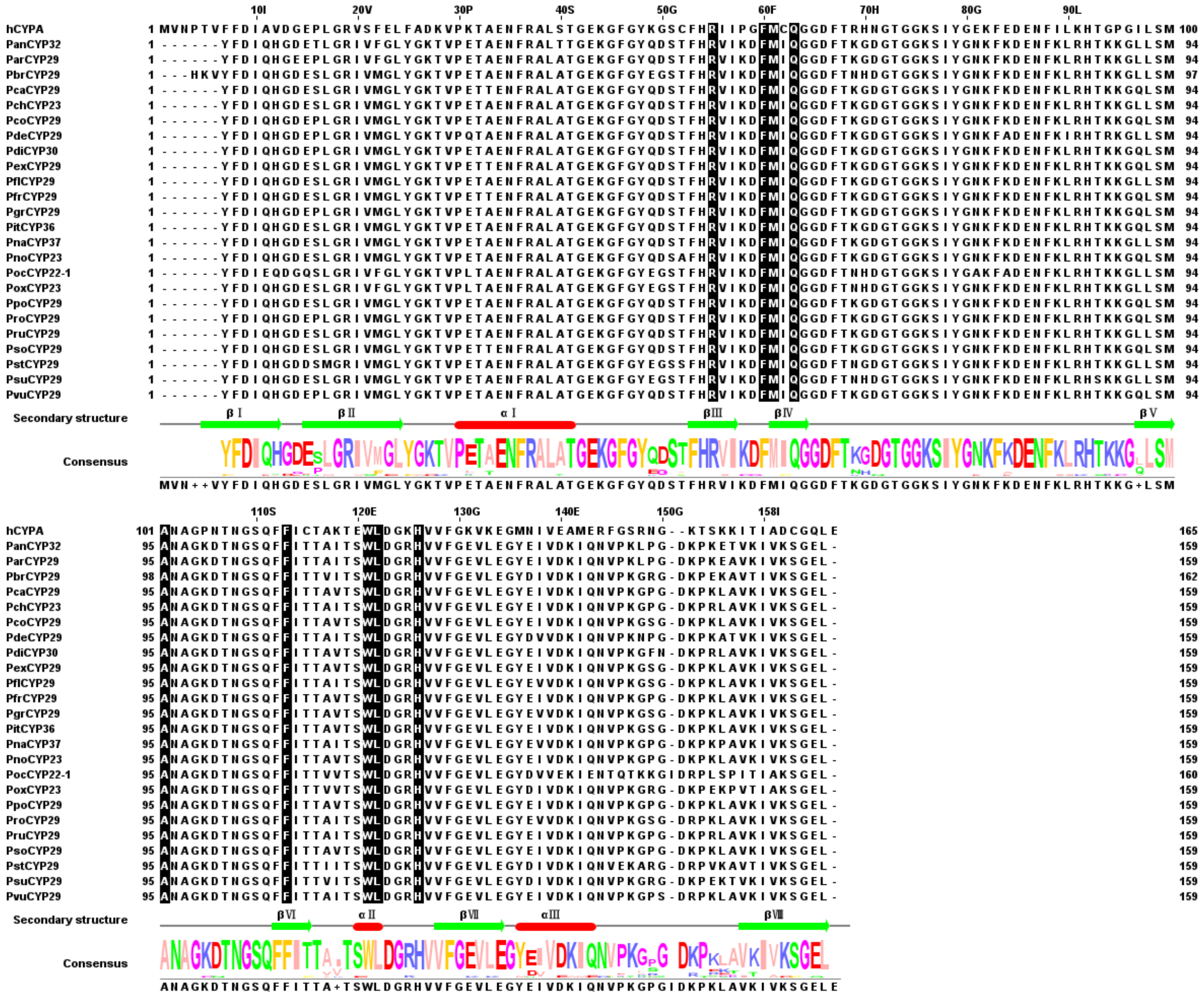

Continue on next page

Orthogroup PenCYP07

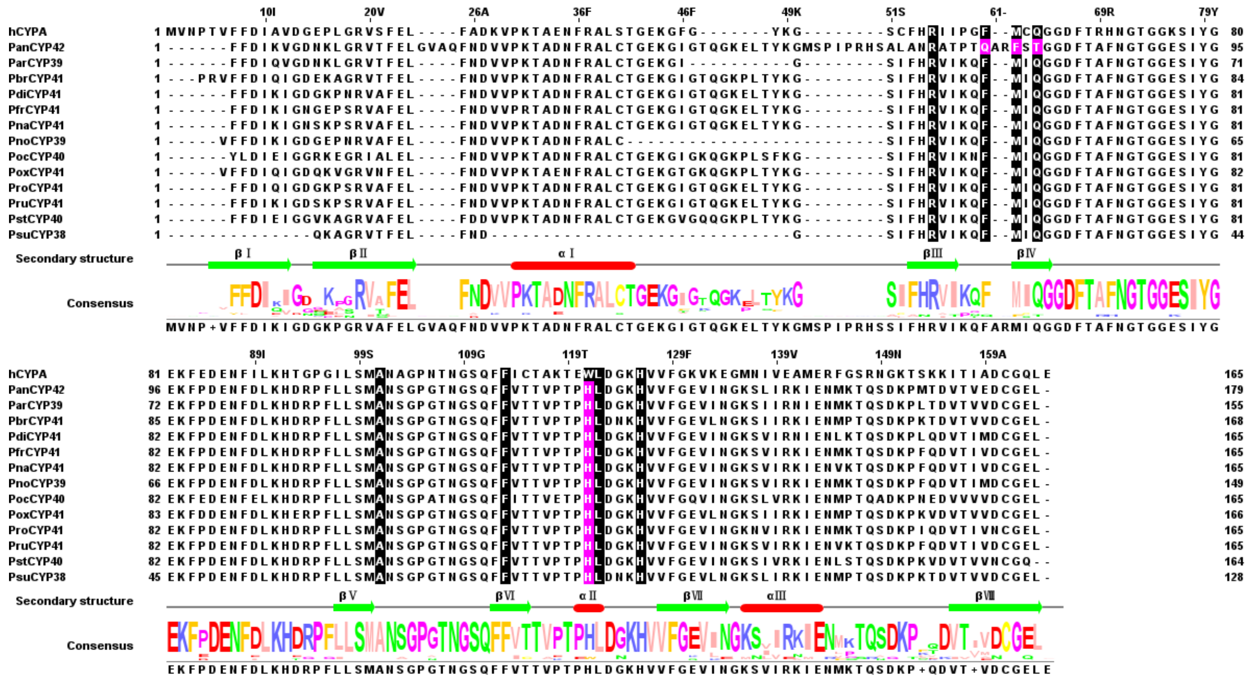

Continue on next page

Orthogroup PenCYP08

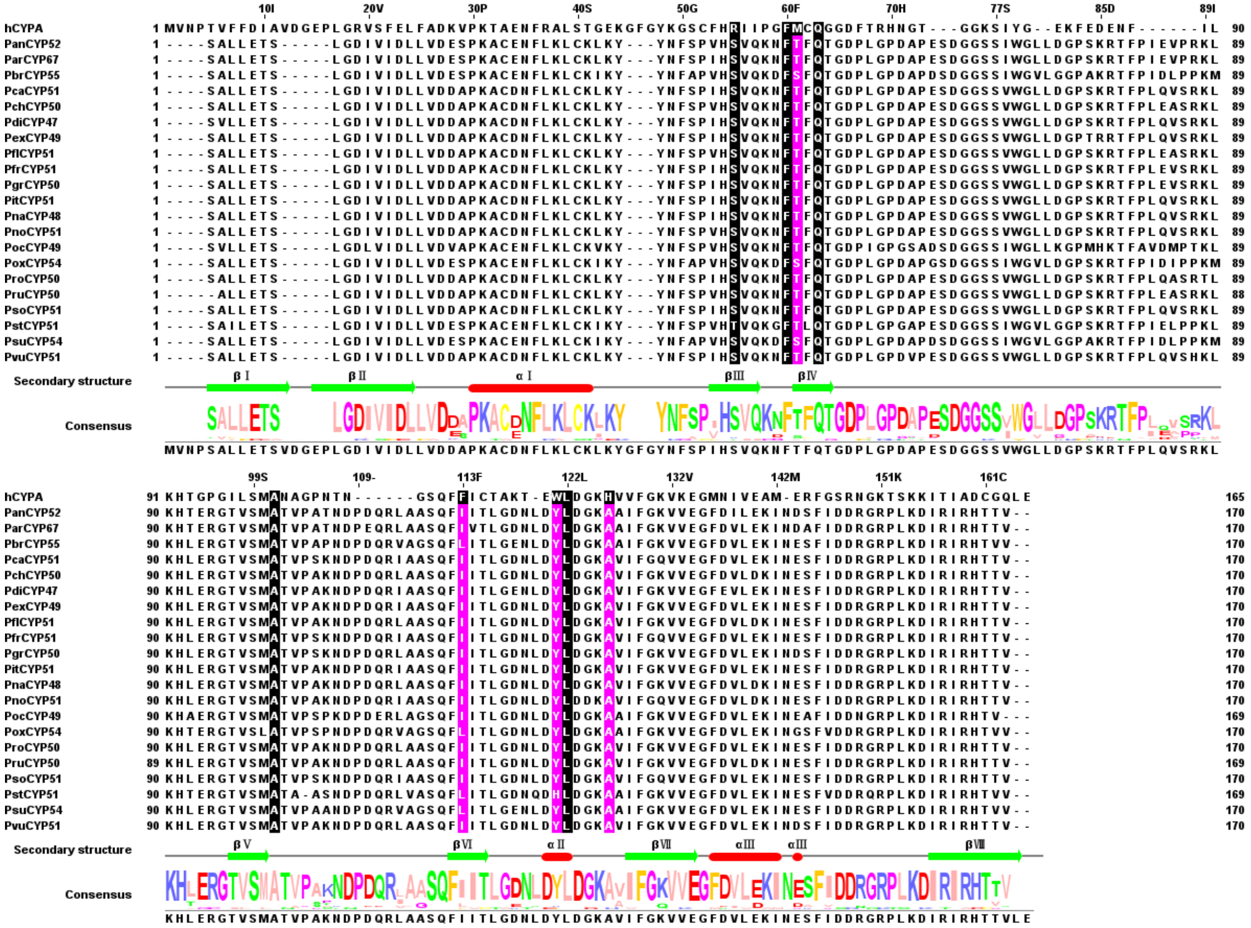

Continue on next page

Orthogroup PenCYP09

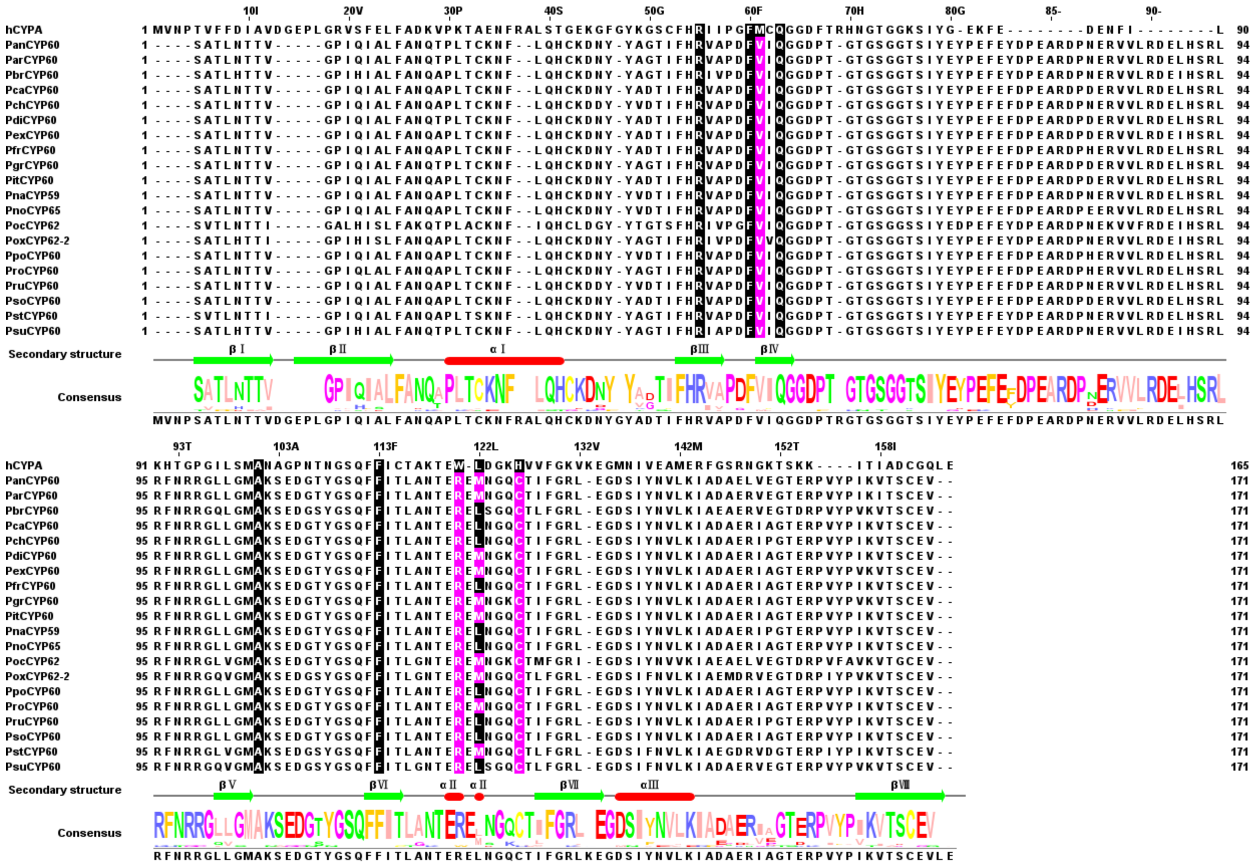

Continue on next page

Orthogroup PenCYP10

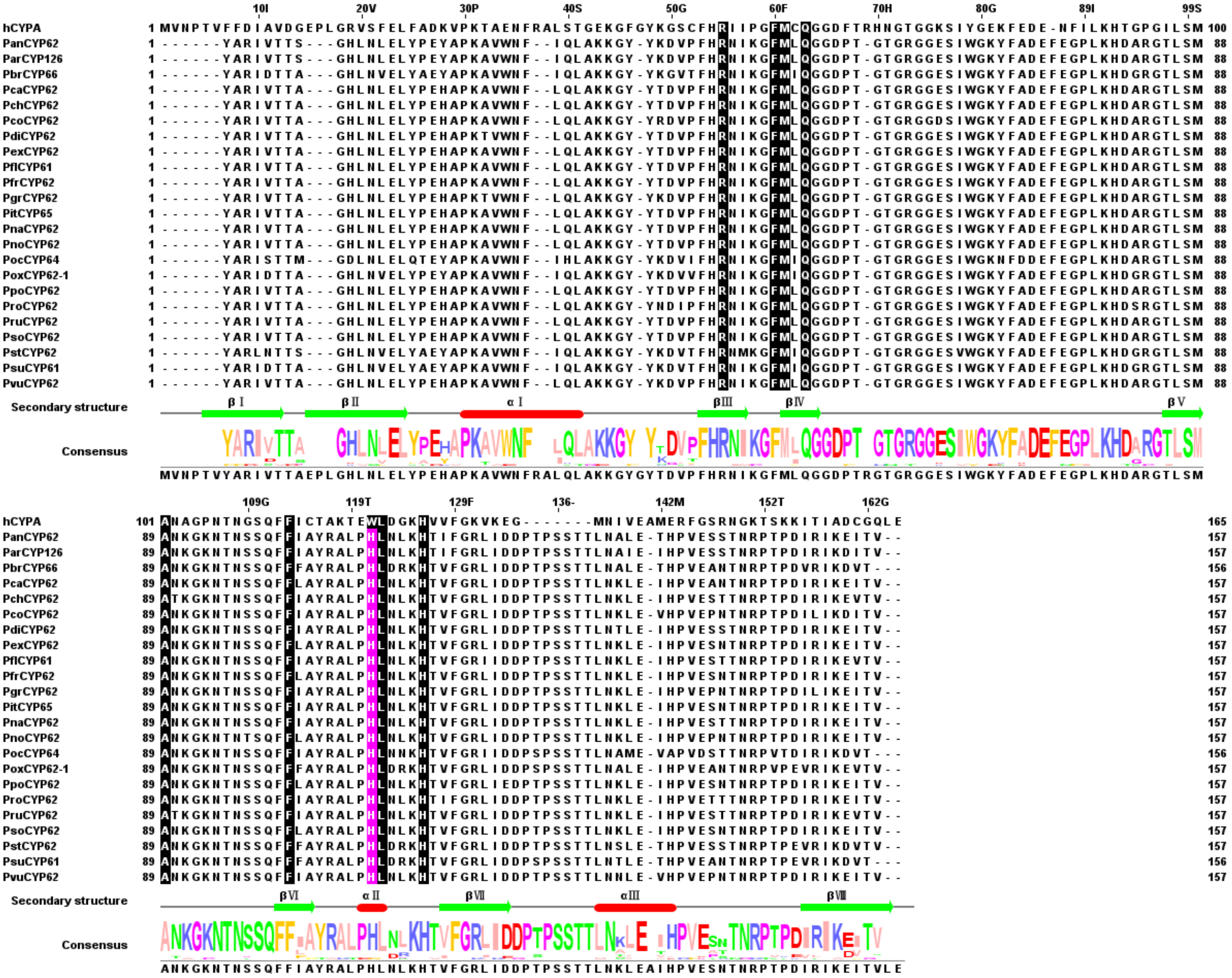

Continue on next page

Orthogroup PenCYP11

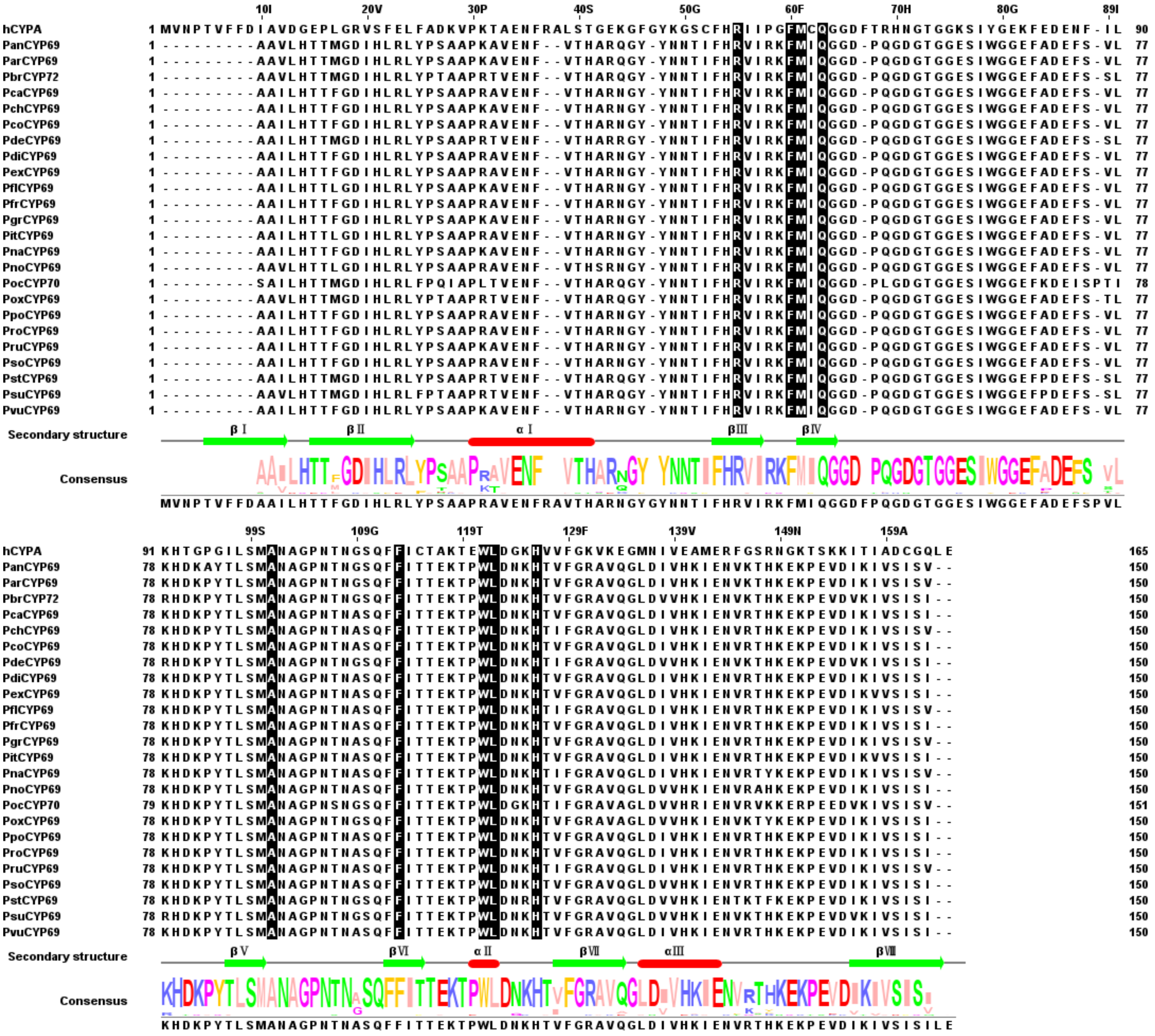

Continue on next page

Orthogroup PenCYP12

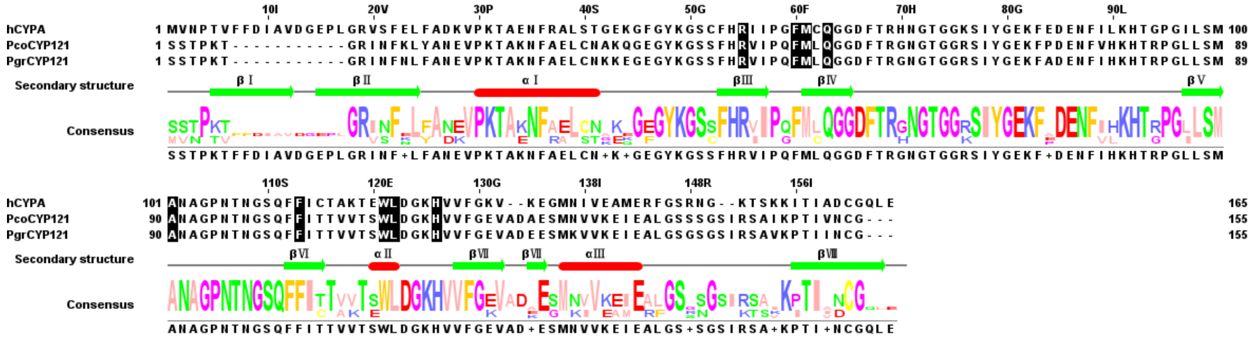

End of supplementary figure S2

Supplementary Figure S3: Multiple sequence alignment of PcaCYP7, PexCYP8, PgrCYP7, PitCYP8 and PsoCYP7 with PcaCYP33, PexCYP33, PgrCYP33, PitCYP33 and PsoCYP33, respectively, corresponded to full-length sequence of members (ProCYP41 and PruCYP40) of the same orthogroup (PenCYP07). MUSCLE algorithm in Jalview software (v2.11.1.3) (http://www.jalview.org/) was employed for this analysis.

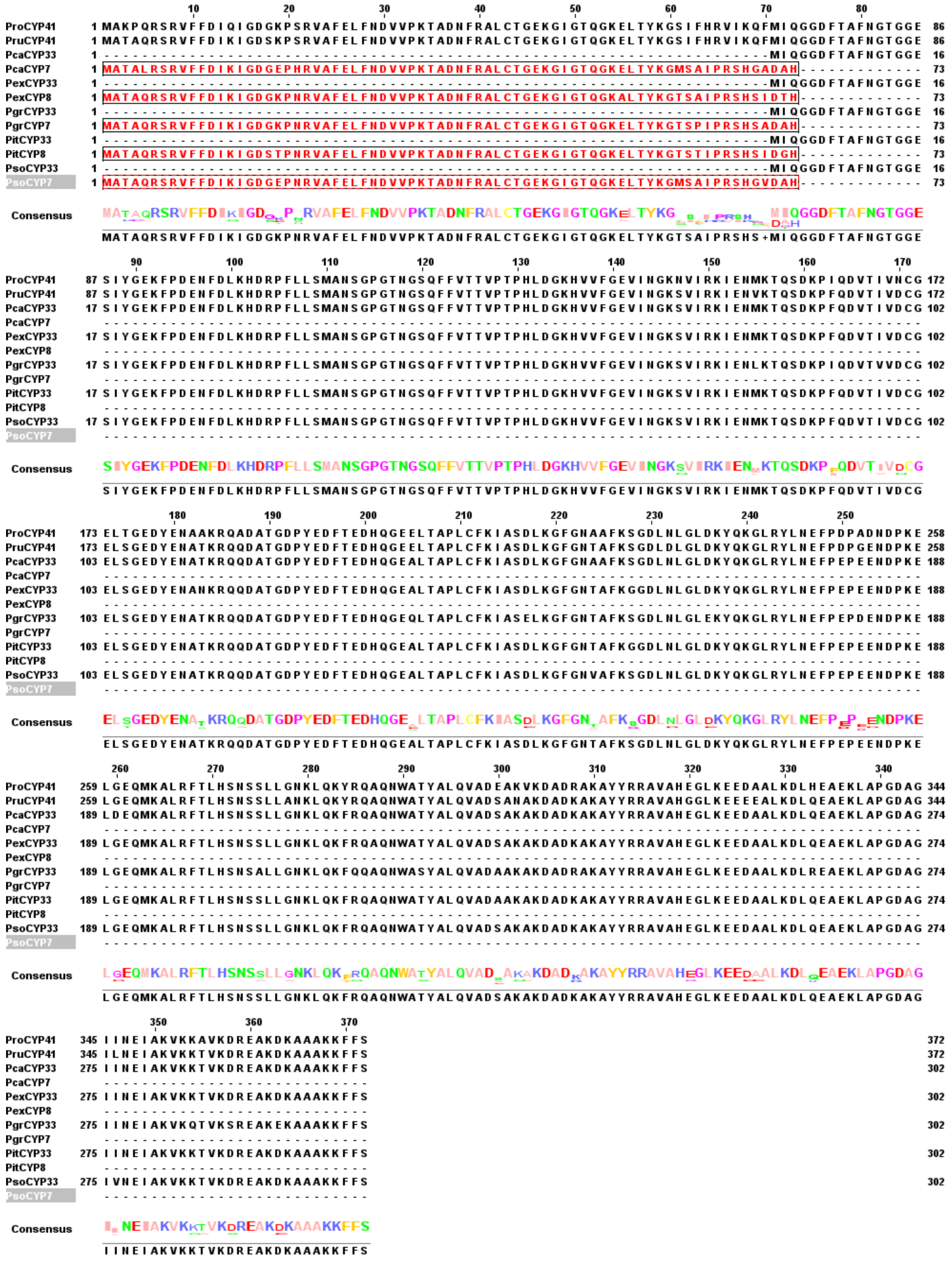

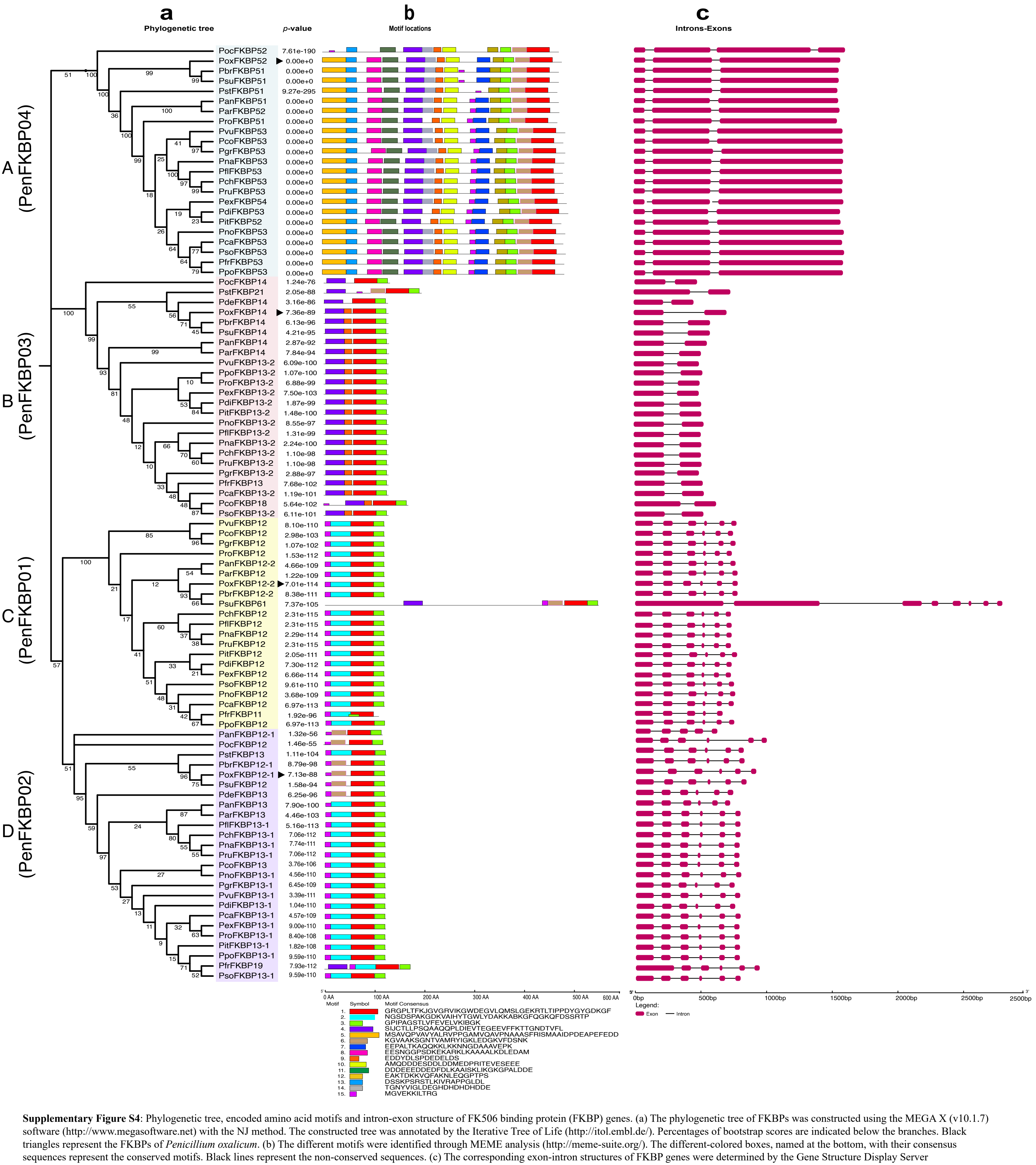

Supplementary Figure S4: Phylogenetic tree, encoded amino acid motifs and intron-exon structure of FK506 binding protein (FKBP) genes. (a) The phylogenetic tree of FKBPs was constructed using the MEGA X (v10.1.7) software (http://www.megasoftware.net) with the NJ method. The constructed tree was annotated by the Iterative Tree of Life (http://itol.embl.de/). Percentages of bootstrap scores are indicated below the branches. Black triangles represent the FKBPs of *Penicillium oxalicum*. (b) The different motifs were identified through MEME analysis (http://meme-suite.org/). The different-colored boxes, named at the bottom, with their consensus sequences represent the conserved motifs. Black lines represent the non-conserved sequences. (c) The corresponding exon-intron structures of FKBP genes were determined by the Gene Structure Display Server (http://gsds.cbi.pku.edu.cn/). The brownish red boxes indicate exons and single lines represent introns. The sizes of exons and introns can be estimated using the scale at the bottom. The figures a, b and c were redrawn in Adobe Illustrator (v25.2.3) (https://adobe.com/products/illustrator).

Supplementary Figure S5: Multiple sequence alignment of members of PenFKBP03 orthogroup representing the C-terminal KDEL retention sequence (red) was studied by using MUSCLE algorithm in Jalview software (v2.11.1.3) (http://www.jalview.org/).

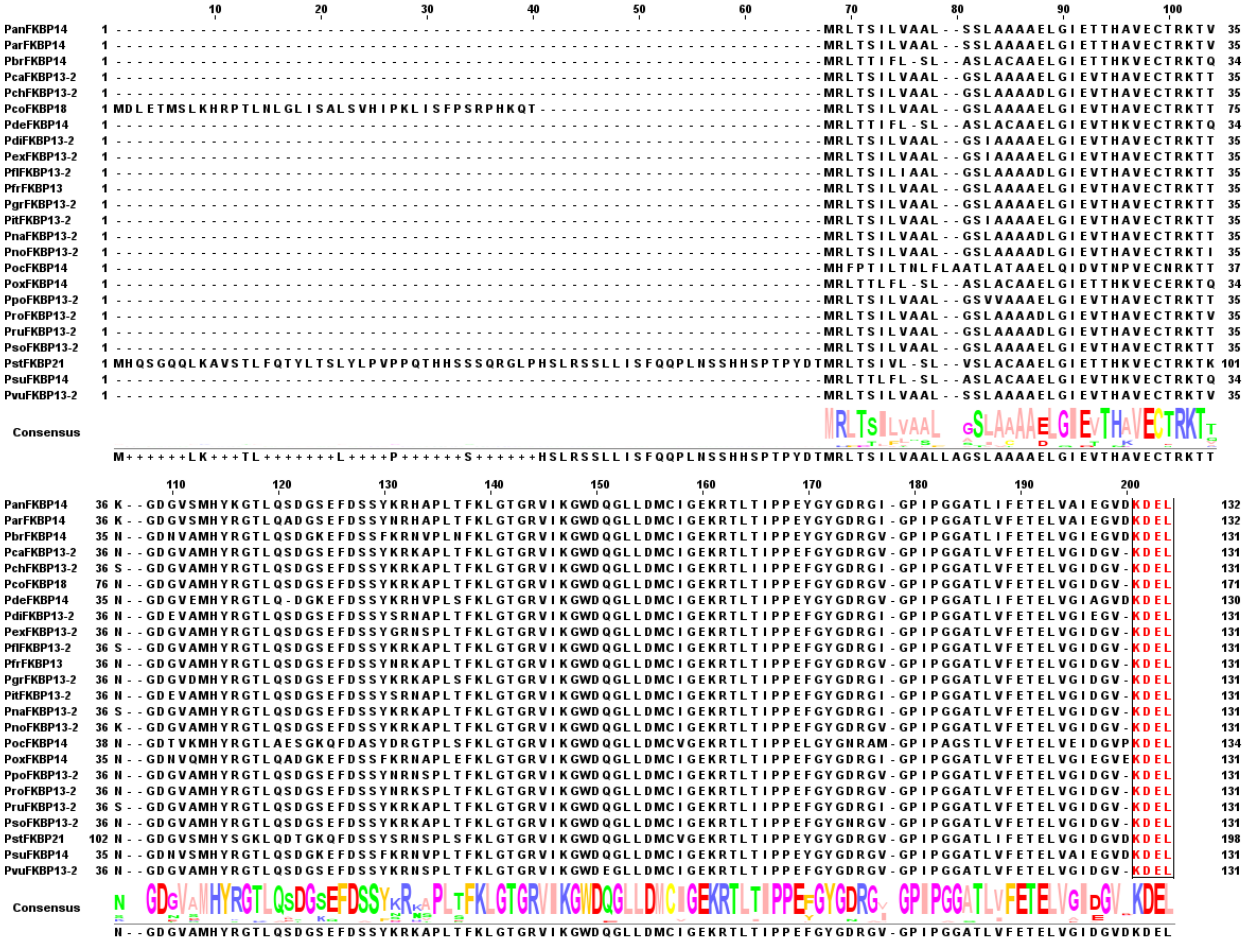

Supplementary Figure S6: Multiple sequence alignment of human FK506-binding protein 12 (hFKBP12) and *Penicillium* FKBP domains of different orthogroups was performed by using MUSCLE algorithm in Jalview software (v2.11.1.3) (http://www.jalview.org/). Secondary structure of hFKBP12 (PDB Id: 2ppn) was aligned with *Penicillium* FKBPs. Green arrows represent beta-sheets, and red objects signify alpha helix. Residues believed to be important for FK506 interaction are highlighted in black and the substitutions are represented in pink.

Orthogroup PenFKBP01

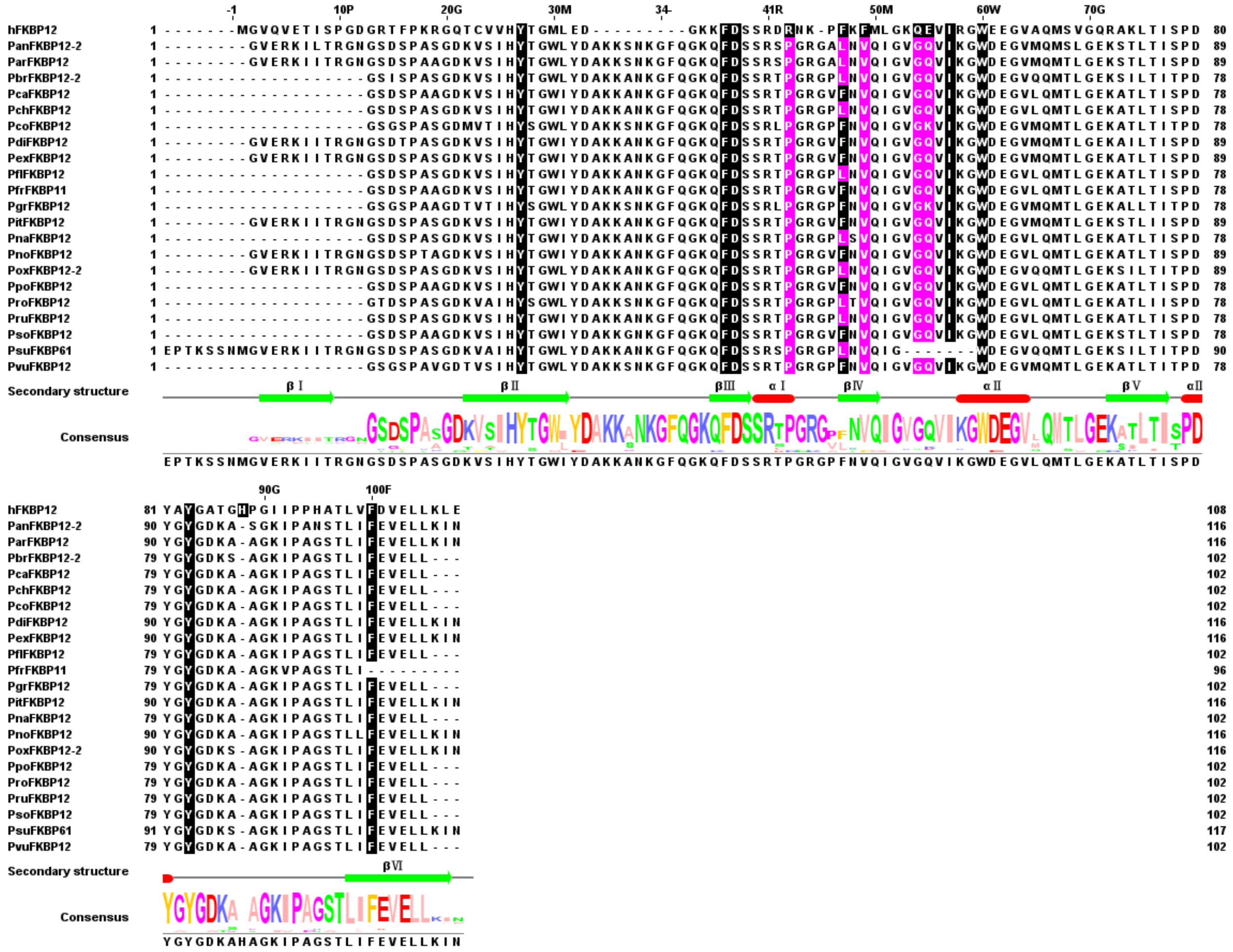

Continue on next page

Orthogroup PenFKBP02

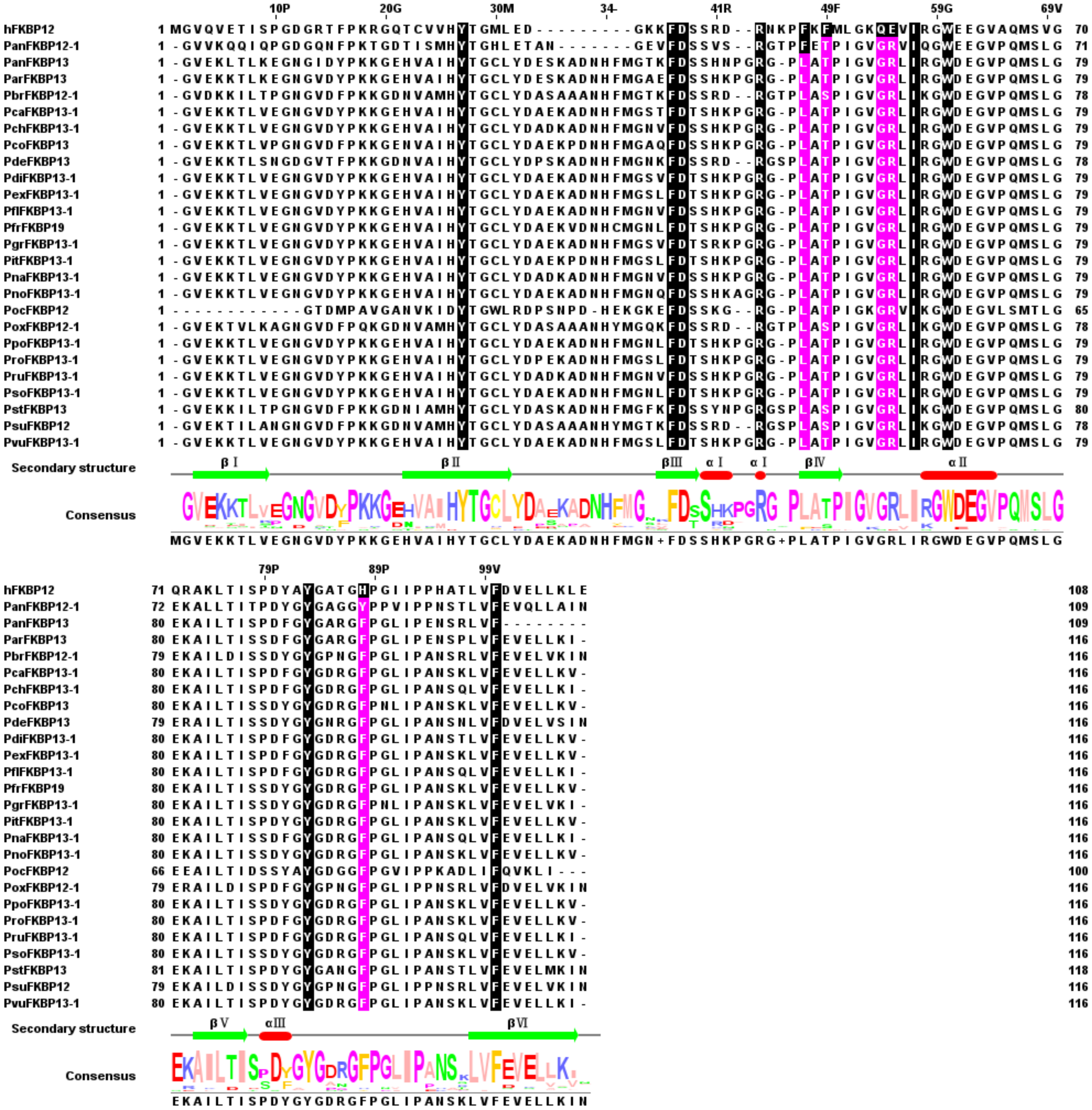

Continue on next page

Orthogroup PenFKBP03

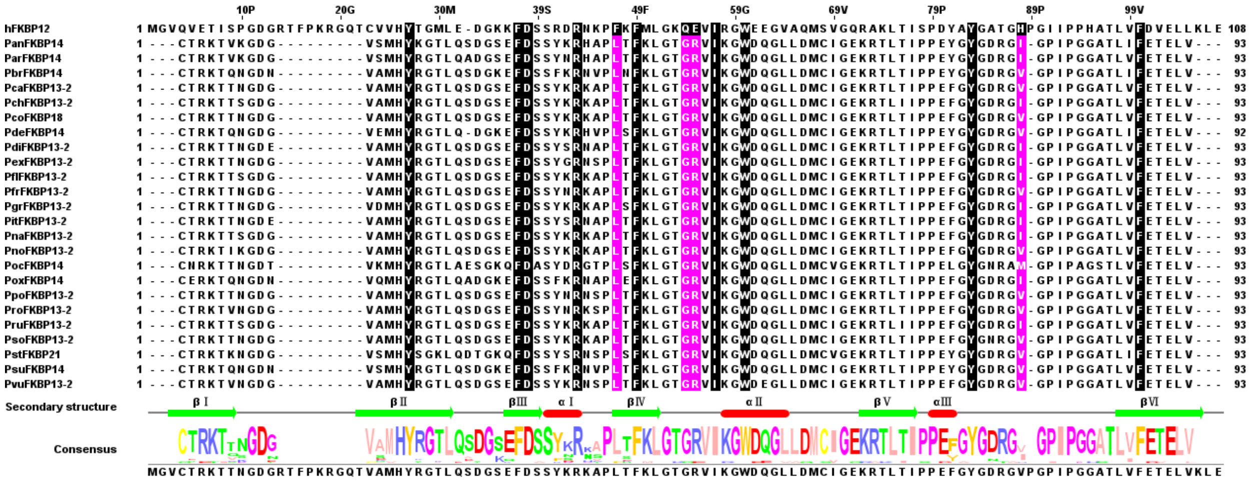

Orthogroup PenFKBP04

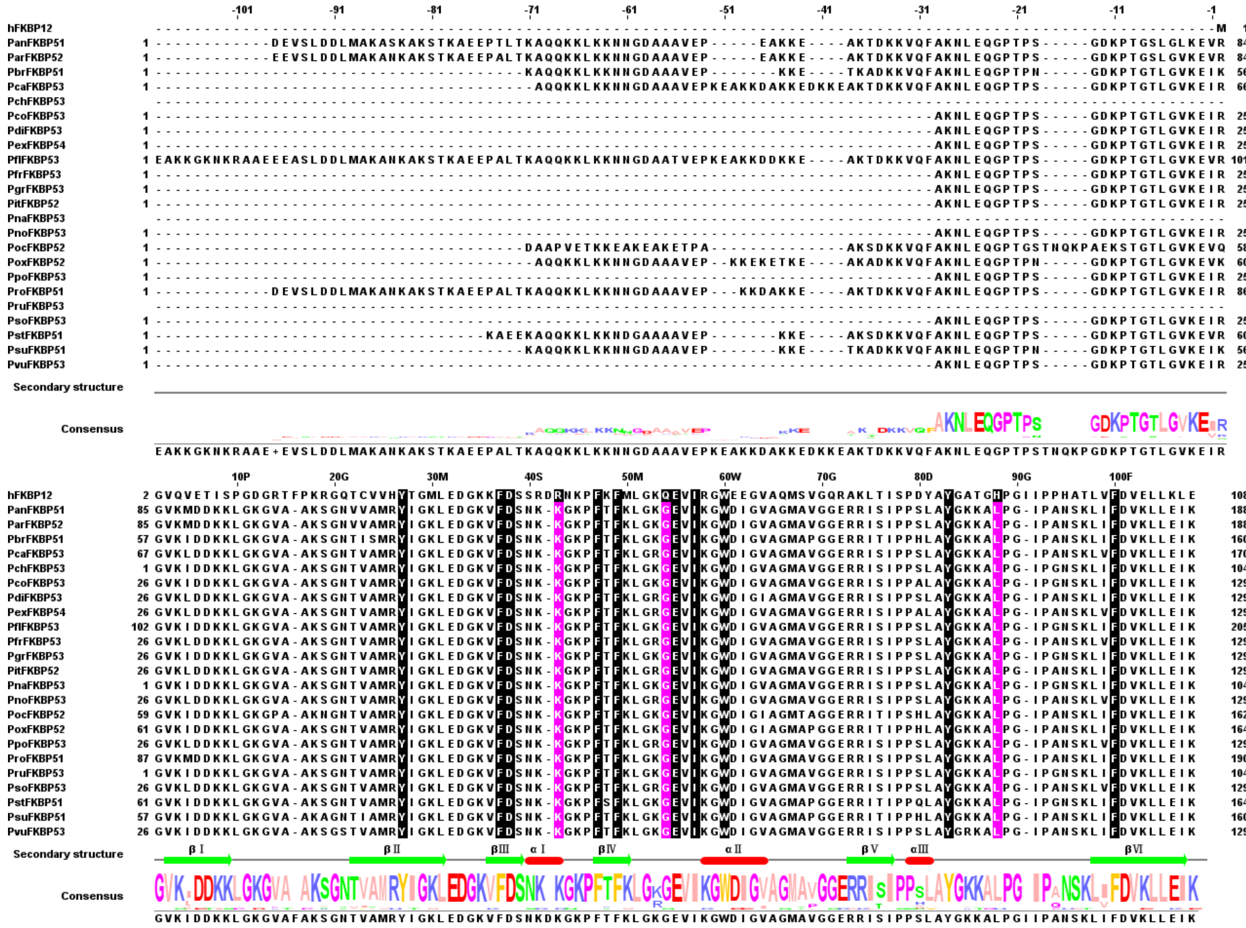

End of supplementary Figure S6

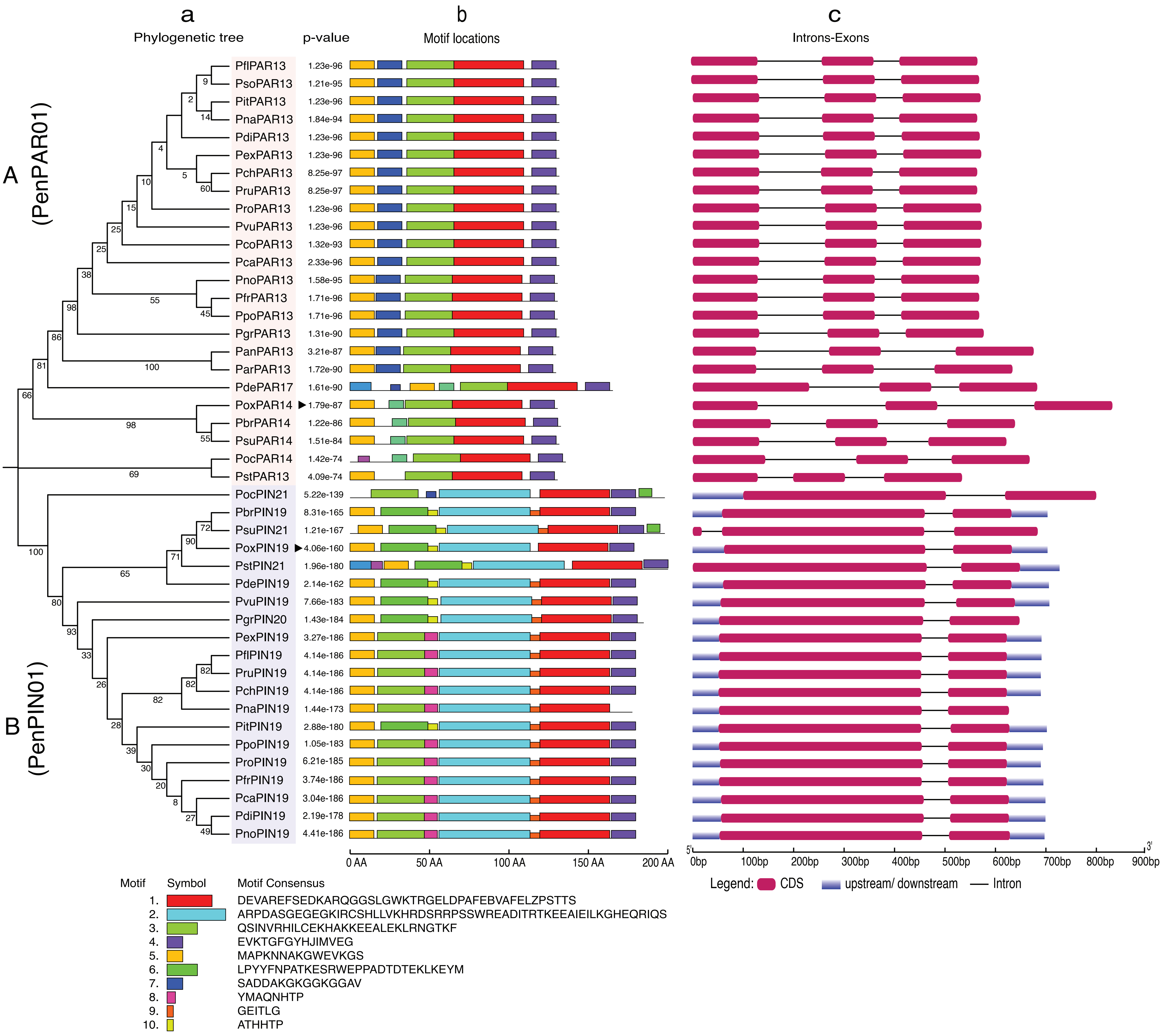

Supplementary Figure S7: Phylogenetic tree, encoded amino acid motifs and intron-exon structure of parvulin genes. (a) The phylogenetic tree of parvulins was constructed using the MEGA X (v10.1.7) software (http://www.megasoftware.net) with the NJ method. The constructed tree was annotated by the Iterative Tree of Life (http://itol.embl.de/). Percentages of bootstrap scores are indicated below the branches. Black triangles represent the parvulins of *Penicillium oxalicum*. (b) The different motifs were identified through MEME analysis (http://meme-suite.org/). The different-colored boxes, named at the bottom, with their consensus sequences represent the conserved motifs. Black lines represent the non-conserved sequences. (c) The corresponding exon-intron structures of parvulin genes were determined by the Gene Structure Display Server (http://gsds.cbi.pku.edu.cn/). The brownish red boxes indicate exons and single lines represent introns. The untranslated regions are indicated by blue boxes. The sizes of exons and introns can be estimated using the scale at the bottom. The figures a, b and c were redrawn in Adobe Illustrator (v25.2.3) (https://adobe.com/products/illustrator).

Supplementary Figure S8a: Multiple sequence alignment of human parvulin (hPAR14) and PPIase domains of *Penicillium* PenPAR01 parvulins was studied by employing MUSCLE algorithm in Jalview software (v2.11.1.3) (http://www.jalview.org/). Secondary structure of hPAR14 (PDB Id: 1EQ3) was aligned with *Penicillium* parvulins. Green arrows represent beta-sheets, and red objects signify the alpha helix. Residues believed to be important for PPIase activity are highlighted in black and substitutions are shown in pink.

Orthogroup PenPAR01

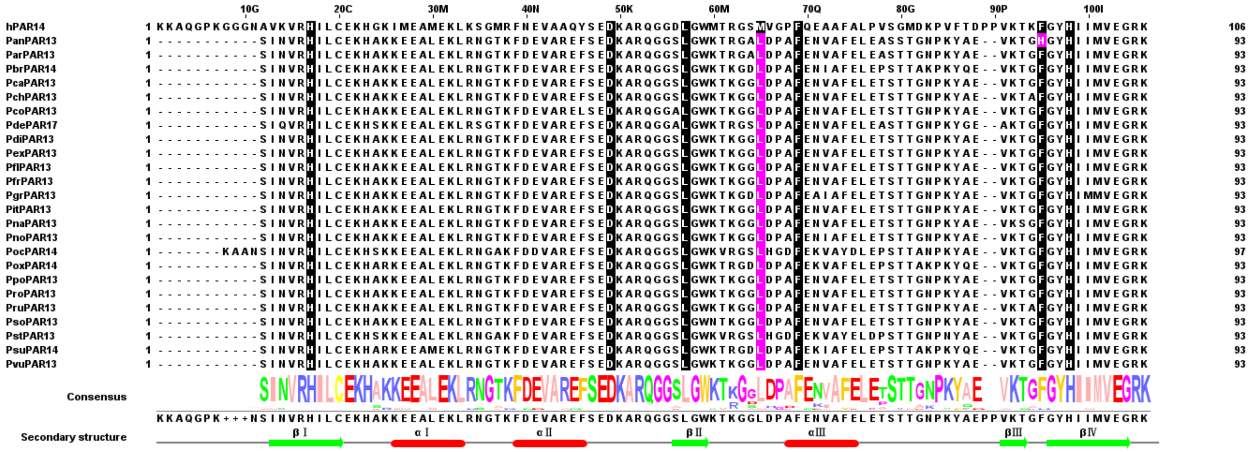

Supplementary Figure S8b: Multiple sequence alignment of human parvulin (hPIN1) and PPIase domains of *Penicillium* PenPIN01 parvulins was performed by using MUSCLE algorithm in Jalview software (v2.11.1.3) (http://www.jalview.org/). The secondary structure of hPIN1 (PDB Id: 1PIN) was aligned with *Penicillium* parvulins. Green arrows represent beta-sheets, and red objects signify the alpha helix. Residues believed to be important for PPIase activity are highlighted in black and the substitutions are depicted in pink.

Orthogroup PenPIN01

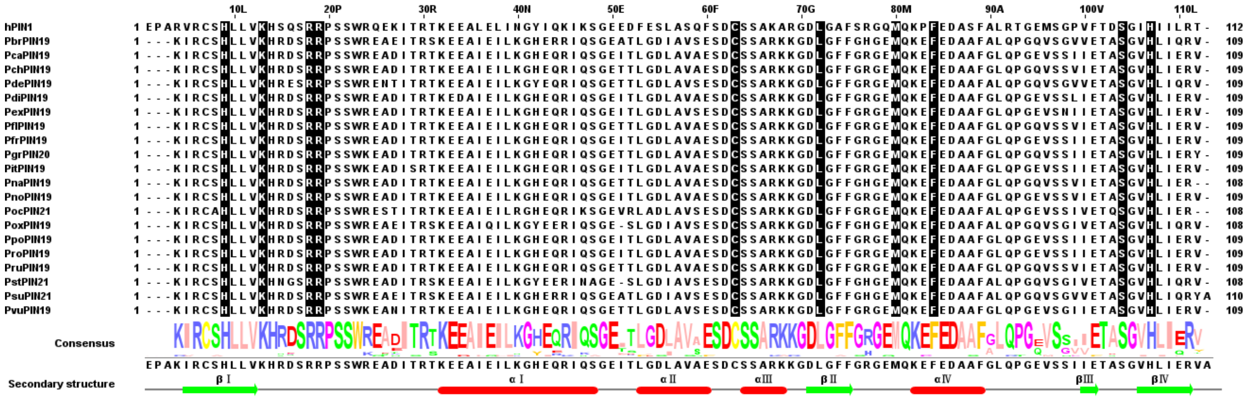

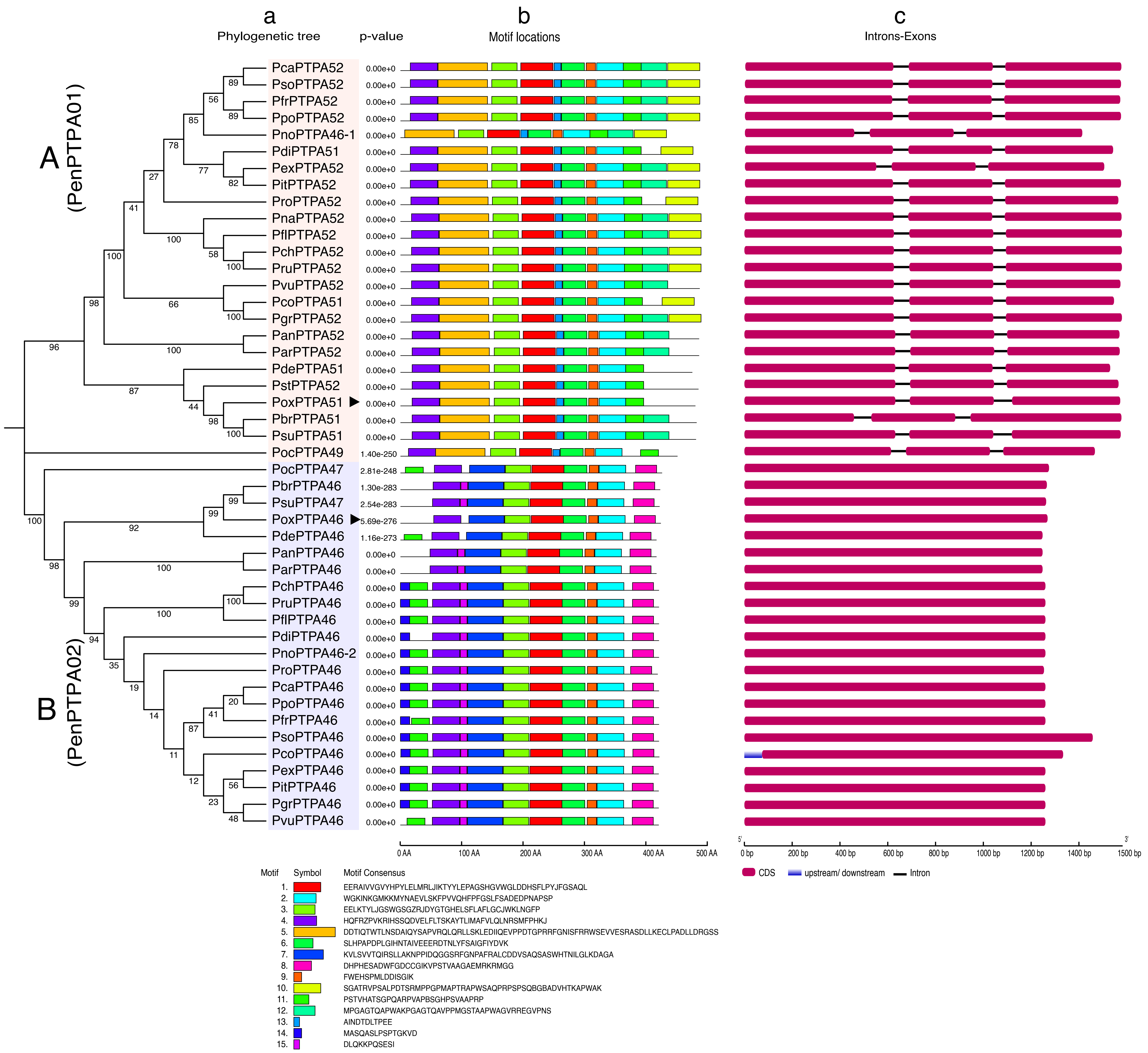

Supplementary Figure S9: Phylogenetic tree, encoded amino acid motifs and intron-exon structure of protein phosphatase 2A activators (PTPAs) genes. (a) The phylogenetic tree of PTPAs was constructed with the MEGA X (v10.1.7) software (http://www.megasoftware.net) with the NJ method. The constructed tree was annotated with the Iterative Tree of Life (http://itol.embl. de/). Percentages of bootstrap scores are indicated below the branches. Black triangles represent the PTPAs of *Penicillium oxalicum*. (b) The different motifs were identified through MEME analysis (http://meme-suite.org/). The different-colored boxes, named at the bottom, with their consensus sequences represent the conserved motifs. Black lines represent the non-conserved sequences. (c) The corresponding exon-intron structures of PTPA genes were determined by the Gene Structure Display Server (http://gsds.cbi.pku.edu.cn/). The brownish red boxes indicate exons and single lines represent introns. The untranslated regions are indicated by blue boxes. The sizes of exons and introns can be estimated using the scale at the bottom. The figures a, b and c were redrawn in Adobe Illustrator (v25.2.3) (https://adobe.com/products/illustrator).

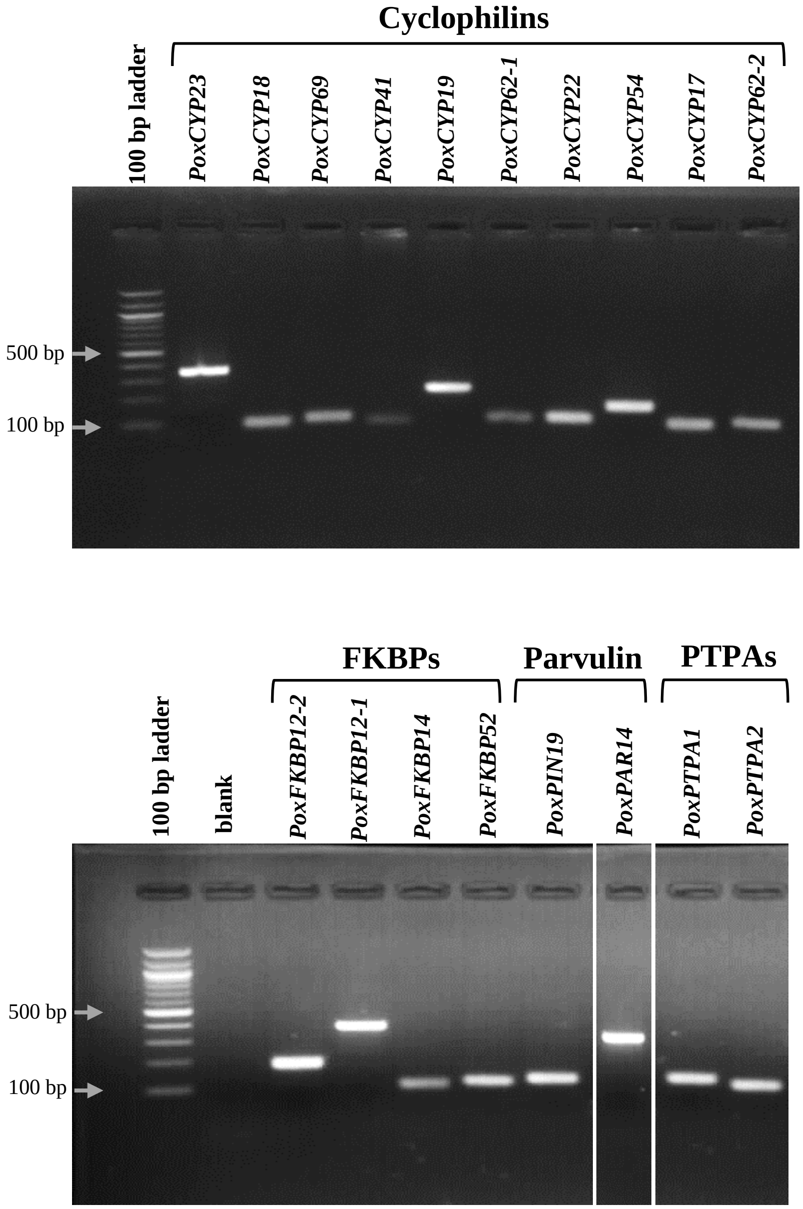

Supplementary Figure S10: The PCR amplification of genes encoding different cyclophilins, FK506-binding proteins (FKBPs), parvulins and protein phosphatase 2A phosphatase activators (PTPAs) using genomic DNA of *Penicillium oxalicum* as template. The amplified products were resolved on 2% agarose gel and stained with ethidium bromide.

Supplementary Figure S11: Representation of the conserved GVWGLD amino acid stretch (highlighted in black) in PenPTPA01 and PenPTPA02 members.

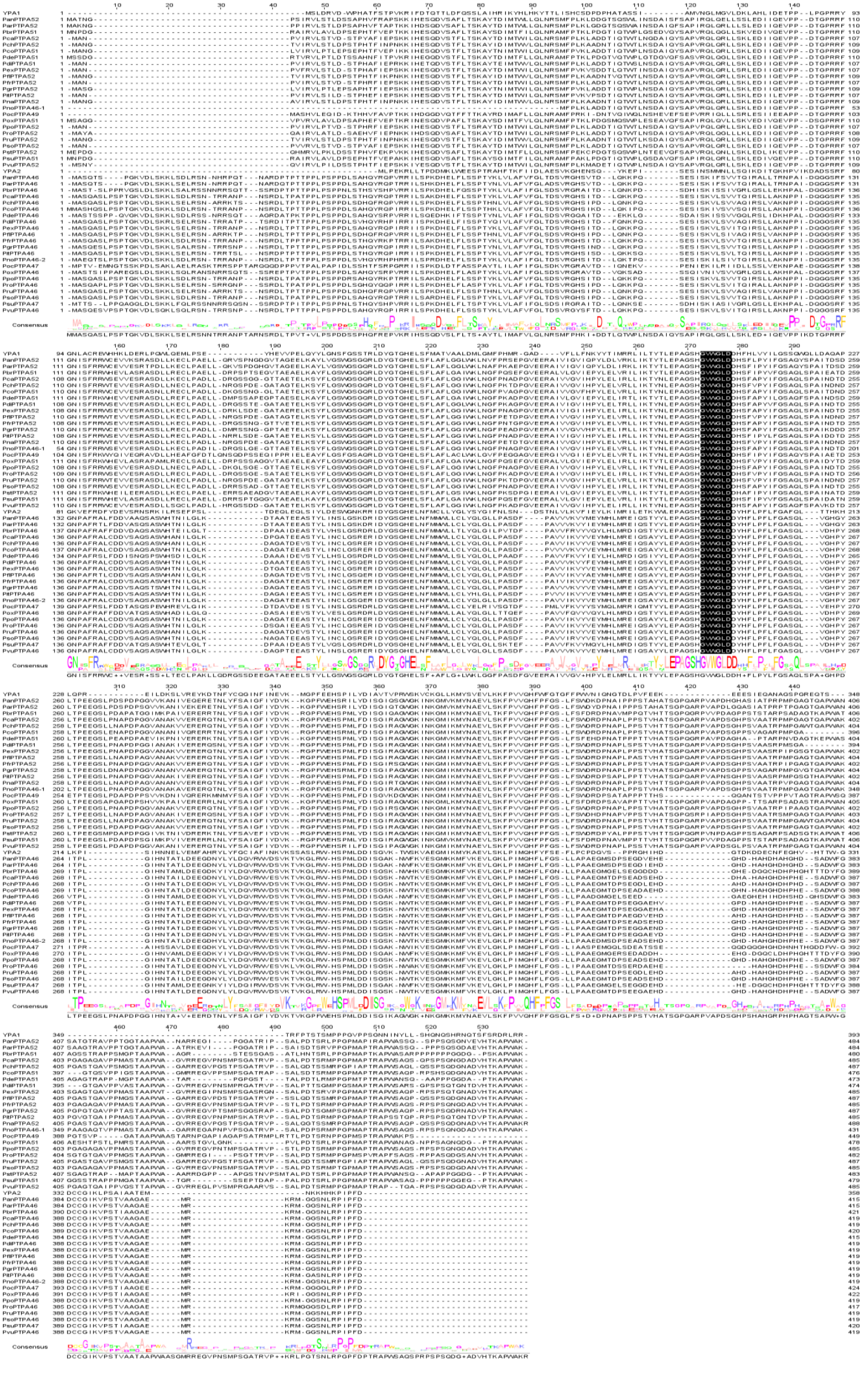

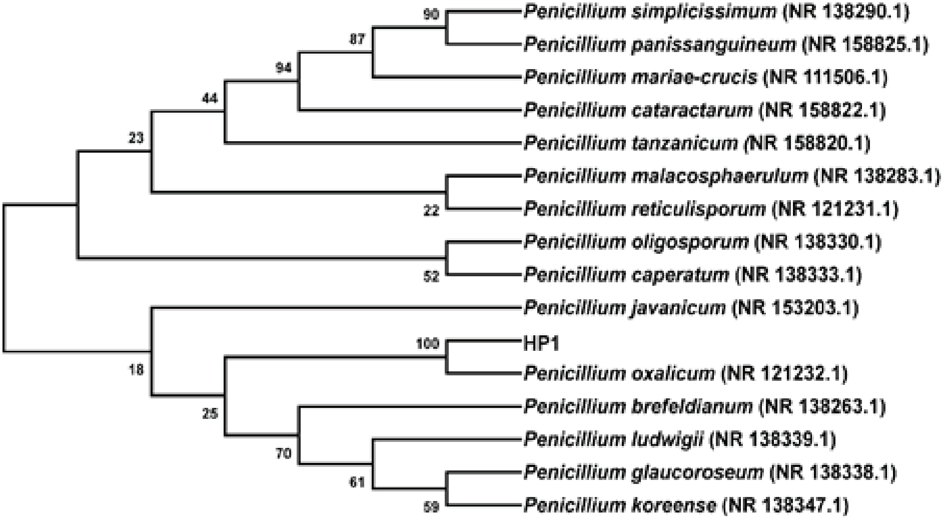

Supplementary Figure S12: Phylogenetic tree showing maximum similarity of 5.8S rRNA ITS sequence of the isolated *Penicillium* HP1 strain with *Penicillium oxalicum* (NR_121232.1).

Supplementary Table S1: *In silico* identification of cyclophilins, FK506 binding proteins (FKBPs), parvulins, and protein phosphatase 2A activators (PTPAs) in *Penicillium oxalicum.*

	S. No.	Proteins	Protein accession no.	Gene accession no.	AA residues	CDS (bp)	Introns	MW (kDa)	pI	Domains	Subcellular localization
Cyclophilins	1	PoxCYP17	EPS28411.1	KB644411.1	162	489	2	17.79	7.88	SD (CLD)	C
	2	PoxCYP19	EPS26058.1	KB644408.1	179	540	3	19.69	6.89	SD (CLD)	C
	3	PoxCYP18	EPS30376.1	KB644412.1	173	522	3	18.91	8.87	SD (CLD)	C
	4	PoxCYP22	EPS32942.1	KB644414.1	209	630	1	22.34	7.05	SD(CLD)	C
	5	PoxCYP23	EPS28743.1	KB644411.1	213	642	6	23.64	6.61	SD(CLD)	ER
	6	PoxCYP41	EPS26772.1	KB644409.1	372	1119	4	41.36	5.93	MD (CLD, TPR)	C
	7	PoxCYP54	EPS30915.1	KB644412.1	479	1440	3	54.51	6.31	MD (CLD, RRM)	N
	8	PoxCYP62-2	EPS34997.1	KB644415.1	567	1704	0	62.52	6.94	SD(CLD)	N
	9	PoxCYP62-1	EPS32379.1	KB644414.1	572	1719	2	62.35	8.19	MD (CLD, UBOX)	N
	10	PoxCYP69	EPS29520.1	KB644412.1	629	1849	2	69.82	6.22	MD (CLD, WD)	N
FKBPs	11	PoxFKBP12-2	EPS29132.1	KB644411.1	121	363	5	12.97	9.36	SD (FKBP)	C
	12	PoxFKBP12-1	EPS30742.1	KB644412.1	121	366	5	12.93	6.05	SD (FKBP)	C
	13	PoxFKBP14	EPS30398.1	KB644412.1	131	396	1	14.34	6.09	SD (FKBP)	ER
	14	PoxFKBP52	EPS34034.1	KB644415.1	484	1455	2	52.72	4.38	MD (FKBP, NPL)	N
Parvulins	15	PoxPAR14	EPS27836.1	KB644410.1	128	387	2	14.00	9.57	SD (Parvulin type PPIase domain)	N
	16	PoxPIN19	EPS29250.1	KB644411.1	175	528	3	19.43	5.98	MD (Parvulin type PPIase domain, WW)	N
PTPAs	17	PoxPTPA51	EPS34045.1	KB644415.1	478	1437	2	51.78	5.97	SD (PTPA)	C
	18	PoxPTPA46	EPS25240.1	KB644408.1	422	1269	0	46.80	5.92	SD (PTPA)	C

AA: amino acid; bp: base pair; C: cytoplasm; CDS: coding sequence; CLD: cyclophilin-like domain; ER: endoplasmic reticulum; FKBP: FK506 binding protein; kDa: kilodalton; MD: multidomain; MW: molecular weight; N: nucleus; NPL: nucleoplasmin like domain; pI: isoelectric point; PTPA: protein phosphatase 2A activator; RRM: RNA recognition motif; SD: single domain; TPR: tetratricopeptide repeat; U-box: U-box domain; WD: tryptophan-aspartate repeats; WW: Domain with 2 conserved tryptophan residues. Red color represents lowest value and green shows highest value.

Supplementary Table S2a: Comparative analysis of cyclophilins of orthogroup PenCYP01 in different species of *Penicillium*

**S. No.**	***Penicillium spp.***	**Protein name**	**Protein accession no.**	**AA**	**MW (kDa)**	**pI**	**CLD**	**Loc.**
1	*P. antarcticum*	PanCYP17	OQD79984.1	162	17.76	6.91	152 (4-155)	C
2	*P. arizonense*	ParCYP17	XP_022484041.1	162	17.77	7.88	152 (4-155)	C
3	*P. brasilianumn*	PbrCYP17	OOQ82861.1	162	17.85	6.91	152 (4-155)	C
4	*P. camemberti*	PcaCYP17-1	CRL18200.1	162	17.73	6.42	152 (4-155)	C
5	*P. chrysogenum*	PchCYP17	KZN90374.1	162	17.70	6.19	152 (4-155)	C
6	*P. coprophilum*	PcoCYP17	OQE41985.1	162	17.77	6.42	152 (4-155)	C
7	*P. decumbens*	PdeCYP17-2	OQD75611.1	161	17.78	7.91	152 (4-155)	C
8	*P. digitatum*	PdiCYP17-1	XP_014533444.1	162	17.67	6.42	152 (4-155)	C
9	*P. expansum*	PexCYP17	XP_016600102.1	162	17.72	6.42	152 (4-155)	C
10	*P. flavigenum*	PflCYP17	OQE25803.1	162	17.71	6.19	152 (4-155)	C
11	*P. freii*	PfrCYP17	KUM66478.1	162	17.70	5.93	152 (4-155)	C
12	*P. griseofulvum*	PgrCYP17	KXG54337.1	162	17.74	6.42	152 (4-155)	C
13	*P. italicum*	PitCYP17	KGO78174.1	162	17.70	6.42	152 (4-155)	C
14	*P. nalgiovense*	PnaCYP17	OQE84201.1	162	17.68	6.19	152 (4-155)	C
15	*P. nordicum*	PnoCYP17	KOS44138.1	162	17.90	5.94	152 (5-156)	C
16	*P. occitanis*	PocCYP17	PCG92000.1	162	17.69	6.51	152 (4-155)	C
17	*P. oxalicum*	PoxCYP17	EPS28411.1	162	17.79	7.88	152 (4-155)	C
18	*P. polonicum*	PpoCYP17	OQD64080.1	162	17.69	5.93	152 (4-155)	C
19	*P. roqueforti*	ProCYP17	CDM27334.1	162	17.74	6.42	152 (4-155)	C
20	*P. rubens*	PruCYP17	XP_002566234.1	162	17.70	6.19	152 (4-155)	C
21	*P. solitum*	PsoCYP17	OQD94594.1	162	17.72	6.42	152 (4-155)	C
22	*P. steckii*	PstCYP17-2	OQE17964.1	161	17.72	6.42	152 (4-155)	C
23	*P. subrubescens*	PsuCYP17	OKP14100.1	162	17.87	6.91	152 (4-155)	C
24	*P. vulpinum*	PvuCYP17	OQE06394.1	162	17.76	6.42	152 (4-155)	C

AA: amino acids; C: cytosol; CLD: cyclophilin-like domain; kDa: kilodalton; Loc: localization; MW: molecular weight; pI: isoelectric point. Red color represents lowest value and green shows highest value.

Supplementary Table S2b: Comparative analysis of cyclophilins of orthogroup PenCYP02 in different species of *Penicillium*

S. No.	***Penicillium spp.***	**Protein name**	**Protein accession no.**	**AA**	**MW (kDa)**	**pI**	**CLD**	**Loc**
1	*P. antarcticum*	-	-	-	-		-	-
2	*P. arizonense*	-	-	-	-		-	-
3	*P. brasilianumn*	PbrCYP19	OOQ86395.1	181	19.93	6.89	164 (17-180)	C
4	*P. camemberti*	PcaCYP18-2	CRL28434.1	173	18.94	6.41	164 (9-172)	C
5	*P. chrysogenum*	PchCYP19	KZN91406.1	173	18.99	6.41	164 (9-172)	C
6	*P. coprophilum*	PcoCYP22-2	OQE43656.1	206	22.68	8.61	164 (42-205)	C
7	*P. decumbens*	PdeCYP19	OQD78364.1	179	19.61	5.93	164 (15-178)	C
8	*P. digitatum*	PdiCYP19	XP_014532717.1	173	19	6.41	164 (9-172)	C
9	*P. expansum*	PexCYP18-3	XP_016595462.1	173	18.97	6.50	164 (9-172)	C
10	*P. flavigenum*	PflCYP18-2	OQE14397.1	173	18.98	6.41	164 (9-172)	C
11	*P. freii*	PfrCYP18-2	KUM57471.1	173	18.92	7.69	164 (9-172)	C
12	*P. griseofulvum*	PgrCYP18-2	KXG51141.1	173	18.96	6.41	164 (9-172)	C
13	*P. italicum*	PitCYP18-3	KGO65715.1	173	18.94	6.41	164 (9-172)	C
14	*P. nalgiovense*	PnaCYP19	OQE96439.1	174	19.14	6.41	165 (9-173)	C
15	*P. nordicum*	PnoCYP18-2	KOS45536.1	173	18.93	7.69	164 (9-172)	C
16	*P. occitanis*	PocCYP19	PCG88868.1	179	19.68	6.42	164 (15-178)	C
17	*P. oxalicum*	PoxCYP19	EPS26058.1	179	19.69	6.89	164 (15-178)	C
18	*P. polonicum*	PpoCYP20	OQD60173.1	187	20.39	7.69	164 (23-186)	C
19	*P. roqueforti*	ProCYP18-3	CDM36176.1	173	18.96	6.06	164 (9-172)	C
20	*P. rubens*	PruCYP19	XP_002565033.1	173	18.99	6.41	164 (9-172)	C
21	*P. solitum*	PsoCYP18-3	OQD91635.1	173	18.94	6.41	164 (9-172)	C
22	*P. steckii*	PstCYP19	OQE31507.1	180	19.83	6.41	164 (16-179)	C
23	*P. subrubescens*	PsuCYP19	OKO94276.1	181	19.92	6.41	164 (16-179)	C
24	*P. vulpinum*	PvuCYP18-2	OQE06984.1	173	18.98	7.69	164 (9-172)	C

AA: amino acids; C: cytosol; CLD: cyclophilin-like domain; kDa: kilodalton; Loc: localization; MW: molecular weight; pI: isoelectric point. Red color represents lowest value and green shows highest value.

Supplementary Table S2c: Comparative analysis of cyclophilins of orthogroup PenCYP03 in different species of *Penicillium*

S. No.	*Penicillium spp.*	Protein name	Protein accession no.	AA	MW (kDa)	pI	CLD	Loc
1	*P. antarcticum*	PanCYP18	OQD87062.1	173	18.53	7.78	155 (17-171)	C
2	*P. arizonense*	ParCYP18-2	XP_022493891.1	173	18.67	8.55	157 (15-171)	C
3	*P. brasilianumn*	PbrCYP18-2	OOQ83012.1	174	18.89	8.87	156 (17-172)	C
4	*P. camemberti*	PcaCYP18-1	CRL17145.1	173	18.49	7.80	156 (17-172)	C
5	*P. chrysogenum*	PchCYP18-2	KZN92198.1	174	18.74	8.57	158 (16-173)	C
6	*P. coprophilum*	-	-	-	-		-	-
7	*P. decumbens*	PdeCYP22-2	OQD77603.1	206	22.32	9.13	156 (50-205)	M
8	*P. digitatum*	PdiCYP18	XP_014531275.1	173	18.51	8.58	156 (17-172)	C
9	*P. expansum*	PexCYP18-2	XP_016593760.1	173	18.55	8.91	156 (17-172)	C
10	*P. flavigenum*	PflCYP22-2	OQE30221.1	212	22.86	9.14	158 (54-211)	M
11	*P. freii*	PfrCYP22-2	KUM63875.1	211	22.59	8.96	156 (55-210)	M
12	*P. griseofulvum*	-	-	-	-		-	-
13	*P. italicum*	PitCYP18-2	KGO77280.1	173	18.51	8.57	156 (17-172)	C
14	*P. nalgiovense*	PnaCYP22-2	OQE90455.1	212	22.8	8.96	158 (54-211)	M
15	*P. nordicum*	PnoCYP22-2	KOS39149.1	211	22.62	8.96	156 (55-210)	M
16	*P. occitanis*	PocCYP18	PCH03365.1	170	18.36	7.82	157 (13-169)	C
17	*P. oxalicum*	PoxCYP18	EPS30376.1	173	18.91	8.87	157 (16-172)	C
18	*P. polonicum*	-	-	-	-		-	-
19	*P. roqueforti*	ProCYP18-2	CDM33506.1	175	18.76	8.89	156 (19-174)	C
20	*P. rubens*	PruCYP18-2	XP_002562346.1	174	18.74	8.57	157 (16-172)	C
21	*P. solitum*	PsoCYP18-2	OQE00981.1	173	18.50	7.88	156 (17-172)	C
22	*P. steckii*	PstCYP22-2	OQE28769.1	205	22.41	9.32	158 (45-202)	M
23	*P. subrubescens*	PsuCYP18	OKO90908.1	174	18.81	8.89	158 (16-173)	C
24	*P. vulpinum*	PvuCYP22-2	OQE07592.1	211	22.55	8.95	155 (55-209)	M

AA: amino acids; C: cytosol; CLD: cyclophilin-like domain; kDa: kilodalton; Loc: localization; M: mitochondria; MW: molecular weight; pI: isoelectric point. Red color represents lowest value and green shows highest value.

Supplementary Table S2d: Comparative analysis of cyclophilins of orthogroup PenCYP04 in different species of *Penicillium*

**S. No.**	***Penicillium spp.***	**Protein name**	**Protein accession no.**	**AA**	**MW (kDa)**	**pI**	**CLD**	**Loc**
1	*P. antarcticum*	-	-	-	-	-	-	-
2	*P. arizonense*	ParCYP18-1	XP_022490207.1	167	18.05	6.91	159 (8-166)	C
3	*P. brasilianumn*	-	-	-	-	-	-	-
4	*P. camemberti*	PcaCYP17-2	CRL24897.1	168	17.97	7.93	160 (8-167)	C
5	*P. chrysogenum*	PchCYP18-1	KZN93469.1	168	18.12	6.91	160 (8-167)	C
6	*P. coprophilum*	PcoCYP18	OQE46629.1	168	18.06	6.91	157 (11-167)	C
7	*P. decumbens*	PdeCYP17-1	OQD74591.1	162	17.76	8.98	157 (5-161)	C
8	*P. digitatum*	PdiCYP17-2	XP_014530821.1	168	17.91	6.28	158 (10-167)	C
9	*P. expansum*	PexCYP18-1	XP_016602773.1	168	18.03	7.94	160 (8-167)	C
10	*P. flavigenum*	PflCYP18-1	OQE23312.1	168	18.08	6.07	160 (8-167)	C
11	*P. freii*	PfrCYP18-1	KUM65238.1	168	18.00	7.94	160 (8-167)	C
12	*P. griseofulvum*	PgrCYP18-1	KXG50639.1	168	18.15	7.96	160 (8-167)	C
13	*P. italicum*	PitCYP18-1	KGO75618.1	168	18.06	6.91	160 (8-167)	C
14	*P. nalgiovense*	PnaCYP18	OQE89777.1	168	18.05	6.91	160 (8-167)	C
15	*P. nordicum*	PnoCYP18-1	KOS47894.1	168	18.07	6.91	157 (11-167)	C
16	*P. occitanis*	-	-	-	-	-	-	-
17	*P. oxalicum*	-	-	-	-	-	-	-
18	*P. polonicum*	PpoCYP18	OQD66776.1	168	18.06	7.93	159 (9-167)	C
19	*P. roqueforti*	ProCYP18-1	CDM34751.1	168	18.10	7.92	157 (11-167)	C
20	*P. rubens*	PruCYP18-1	XP_002561605.1	168	18.12	6.91	160 (8-167)	C
21	*P. solitum*	PsoCYP18-1	OQD90319.1	168	18.02	7.93	160 (8-167)	C
22	*P. steckii*	PstCYP17-1	OQE19056.1	165	17.63	8.52	159 (6-164)	C
23	*P. subrubescens*	-	-	-	-	-	-	-
24	*P. vulpinum*	PvuCYP18-1	OQE00183.1	168	18.09	7.94	158 (10-167)	C

AA: amino acids; C: cytosol; CLD: cyclophilin-like domain; kDa: kilodalton; Loc: localization; MW: molecular weight; pI: isoelectric point. Red color represents lowest value and green shows highest value.

Supplementary Table S2e: Comparative analysis of cyclophilins of orthogroup PenCYP05 in different species of *Penicillium*

**S. No.**	***Penicillium spp.***	**Protein name**	**Protein accession no.**	**AA**	**MW (kDa)**	**pI**	**CLD**	**Loc**
1	*P. antarcticum*	PanCYP22	OQD90958.1	209	22.35	6.37	196 (2-197)	C
2	*P. arizonense*	ParCYP22	XP_022491254.1	209	22.41	6.44	190 (2-191)	C
3	*P. brasilianumn*	PbrCYP22	OOQ88537.1	209	22.07	6.43	190 (2-191)	C
4	*P. camemberti*	PcaCYP22	CRL28228.1	208	22.11	7.09	195 (2-196)	C
5	*P. chrysogenum*	PchCYP22	KZN89557.1	208	22.22	7.09	191 (2-192)	C
6	*P. coprophilum*	PcoCYP22-1	OQE44220.1	208	22.29	6.43	192 (2-193)	C
7	*P. decumbens*	PdeCYP22-1	OQD76602.1	209	22.23	8.99	195 (2-196)	C
8	*P. digitatum*	PdiCYP22	XP_014535704.1	208	22.21	6.70	189 (2-190)	C
9	*P. expansum*	PexCYP22	XP_016597377.1	208	22.18	6.70	195 (2-196)	C
10	*P. flavigenum*	PflCYP22-1	OQE22789.1	208	22.25	7.09	192 (2-193)	C
11	*P. freii*	PfrCYP22-1	KUM63178.1	208	22.10	7.09	195 (2-196)	C
12	*P. griseofulvum*	PgrCYP26	KXG52801.1	246	26.57	8.63	195 (40-234)	C
13	*P. italicum*	PitCYP22	KGO76335.1	208	22.17	7.09	195 (2-196)	C
14	*P. nalgiovense*	PnaCYP22-1	OQE86138.1	211	22.55	7.79	192 (5-196)	C
15	*P. nordicum*	PnoCYP22-1	KOS41948.1	208	22.12	7.09	195 (2-196)	C
16	*P. occitanis*	PocCYP22-2	PCH07164.1	212	22.92	6.35	191 (2-192)	C
17	*P. oxalicum*	PoxCYP22	EPS32942.1	209	22.34	7.05	199 (2-200)	C
18	*P. polonicum*	PpoCYP22	OQD62183.1	208	22.12	7.09	195 (2-196)	C
19	*P. roqueforti*	ProCYP22	CDM31360.1	208	22.26	6.70	189 (2-190)	C
20	*P. rubens*	PruCYP22	XP_002569008.1	208	22.22	7.09	191 (2-192)	C
21	*P. solitum*	PsoCYP22	OQD97960.1	208	22.10	7.09	195 (2-196)	C
22	*P. steckii*	PstCYP22-1	OQE27527.1	209	22.31	7.79	191 (2-192)	C
23	*P. subrubescens*	PsuCYP22	OKP15010.1	209	22.22	7.83	200 (2-201)	C
24	*P. vulpinum*	PvuCYP22-1	OQE00699.1	208	22.18	6.22	197 (2-198)	C

AA: amino acids; C: cytosol; CLD: cyclophilin-like domain; kDa: kilodalton; Loc: localization; MW: molecular weight; pI: isoelectric point. Red color represents lowest value and green shows highest value.

Supplementary Table S2f: Comparative analysis of cyclophilins of orthogroup PenCYP06 in different species of *Penicillium*

S. No.	*Penicillium spp.*	Protein name	Protein accession no.	AA	MW (kDa)	pI	CLD	Loc
1	*P. antarcticum*	PanCYP32	OQD85283.1	288	32.44	5.69	159 (38-196)	ER
2	*P. arizonense*	ParCYP29	XP_022489368.1	264	29.61	5.84	159 (38-196)	ER
3	*P. brasilianumn*	PbrCYP29	OOQ83251.1	265	29.72	5.28	162 (33-194)	ER
4	*P. camemberti*	PcaCYP29	CRL18667.1	270	29.88	5.54	159 (38-196)	ER
5	*P. chrysogenum*	PchCYP23	KZN90748.1	214	23.73	6.45	159 (38-196)	ER
6	*P. coprophilum*	PcoCYP29	OQE41773.1	268	29.68	6.15	159 (38-196)	ER
7	*P. decumbens*	PdeCYP29	OQD74213.1	263	29.55	6.46	159 (38-196)	ER
8	*P. digitatum*	PdiCYP30	XP_014537840.1	268	29.91	5.39	159 (38-196)	ER
9	*P. expansum*	PexCYP29	XP_016595680.1	268	29.73	5.70	159 (38-196)	ER
10	*P. flavigenum*	PflCYP29	OQE28056.1	269	29.84	6.15	159 (38-196)	ER
11	*P. freii*	PfrCYP29	KUM58093.1	268	29.71	5.54	159 (38-196)	ER
12	*P. griseofulvum*	PgrCYP29	KXG52606.1	271	29.96	6.93	159 (38-196)	ER
13	*P. italicum*	PitCYP36	KGO76066.1	337	36.98	5.05	159 (106-264)	ER
14	*P. nalgiovense*	PnaCYP37	OQE91950.1	340	37.71	7.02	159 (106-264)	ER
15	*P. nordicum*	PnoCYP23	KOS45894.1	212	23.45	7.84	158 (39-196)	ER
16	*P. occitanis*	PocCYP22-1	PCH02504.1	207	22.82	6.21	160 (38-197)	ER
17	*P. oxalicum*	PoxCYP23	EPS28743.1	213	23.64	6.61	159 (38-196)	ER
18	*P. polonicum*	PpoCYP29	OQD68220.1	268	29.66	5.54	159 (38-196)	ER
19	*P. roqueforti*	ProCYP29	CDM27801.1	265	29.53	5.72	159 (38-196)	ER
20	*P. rubens*	PruCYP29	XP_002565809.1	265	29.4	6.46	159 (38-196)	ER
21	*P. solitum*	PsoCYP29	OQD95123.1	268	29.73	5.55	159 (38-196)	ER
22	*P. steckii*	PstCYP29	OQE27867.1	266	29.6	5.91	159 (38-196)	ER
23	*P. subrubescens*	PsuCYP29	OKO95621.1	266	29.77	5.48	159 (38-196)	ER
24	*P. vulpinum*	PvuCYP29	OQE06008.1	261	29.21	6.54	159 (38-196)	ER

AA: amino acids; CLD: cyclophilin-like domain; ER: endoplasmic reticulum; kDa: kilodalton; Loc: localization; MW: molecular weight; pI: isoelectric point. Red color represents lowest value and green shows highest value.

Supplementary Table S2g: Comparative analysis of cyclophilins of orthogroup PenCYP07 in different species of *Penicillium*

S. No.	*Penicillium spp.*	Protein name	Protein accession no.	AA	MW (kDa)	pI	CLD	TPR domain	Loc
1	*P. antarcticum*	PanCYP42	OQD82010.1	385	42.30	5.79	179 (9-187)	34 (310-343)	C
2	*P. arizonense*	ParCYP39	XP_022485085.1	361	39.95	5.56	155 (9-163)	34 (298-331)	C
3	*P. brasilianumn*	PbrCYP41	OOQ84995.1	379	41.97	6.06	168 (12-179)	34 (309-342)	C
4	*P. chrysogenum*	-	-	-	-	-	-	-	-
5	*P. coprophilum*	-	-	-	-	-	-	-	-
6	*P. decumbens*	-	-	-	-	-	-	-	-
7	*P. digitatum*	PdiCYP41	XP_014538180.1	372	41.27	5.92	165 (9-173)	34 (309-342)	C
8	*P. flavigenum*	-	-	-	-	-	-	-	-
9	*P. freii*	PfrCYP41	KUM59399.1	372	41.14	6.06	165 (9-173)	34 (309-342)	C
10	*P. nalgiovense*	PnaCYP41	OQE72690.1	373	41.20	5.78	165 (9-173)	34 (309-342)	C
11	*P. nordicum*	PnoCYP39	KOS45707.1	355	39.35	5.90	149 (9-157)	34 (198-231)	C
12	*P. occitanis*	PocCYP40	PCG90929.1	371	40.79	5.93	165 (9-173)	34 (308-341)	C
13	*P. oxalicum*	PoxCYP41	EPS26772.1	372	41.36	5.93	166 (9-174)	34 (309-342)	C
14	*P. polonicum*	-	-	-	-	-	-	-	-
15	*P. roqueforti*	ProCYP41	CDM32075.1	372	41.18	6.29	165 (9-173)	39 (309-348)	C
16	*P. rubens*	PruCYP41	XP_002563022.1	372	41.16	5.91	165 (9-173)	34 (309-342)	C
17	*P. steckii*	PstCYP40	OQE29790.1	373	40.96	6.16	164 (11-174)	34 (310-343)	C
18	*P. subrubescens*	PsuCYP38	OKO97230.1	343	38.18	5.56	128 (17-144)	34 (280-313)	C
19	*P. vulpinum*	PvuCYP33	OQE09577.1	302	33.40	5.50	102 (1-103)	34 (239-272)	C
20 a	*P. camemberti*	PcaCYP7	CRL20414.1	73	7.94	7.95	56 (10-66)	-	
20 b	*P. camemberti*	PcaCYP33	CRL20413.1	303	33.38	5.33	102 (1-103)	34 (239-272)	C
21 a	*P. expansum*	PexCYP8	XP_016602215.1	73	7.95	9.65	58 (10-68)	-	
21 b	*P. expansum*	PexCYP33	XP_016602216.1	302	33.34	5.42	102 (1-103)	34 (239-272)	C
22 a	*P. griseofulvum*	PgrCYP7	KXG45958.1	73	7.96	9.36	57 (10-67)	-	
22 b	*P. griseofulvum*	PgrCYP33	KXG45957.1	302	33.33	5.43	102 (1-103)	34 (239-272)	C
23 a	*P. italicum*	PitCYP8	KGO77399.1	73	8.00	8.98	58 (10-68)	-	
23 b	*P. italicum*	PitCYP33	KGO77400.1	302	33.31	5.42	102 (1-103)	34 (239-272)	C
24 a	*P. solitum*	PsoCYP7	OQD88558.1	73	7.96	7.93	56 (10-66)	-	
24 b	*P. solitum*	PsoCYP33	OQD88555.1	302	33.34	5.42	102 (1-103)	34 (239-272)	C

AA: amino acids; C: cytosol; CLD: cyclophilin-like domain; kDa: kilodalton; Loc: localization; MW: molecular weight; pI: isoelectric point; TPR: tetratricopeptide repeat. Partial proteins (may nonfunctional due to incomplete CLD domains) are highlighted in gray color). Red color represents lowest value and green shows highest value.

Supplementary Table S2h: Comparative analysis of cyclophilins of orthogroup PenCYP08 in different species of *Penicillium*

S. No.	*Penicillium spp.*	Protein name	Protein accession no.	AA	MW (kDa)	pI	CLD	RRM domain	Loc
1	*P. antarcticum*	PanCYP52	OQD83063.1	457	52.52	5.59	170 (2-171)	83 (245-327)	N
2	*P. arizonense*	ParCYP67	XP_022485256.1	589	67.55	5.32	170 (135-304)	83 (378-460)	N
3	*P. brasilianumn*	PbrCYP55	OOQ87529.1	484	55.37	5.98	170 (2-171)	71 (250-320)	N
4	*P. camemberti*	PcaCYP51	CRL29953.1	452	51.73	5.50	170 (2-171)	83 (245-327)	N
5	*P. chrysogenum*	PchCYP50	KZN88869.1	444	50.81	5.76	170 (2-171)	83 (245-327)	N
6	*P. coprophilum*	-	-	-	-	-	-	-	-
7	*P. decumbens*	-	-	-	-	-	-	-	-
8	*P. digitatum*	PdiCYP47	XP_014532860.1	428	47.73	4.68	170 (2-171)	83 (245-372)	N
9	*P. expansum*	PexCYP49	XP_016596415.1	444	49.44	4.56	170 (2-171)	83 (245-327)	N
10	*P. flavigenum*	PflCYP51	OQE27131.1	448	51.24	5.49	170 (2-171)	83 (245-327)	N
11	*P. freii*	PfrCYP51	KUM63769.1	452	51.77	5.42	170 (2-71)	83 (245-327)	N
12	*P. griseofulvum*	PgrCYP50	KXG51883.1	439	50.01	5.68	170 (2-171)	83 (245-327)	N
13	*P. italicum*	PitCYP51	KGO77145.1	464	51.82	4.66	170 (2-171)	83 (248-326)	N
14	*P. nalgiovense*	PnaCYP48	OQE74228.1	436	48.65	4.63	170 (2-171)	83 (245-327)	N
15	*P. nordicum*	PnoCYP51	KOS37305.1	452	51.78	5.42	170 (2-171)	83 (245-327)	N
16	*P. occitanis*	PocCYP49	PCH07127.1	452	49.84	4.72	169 (2-170)	83 (245-326)	N
17	*P. oxalicum*	PoxCYP54	EPS30915.1	479	54.51	6.31	170 (2-171)	79 (248-326)	N
18	*P. polonicum*	-	-	-	-	-	-	-	-
19	*P. roqueforti*	ProCYP50	CDM29153.1	444	50.59	5.65	170 (2-171)	83 (245-327)	N
20	*P. rubens*	PruCYP50	XP_002568227.1	444	50.81	5.76	169 (2-170)	83 (245-327)	N
21	*P. solitum*	PsoCYP51	OQD90267.1	451	51.51	5.49	170 (2-171)	83 (248-326)	N
22	*P. steckii*	PstCYP51	OQE29724.1	448	51.45	5.73	169 (2-170)	83 (244-326)	N
23	*P. subrubescens*	PsuCYP54	OKP13294.1	478	54.4	5.85	170 (2-172)	83 (245-327)	N
24	*P. vulpinum*	PvuCYP51	OQE05593.1	448	51.09	5.76	170 (2-171)	83 (245-327)	N

AA: amino acids; C: cytosol; CLD: cyclophilin-like domain; kDa: kilodalton; Loc: localization; MW: molecular weight; N: nucleus; pI: isoelectric point; RRM: RNA recognition motif. Red color represents lowest value and green shows highest value.

Supplementary Table S2i: Comparative analysis of cyclophilins of orthogroup PenCYP09 in different species of *Penicillium*

S. No.	***Penicillium spp.***	**Protein name**	**Protein accession no.**	**AA**	**MW (kDa)**	**pI**	**CLD**	**Loc**
1	*P. antarcticum*	PanCYP60	OQD79390.1	545	60.17	8.26	171 (14-184)	N
2	*P. arizonense*	ParCYP60	XP_022492184.1	547	60.35	8.70	171 (14-184)	N
3	*P. brasilianumn*	PbrCYP60	OOQ88953.1	554	60.77	8.28	171 (14-184)	N
4	*P. camemberti*	PcaCYP60	CRL27976.1	544	60.14	6.49	171 (14-184)	N
5	*P. chrysogenum*	PchCYP60	KZN89067.1	543	60.07	8.59	171 (14-184)	N
6	*P. coprophilum*	-	-	-	-	-	-	-
7	*P. decumbens*	-	-	-	-	-	-	-
8	*P. digitatum*	PdiCYP60	XP_014533773.1	546	60.66	8.60	171 (14-184)	N
9	*P. expansum*	PexCYP60	XP_016596923.1	545	60.39	7.10	171 (14-184)	N
10	*P. flavigenum*	-	-	-	-	-	-	-
11	*P. freii*	PfrCYP60	KUM59792.1	544	60.29	8.02	171 (14-184)	N
12	*P. griseofulvum*	PgrCYP60	KXG51474.1	544	60.34	8.27	171 (14-184)	N
13	*P. italicum*	PitCYP60	KGO76191.1	545	60.33	7.10	171 (14-184)	N
14	*P. nalgiovense*	PnaCYP59	OQE82711.1	540	59.76	8.01	171 (14-184)	N
15	*P. nordicum*	PnoCYP65	KOS39527.1	594	65.78	8.76	171 (64-234)	N
16	*P. occitanis*	PocCYP62	PCG98687.1	565	62.80	7.18	171 (14-184)	N
17	*P. oxalicum*	PoxCYP62-2	EPS34997.1	567	62.52	6.94	171 (14-184)	N
18	*P. polonicum*	PpoCYP60	OQD60247.1	544	60.20	6.77	171 (14-184)	N
19	*P. roqueforti*	ProCYP60	CDM29813.1	545	60.18	7.59	171 (14-184)	N
20	*P. rubens*	PruCYP60	XP_002568462.1	543	60.07	8.59	171 (14-184)	N
21	*P. solitum*	PsoCYP60	OQD86006.1	544	60.24	7.07	171 (14-184)	N
22	*P. steckii*	PstCYP60	OQE22485.1	544	60.18	8.03	171 (14-184)	N
23	*P. subrubescens*	PsuCYP60	OKP12258.1	553	60.82	8.46	171 (14-184)	N
24	*P. vulpinum*	-	-	-	-	-	-	-

AA: amino acids; C: cytosol; CLD: cyclophilin-like domain; kDa: kilodalton; Loc: localization; MW: molecular weight; N: nucleus; pI: isoelectric point. Red color represents lowest value and green shows highest value.

Supplementary Table S2j: Comparative analysis of cyclophilins of orthogroup PenCYP10 in different species of *Penicillium*

S. No.	*Penicillium spp.*	Protein name	Protein accession no.	AA	MW (kDa)	pI	CLD	U box domain/ PP2C domain*	Loc
							Start (AA)	Start (AA)	
1	*P. antarcticum*	PanCYP62	OQD86755.1	574	62.09	7.71	157 (308-464)	72 (45-116)	N
2	*P. arizonense*	ParCYP126	XP_022493905.1	1183	126.82	5.97	157 (308-464)	72 (45-116)<br>332 (749-1080) *	N
3	*P. brasilianumn*	PbrCYP66	OOQ85525.1	613	66.47	8.93	156 (305-460)	72 (86-157)	N
4	*P. camemberti*	PcaCYP62	CRL26136.1	573	62.15	8.45	157 (305-461)	72 (45-116)	N
5	*P. chrysogenum*	PchCYP62	KZN88196.1	571	62.1	7.24	157 (304-460)	72 (45-116)	N
6	*P. coprophilum*	PcoCYP62	OQE45095.1	573	62.26	7.73	157 (305-461)	72 (45-116)	N
7	*P. decumbens*	-	-	-	-	-	-	-	-
8	*P. digitatum*	PdiCYP62	XP_014532357.1	573	62.12	8.46	157 (305-461)	72 (45-116)	N
9	*P. expansum*	PexCYP62	XP_016602528.1	573	62.15	8.45	157 (305-461)	72 (45-116)	N
10	*P. flavigenum*	PflCYP61	OQE32414.1	571	61.91	8.45	157 (304-460)	72 (45-116)	N
11	*P. freii*	PfrCYP62	KUM62866.1	572	62.07	7.73	157 (305-461)	72 (45-116)	N
12	*P. griseofulvum*	PgrCYP62	KXG47693.1	572	62.01	8.63	157 (304-460)	72 (45-116)	N
13	*P. italicum*	PitCYP65	KGO67615.1	604	65.63	8.40	157 (305-461)	72 (45-116)	N
14	*P. nalgiovense*	PnaCYP62	OQE94151.1	571	62.08	7.74	157 (304-460)	72 (45-116)	N
15	*P. nordicum*	PnoCYP62	KOS47498.1	573	62.18	7.24	157 (305-461)	72 (45-116)	N
16	*P. occitanis*	PocCYP64	PCG96974.1	581	64.06	8.63	156 (309-464)	73 (45-117)	N
17	*P. oxalicum*	PoxCYP62-1	EPS32379.1	572	62.35	8.19	157 (308-464)	74 (42-115)	N
18	*P. polonicum*	PpoCYP62	OQD71244.1	572	62.06	7.73	157 (305-461)	72 (45-116)	N
19	*P. roqueforti*	ProCYP62	CDM31005.1	573	62.34	8.18	157 (305-461)	72 (45-116)	N
20	*P. rubens*	PruCYP62	XP_002567457.1	571	62.1	7.24	157 (304-460)	72 (45-116)	N
21	*P. solitum*	PsoCYP62	OQE03396.1	573	62.12	8.45	157 (305-461)	72 (45-116)	N
22	*P. steckii*	PstCYP62	OQE31306.1	576	62.67	8.16	157 (308-464)	72 (45-116)	N
23	*P. subrubescens*	PsuCYP61	OKP14436.1	571	61.96	6.92	156 (307-462)	72 (45-116)	N
24	*P. vulpinum*	PvuCYP62	OQE07304.1	573	62.44	8.18	157 (349-505)	72 (45-116)	N

AA: amino acids; C: cytosol; CLD: cyclophilin-like domain; kDa: kilodalton; Loc: localization; MW: molecular weight; pI: isoelectric point; PP2C: Protein phosphatase 2 C. Red color represents lowest value and green shows highest value.

Supplementary Table S2k: Comparative analysis of cyclophilins of orthogroup PenCYP11 in different species of *Penicillium*

S. No.	*Penicillium spp.*	Protein name	Protein accession no.	AA	MW (kDa)	pI	CLD	WD repeats	Loc
1	*P. antarcticum*	PanCYP69	OQD83865.1	627	69.22	6.05	150 (477-626)	231 (72-303)	N
2	*P. arizonense*	ParCYP69	XP_022483413.1	627	69.26	6.13	150 (477-626)	231 (72-303)	N
3	*P. brasilianumn*	PbrCYP72	OOQ91529.1	657	72.46	6.28	150 (507-656)	164 (102-266)	N
4	*P. camemberti*	PcaCYP69	CRL18291.1	627	**69.56**	6.14	150 (477-626)	164 (72-236)	N
5	*P. chrysogenum*	PchCYP69	KZN90456.1	627	69.51	6.38	150 (477-626)	237 (73-310)	N
6	*P. coprophilum*	PcoCYP69	OQE42181.1	627	69.56	6.10	150 (477-626)	231 (72-303)	N
7	*P. decumbens*	PdeCYP69	OQD76092.1	629	69.56	6.35	150 (479-628)	231 (74-305)	N
8	*P. digitatum*	PdiCYP69	XP_014533512.1	627	69.6	6.28	150 (477-626)	164 (72-236)	N
9	*P. expansum*	PexCYP69	XP_016602951.1	627	69.48	6.19	150 (477-626)	237 (73-310)	N
10	*P. flavigenum*	PflCYP69	OQE25910.1	627	69.48	6.28	150 (477-626)	164 (72-236)	N
11	*P. freii*	PfrCYP69	KUM60563.1	627	69.52	6.10	150 (477-626)	238 (72-310)	N
12	*P. griseofulvum*	PgrCYP69	KXG54260.1	627	69.5	6.19	150 (477-626)	238 (72-310)	N
13	*P. italicum*	PitCYP69	KGO77757.1	627	69.55	6.19	150 (477-626)	238 (72-310)	N
14	*P. nalgiovense*	PnaCYP69	OQE89342.1	627	69.61	6.24	150 (477-626)	238 (72-310)	N
15	*P. nordicum*	PnoCYP69	KOS44474.1	627	69.3	6.19	150 (477-626)	164 (72-236)	N
16	*P. occitanis*	PocCYP70	PCG89336.1	631	70.39	6.12	151 (480-631)	221 (75-296)	N
17	*P. oxalicum*	PoxCYP69	EPS29520.1	629	69.82	6.22	150 (479-628)	254 (74-328)	N
18	*P. polonicum*	PpoCYP69	OQD63852.1	627	69.49	6.10	150 (477-626)	238 (72-310)	N
19	*P. roqueforti*	ProCYP69	CDM27419.1	627	69.41	6.62	150 (477-626)	163 (72-235)	N
20	*P. rubens*	PruCYP69	XP_002566139.1	627	69.51	6.38	150 (477-626)	238 (72-310)	N
21	*P. solitum*	PsoCYP69	OQD97237.1	627	69.52	6.14	150 (477-626)	164 (72-236)	N
22	*P. steckii*	PstCYP69	OQE28371.1	629	69.81	6.03	150 (479-628)	164 (74-238)	N
23	*P. subrubescens*	PsuCYP69	OKP10113.1	629	69.57	6.19	150 (479-628)	164 (74-238)	N
24	*P. vulpinum*	PvuCYP69	OQE00515.1	627	69.47	6.22	150 (477-626)	238 (72-310)	N

AA: amino acids; CLD: cyclophilin-like domain; kDa: kilodalton; Loc: localization; MW: molecular weight; N: nucleus; pI: isoelectric point; WD: tryptophan-aspartate repeats. Red color represents lowest value and green shows highest value.

Supplementary Table S2l: Comparative analysis of cyclophilins of orthogroup PenCYP12 in different species of *Penicillium*

S. No	*Penicillium spp.*	Protein name	Protein accession no.	AA	MW (kDa)	pI	CLD domain	GIT_SHD2 domain	Loc
1	*P. antarcticum*	-	-	-	-	-	-	-	-
2	*P. arizonense*	-	-	-	-	-	-	-	-
3	*P. brasilianumn*	-	-	-	-	-	-	-	-
4	*P. camemberti*	-	-	-	-	-	-	-	-
5	*P. chrysogenum*	-	-	-	-	-	-	-	-
6	*P. coprophilum*	PcoCYP121	OQE46439.1	1110	121.93	5.78	155 (954-1108)	28 (130-157)<br>27 (188-214)	C
7	*P. decumbens*	-	-	-	-	-	-	-	-
8	*P. digitatum*	-	-	-	-	-	-	-	-
9	*P. expansum*	-	-	-	-	-	-	-	-
10	*P. flavigenum*	-	-	-	-	-	-	-	-
11	*P. freii*	-	-	-	-	-	-	-	-
12	*P. griseofulvum*	PgrCYP121	KXG45441.1	1108	121.89	5.60	155 (952-1106)	29 (126-156)<br>27 (186-212)	C
13	*P. italicum*	-	-	-	-	-	-	-	-
14	*P. nalgiovense*	-	-	-	-	-	-	-	-
15	*P. nordicum*	-	-	-	-	-	-	-	-
16	*P. occitanis*	-	-	-	-	-	-	-	-
17	*P. oxalicum*	-	-	-	-	-	-	-	-
18	*P. polonicum*	-	-	-	-	-	-	-	-
19	*P. roqueforti*	-	-	-	-	-	-	-	-
20	*P. rubens*	-	-	-	-	-	-	-	-
21	*P. solitum*	-	-	-	-	-	-	-	-
22	*P. steckii*	-	-	-	-	-	-	-	-
23	*P. subrubescens*	-	-	-	-	-	-	-	-
24	*P. vulpinum*	-	-	-	-	-	-	-	-

AA: amino acids; C: cytosol; CLD: cyclophilin-like domain; GIT_SDH: Spa2 homology domain (SHD) of GIT {G-protein coupled receptor (GPCR)-kinase-interacting protein}; kDa: kilodalton; Loc: localization; MW: molecular weight; pI: isoelectric point.

Supplementary Table S3a: Percentage identity and similarity of cyclophilins of orthogroup PenCYP01

	1	2	3	4	5	6	7	8	9	10	11	12	13	14	15	16	17	18	19	20	21	22	23	24	25
1. hCYPA		48	47.1	46.2	47.1	47.1	47.1	47.4	47.7	47.7	47.7	45.9	47.1	47.7	47.7	47.7	48.8	47.1	45.9	47.7	47.1	47.7	49.4	46.2	46.5
2. PanCYP17	62.4		97.5	91.4	90.1	90.7	88.9	89.5	90.7	90.7	90.1	88.3	89.5	90.7	90.7	89.6	84.6	87.7	88.9	90.1	90.7	90.7	90.7	92	89.5
3. ParCYP17	61.8	99.4		91.4	92	91.4	90.1	89.5	92.6	92.6	92	90.1	90.7	92.6	92.6	90.8	85.8	87.7	90.7	92	91.4	92.6	91.4	92	91.4
4. PbrCYP17	61.2	98.1	97.5		88.3	87.7	86.4	91.4	87	87.7	88.3	85.2	87.7	88.3	87.7	86.5	87.7	92.6	85.8	87	87.7	87.7	93.2	97.5	87.7
5. PcaCYP17-1	61.8	96.3	96.9	95.1		96.9	96.3	86.4	98.1	99.4	97.5	95.7	98.8	98.8	98.1	96.9	85.2	86.4	96.3	98.8	96.9	99.4	87	87	99.4
6. PchCYP17	62.4	95.7	96.3	94.4	99.4		93.2	87.7	96.3	97.5	99.4	95.1	95.7	96.9	98.8	96.3	85.2	87	95.7	96.9	100	97.5	86.4	87.7	96.3
7. PcoCYP17	61.8	95.7	95.7	95.1	98.1	97.5		87	94.4	95.7	93.8	92	96.3	95.1	94.4	93.3	82.7	85.8	92.6	95.1	93.2	95.7	86.4	85.2	95.7
8. PdeCYP17-2	63	96.9	96.3	96.9	93.2	94.4	93.2		87	87	88.3	85.8	85.8	87	87.7	87.1	87.7	92	86.4	86.4	87.7	87	90.7	92	85.8
9. PdiCYP17-1	62.4	96.9	97.5	95.1	99.4	98.8	97.5	93.8		98.8	96.9	95.1	96.9	98.8	97.5	96.3	84.6	87	95.7	98.1	96.3	98.8	87.7	87.7	97.5
10. PexCYP17	61.8	96.3	96.9	95.1	100	99.4	98.1	93.2	99.4		98.1	96.3	98.1	99.4	98.8	97.5	84.6	87	96.9	99.4	97.5	100	87.7	87.7	98.8
11. PflCYP17	62.4	95.7	96.3	94.4	99.4	100	97.5	94.4	98.8	99.4		95.7	96.3	97.5	99.4	96.9	85.8	87.7	96.3	97.5	99.4	98.1	87	88.3	96.9
12. PfrCYP17	61.8	95.1	95.7	93.8	98.8	99.4	96.9	93.2	98.1	98.8	99.4		94.4	95.7	96.3	95.1	84.6	84.6	99.4	95.7	95.1	96.3	85.2	85.2	95.7
13. PgrCYP17	61.2	96.3	96.3	95.1	99.4	98.8	97.5	93.2	98.8	99.4	98.8	98.1		97.5	96.9	95.7	84	85.8	95.1	97.5	95.7	98.1	86.4	86.4	98.1
14. PitCYP17	62.4	96.9	97.5	95.1	100	99.4	98.1	93.8	100	100	99.4	98.8	99.4		98.1	96.9	84.6	87	96.3	98.8	96.9	99.4	87.7	87.7	98.1
15. PnaCYP17	62.4	95.7	96.3	94.4	99.4	100	97.5	94.4	98.8	99.4	100	99.4	98.8	99.4		97.5	85.2	87	96.9	98.1	98.8	98.8	86.4	87.7	97.5
16. PnoCYP17	63	95.7	96.3	94.5	98.2	98.8	96.3	93.9	97.5	98.2	98.8	98.2	97.5	98.2	98.8		84.7	85.9	95.7	96.9	96.3	97.5	85.9	87.1	96.3
17. PocCYP17	63	93.8	94.4	92.6	91.4	92	90.7	94.4	92	91.4	92	91.4	90.7	92	92	92		87	84	84	85.2	84.6	85.2	87	84.6
18. PoxCYP17	60.6	97.5	96.9	98.1	95.1	94.4	95.1	97.5	95.7	95.1	94.4	93.8	95.1	95.7	94.4	94.5	92.6		85.2	86.4	87	87	91.4	93.2	85.8
19. PpoCYP17	61.8	95.1	95.7	93.8	98.8	99.4	96.9	93.2	98.1	98.8	99.4	100	98.1	98.8	99.4	98.2	91.4	93.8		96.3	95.7	96.9	85.8	85.8	96.3
20. ProCYP17	61.8	96.3	96.9	95.1	100	99.4	98.1	93.2	99.4	100	99.4	98.8	99.4	100	99.4	98.2	91.4	95.1	98.8		96.9	99.4	87	87	98.1
21. PruCYP17	62.4	95.7	96.3	94.4	99.4	100	97.5	94.4	98.8	99.4	100	99.4	98.8	99.4	100	98.8	92	94.4	99.4	99.4		97.5	86.4	87.7	96.3
22. PsoCYP17	61.8	96.3	96.9	95.1	100	99.4	98.1	93.2	99.4	100	99.4	98.8	99.4	100	99.4	98.2	91.4	95.1	98.8	100	99.4		87.7	87.7	98.8
23. PstCYP17-2	61.8	98.8	98.1	98.1	96.3	95.7	96.3	95.7	96.9	96.3	95.7	95.1	96.3	96.9	95.7	95.1	93.2	96.9	95.1	96.3	95.7	96.3		93.8	86.4
24. PsuCYP17	61.2	97.5	96.9	98.8	93.8	93.2	93.8	96.3	94.4	93.8	93.2	92.6	93.8	94.4	93.2	93.3	92	97.5	92.6	93.8	93.2	93.8	97.5		86.4
25. PvuCYP17	61.8	96.3	96.9	95.1	100	99.4	98.1	93.2	99.4	100	99.4	98.8	99.4	100	99.4	98.2	91.4	95.1	98.8	100	99.4	100	96.3	93.8	

Similarity

Identity

Red color represents lowest and green shows highest percentage value.

Supplementary Table S3b: Percentage identity and similarity of cyclophilins of orthogroup PenCYP02

	1	2	3	4	5	6	7	8	9	10	11	12	13	14	15	16	17	18	19	20	21	22	23
1. hCYPA		53.8	55.2	54.6	47.3	53.9	54.6	55.2	55.2	55.2	55.7	55.2	54.9	55.7	54.4	53.9	51.1	54.6	54.6	55.2	54.1	53.3	56.3
2. PbrCYP19	67.4		85.6	86.7	77.2	89.5	86.2	86.2	87.3	85.6	86.2	86.7	86.3	85.6	80.7	91.2	83.4	85.6	86.7	85.6	90.6	96.1	86.7
3. PcaCYP18-2	68.8	91.2		95.4	80.1	88.8	97.1	97.7	96	98.3	95.4	97.7	94.8	97.7	82.1	84.4	90.9	97.1	95.4	99.4	85	85.6	96.5
4. PchCYP19	68.8	91.2	98.8		82	87.7	95.4	96	99.4	95.4	94.8	97.1	98.3	95.4	80.4	87.2	88.2	96.5	100	95.4	86.7	87.3	96
5. PcoCYP22-2	58.3	81.1	82.5	83.5		78.2	80.1	80.6	82.5	80.6	81.6	81.6	81.6	80.6	70.9	76.8	83.5	81.1	82	80.1	76.7	78.2	82
6. PdeCYP19	67.6	94.5	93.9	92.7	82.5		88.8	89.4	88.3	89.4	89.4	89.4	87.2	88.3	81.6	85.5	87.2	89.9	87.7	89.4	88.9	86.7	88.8
7. PdiCYP19	68.8	91.7	98.8	98.8	82.5	92.7		98.3	96	97.7	95.4	98.3	94.8	96.5	81	85.5	90.4	97.7	95.4	97.7	85.6	86.2	96.5
8. PexCYP18-3	68.8	91.7	99.4	99.4	83	93.3	99.4		96.5	98.3	96	98.8	95.4	97.1	81	85.5	90.9	98.3	96	98.3	85.6	86.2	97.1
9. PflCYP18-2	68.8	91.2	98.8	100	83.5	92.7	98.8	99.4		96	95.4	97.7	98.9	96	81	87.7	88.8	97.1	99.4	96	87.2	87.8	96.5
10. PfrCYP18-2	68.8	91.2	100	98.8	82.5	93.9	98.8	99.4	98.8		96	98.3	94.8	98.8	81	84.9	92.5	97.7	95.4	98.8	85	85.6	97.7
11. PgrCYP18-2	69.4	92.8	98.8	98.8	83.5	94.4	98.8	99.4	98.8	98.8		96	94.3	96	81	86.6	88.8	97.1	94.8	95.4	86.7	86.2	98.3
12. PitCYP18-3	68.8	91.7	99.4	99.4	83	93.3	99.4	100	99.4	99.4	99.4		96.6	97.1	81	86.6	90.9	98.3	97.1	98.3	85.6	86.7	97.1
13. PnaCYP19	68.4	90.6	97.7	98.9	83	92.2	97.7	98.3	98.9	97.7	97.7	98.3		94.8	80.6	86.7	87.8	96	98.3	94.8	86.7	86.8	95.4
14. PnoCYP18-2	68.8	90.6	99.4	98.3	82	93.3	98.3	98.8	98.3	99.4	98.3	98.8	97.1		81	84.9	91.4	96.5	95.4	97.7	85	85.6	97.7
15. PocCYP19	66.5	88.4	89.9	89.4	78.2	91.6	89.9	89.9	89.4	89.9	89.9	89.9	89.4	89.4		81.7	77.5	81	80.4	82.1	82.2	81.2	81.6
16. PoxCYP19	67.6	93.9	92.7	93.9	84	93.9	93.3	93.9	93.9	92.7	94.4	93.9	93.3	92.2	89.9		81.8	85.5	87.2	84.4	89.4	90.6	87.2
17. PpoCYP20	63.6	88.8	92.5	91.4	85.9	90.4	91.4	92	91.4	92.5	91.4	92	90.9	92	86.1	89.3		90.4	88.2	91.4	82.4	82.9	90.4
18. ProCYP18-3	68.8	91.2	98.8	100	83.5	92.7	98.8	99.4	100	98.8	98.8	99.4	98.9	98.3	89.4	93.9	91.4		96.5	97.7	86.1	86.7	97.1
19. PruCYP19	68.8	91.2	98.8	100	83.5	92.7	98.8	99.4	100	98.8	98.8	99.4	98.9	98.3	89.4	93.9	91.4	100		95.4	86.7	87.3	96
20. PsoCYP18-3	68.8	91.2	100	98.8	82.5	93.9	98.8	99.4	98.8	100	98.8	99.4	97.7	99.4	89.9	92.7	92.5	98.8	98.8		85	85.6	96.5
21. PstCYP19	66.7	94.5	90.6	91.1	80.6	94.4	90.6	90.6	91.1	90.6	91.1	90.6	91.1	90	92.8	95	88.2	91.1	91.1	90.6		89.5	86.7
22. PsuCYP19	66.3	97.8	91.7	92.8	82.5	92.8	92.3	92.3	92.8	91.7	92.8	92.3	92.3	91.2	87.3	93.4	89.3	92.8	92.8	91.7	94.5		87.3
23. PvuCYP18-2	69.4	92.3	98.8	98.8	83.5	93.9	98.8	99.4	98.8	98.8	100	99.4	97.7	98.3	89.4	94.4	91.4	98.8	98.8	98.8	91.1	92.8	

Identity

Similarity

Supplementary Table S3c: Percentage identity and similarity of cyclophilins of orthogroup PenCYP03

	1	2	3	4	5	6	7	8	9	10	11	12	13	14	15	16	17	18	19	20	21	22
1. hCYPA		60.9	60.9	58.3	59.2	58.3	50.2	60.9	60.3	48.4	48.6	59.8	48.8	48.6	59	59.2	58	58.3	59.2	49.5	57.7	49.1
2. PanCYP18	72.8		94.8	83.9	83.2	82.8	68.9	86.7	84.4	67	67.8	85.5	66	67.3	66.3	83.2	82.9	82.8	83.2	68.3	81.6	69.7
3. ParCYP18-2	72.3	97.7		83.9	84.4	83.3	69.4	85.5	83.8	67.5	69.7	84.4	66.5	69.2	66.9	82.1	82.3	83.3	84.4	67.8	81	69.7
4. PbrCYP18-2	71.3	90.8	89.7		85.1	82.8	72.3	84.5	85.1	68.4	69.2	84.5	67.5	68.7	65.4	86.2	82.3	82.8	85.1	74.1	93.7	69.7
5. PcaCYP18-1	74.6	90.8	90.2	89.7		91.4	67.5	95.4	94.2	73.6	79.6	96.5	73.1	79.6	66.9	83.2	92.6	91.4	98.8	70.2	83.9	79.1
6. PchCYP18-2	71.3	89.7	90.2	89.1	96.6		68.4	91.4	92.5	79.2	74.9	91.4	78.3	74.9	67	83.9	89.1	100	91.4	69.3	82.2	75.4
7. PdeCYP22-2	60.7	72.8	73.8	75.2	73.8	73.3		68.9	69.4	77.8	78.2	68.9	77.4	76.8	54	70.9	69.4	68.4	68	78.3	72.3	79.1
8. PdiCYP18	74.6	90.8	90.2	89.7	100	96.6	73.8		96.5	74.5	77.7	98.8	74.5	76.8	66.3	85	93.7	91.4	95.4	70.2	83.3	80.1
9. PexCYP18-2	74.6	90.2	89.6	89.1	98.8	96	73.8	98.8		75	76.8	96.5	75	76.8	69.1	84.4	93.7	92.5	94.2	69.8	84.5	78.2
10. PflCYP22-2	58.5	73.1	73.6	73.1	78.3	81.6	85.8	78.3	77.8		92	74.5	97.6	91	54.4	67.5	73.6	79.2	73.6	77	67.9	92
11. PfrCYP22-2	61.1	73.9	74.4	73	81.5	79.6	87.2	81.5	80.6	96.7		78.7	91.5	96.7	54.6	68.7	75.8	74.9	78.7	76.4	68.7	96.2
12. PitCYP18-2	74.6	90.8	90.2	89.7	100	96.6	73.8	100	98.8	78.3	81.5		74.5	76.8	66.9	85	94.9	91.4	95.4	70.2	83.3	80.1
13. PnaCYP22-2	58.5	73.1	73.1	73.1	78.8	81.6	85.8	78.8	78.3	99.1	96.7	78.8		90.6	53.9	67	73.1	78.3	73.1	76.5	67	92
14. PnoCYP22-2	60.7	73.5	73.9	72.5	80.6	79.1	86.7	80.6	80.6	96.2	99.1	80.6	96.2		54.2	67.3	75.8	74.9	79.6	75.5	67.8	94.8
15. PocCYP18	70	77.5	78	77.6	79.8	79.3	62.6	78.6	80.3	65.1	65.4	78.6	65.1	65.4		65.2	67.2	67	67.4	54.3	65.4	55.1
16. PoxCYP18	71.7	89	86.7	89.7	89.6	89.1	74.8	89.6	89	72.2	73	89.6	72.6	72.5	76.3		81.7	83.9	83.2	70.7	85.6	70.1
17. ProCYP18-2	73.1	88	88.6	88.6	96	93.7	74.8	96	97.1	77.4	79.6	96	77.8	79.6	77.7	86.9		89.1	91.4	70.7	82.3	77.7
18. PruCYP18-2	71.3	89.7	90.2	89.1	96.6	100	73.3	96.6	96	81.6	79.6	96.6	81.6	79.1	79.3	89.1	93.7		91.4	69.3	82.2	75.4
19. PsoCYP18-2	74	90.2	89.6	89.1	99.4	96	74.3	99.4	98.3	77.8	81	99.4	78.3	80.1	80.3	89	95.4	96		70.2	83.9	79.1
20. PstCYP22-2	61	72.7	71.7	78.5	73.2	75.1	87.4	73.2	72.7	84.4	82	73.2	84.9	81.5	65.4	74.6	74.1	75.1	72.7		74.1	77.8
21. PsuCYP18	71.8	89.1	87.4	95.4	88.5	87.9	76.7	88.5	88.5	72.2	72	88.5	72.2	71.6	77	89.7	88.6	87.9	87.9	77.6		69.2
22. PvuCYP22-2	60.7	74.9	74.4	73.5	82	79.1	86.7	82	81	96.7	99.5	82	97.2	98.6	64	73.5	79.6	79.1	81.5	82.5	73	

Identity

Similarity

Supplementary Table S3d: Percentage identity and similarity of cyclophilins of orthogroup PenCYP04

	1	2	3	4	5	6	7	8	9	10	11	12	13	14	15	16	17	18	19	20	
1. hCYPA		60.6	63.7	62	62.6	60.6	62	63.2	62.6	63.2	60.8	63.7	63.2	63.2	63.2	63.7	62	63.7	59.6	62	**Identity**
2. ParCYP18-1	71.9		85.7	84.5	86.3	61.7	83.9	85.7	84.5	85.7	85.7	85.1	85.1	85.1	83.9	86.3	84.5	85.1	61.7	85.7	
3. PcaCYP17-2	72.6	89.9		92.9	94.6	61.9	94.6	95.2	93.5	97	92.9	96.4	94.6	95.8	97.6	96.4	92.9	98.2	63.1	95.2	
4. PchCYP18-1	73.2	91.1	97.6		92.3	61.9	94	94	97.6	92.9	92.3	94	97	93.5	93.5	93.5	100	93.5	63.7	93.5	
5. PcoCYP18	72.6	91.7	97	96.4		62.1	92.9	93.5	91.7	92.9	94	94.6	92.3	93.5	92.9	95.8	92.3	95.2	64.3	95.8	
6. PdeCYP17-1	73.3	73.7	72	73.8	72.6		61.9	62.5	61.9	61.9	61.3	63.1	61.3	62.5	61.9	62.1	61.9	61.9	61.8	63.1	
7. PdiCYP17-2	73.2	90.5	97.6	97.6	96.4	72		95.8	93.5	92.9	92.9	95.8	93.5	94	94.6	94.6	94	95.2	63.1	94	
8. PexCYP18-1	73.8	90.5	97.6	96.4	96.4	72.6	96.4		93.5	94.6	92.3	97	93.5	94.6	95.2	95.8	94	95.8	64.3	94	
9. PflCYP18-1	73.2	89.9	97	99.4	95.8	73.8	97	95.8		93.5	91.7	93.5	97	92.9	94	92.9	97.6	94	62.5	92.9	
10. PfrCYP18-1	72.6	90.5	98.8	97.6	96.4	72.6	97.6	98.8	97		90.5	94.6	93.5	94	97	95.2	92.9	95.2	61.9	93.5	
11. PgrCYP18-1	73.2	91.7	97	96.4	95.8	73.2	97	95.2	95.8	96.4		93.5	91.1	91.7	92.9	92.3	92.3	94.6	64.3	94	
12. PitCYP18-1	74.4	90.5	98.8	97.6	97	72.6	97.6	98.8	97	98.8	96.4		93.5	95.8	96.4	97	94	97	65.5	95.2	
13. PnaCYP18	73.2	89.9	97.6	98.8	95.8	73.2	96.4	95.2	98.2	96.4	95.8	96.4		92.9	93.5	92.9	97	95.2	62.5	92.3	
14. PnoCYP18-1	73.8	91.1	98.2	98.2	95.8	73.2	97	97	97.6	98.2	95.8	98.2	97		94.6	95.8	93.5	95.2	63.7	95.8	
15. PpoCYP18	72	89.3	98.8	98.2	96.4	72	97.6	97.6	97.6	98.8	97	98.8	96.4	98.2		95.2	93.5	97	62.5	95.2	
16. ProCYP18-1	72.6	91.1	98.2	97	97.6	72.6	97	97.6	96.4	98.2	95.8	98.8	95.8	97.6	98.2		93.5	95.8	64.3	95.2	
17. PruCYP18-1	73.2	91.1	97.6	100	96.4	73.8	97.6	96.4	99.4	97.6	96.4	97.6	98.8	98.2	98.2	97		93.5	63.7	93.5	
18. PsoCYP18-1	73.2	89.9	100	97.6	97	72.6	97.6	97.6	97	98.8	97	98.8	97.6	98.2	98.8	98.2	97.6		63.7	95.8	
19. PstCYP17-1	69.7	73.1	72.6	72	73.2	72.7	72	72.6	71.4	72	73.8	73.2	71.4	71.4	72.6	73.2	72	72.6		64.3	
20. PvuCYP18-1	73.2	90.5	97.6	98.2	96.4	72.6	97.6	96.4	97.6	97.6	97	97.6	96.4	98.2	98.8	97	98.2	97.6	73.2		
	**Similarity**																				

Supplementary Table S3e: Percentage identity and similarity of cyclophilins of orthogroup PenCYP05																										
	1	2	3	4	5	6	7	8	9	10	11	12	13	14	15	16	17	18	19	20	21	22	23	24	25	
1. hCYPA		30.9	30.5	31.8	30.1	31.5	30.6	31.8	30.6	30.1	31.1	30.1	27.7	29.7	30.1	30.1	29.1	31.8	30.1	30.6	31.5	30.1	30.9	33.2	30.6	
2. PanCYP22	43.1		95.7	87.1	90.9	91.9	90.4	86.6	92.8	90.9	91.9	90.9	76.9	90.4	90.1	91.4	76.2	86.6	91.4	91.4	91.9	90.9	83.7	87.1	92.8	
3. ParCYP22	44	98.1		85.2	90	90.9	90.4	88	91.9	90	90.9	90	77.7	89.5	89.2	90.4	73.8	87.6	90.4	90.4	90.9	90	85.6	87.1	92.3	
4. PbrCYP22	44.5	92.8	92.3		86.1	87.1	84.2	86.6	86.6	85.6	86.1	86.6	71.3	85.2	85.4	86.1	77.6	88.5	86.1	85.2	87.1	86.6	84.2	95.2	87.1	
5. PcaCYP22	41.8	94.7	94.7	93.3		95.7	93.3	86.6	96.2	96.6	96.2	99	79.3	96.6	94.3	99.5	75.6	87.1	99.5	95.7	95.7	99	82.3	87.1	95.7	
6. PchCYP22	43.3	95.7	95.7	94.7	96.6		93.3	88	95.7	96.6	98.6	96.6	80.1	95.7	95.7	96.2	76.1	88	96.2	96.2	100	96.6	84.2	88.5	95.7	
7. PcoCYP22-1	43.3	94.7	94.7	92.8	95.7	96.6		85.2	94.2	93.8	93.3	92.8	79.3	92.8	91.5	93.3	75.6	87.1	93.3	94.7	93.3	92.8	82.8	85.2	95.7	
8. PdeCYP22-1	43.1	92.3	93.3	93.3	93.8	94.7	91.9		88	86.6	87.6	87.6	72.1	87.1	86.3	87.1	78.5	89.5	87.1	86.6	88	87.6	87.6	89	86.6	
9. PdiCYP22	42.8	95.2	95.2	92.8	97.6	97.1	97.1	93.3		96.6	96.2	96.2	79.3	96.2	94.3	96.6	77	87.6	96.6	96.6	95.7	96.2	83.7	88	95.7	
10. PexCYP22	42.3	94.3	94.3	92.8	97.6	97.6	96.6	93.3	98.1		96.6	97.6	79.3	97.6	94.8	97.1	75.6	87.1	97.1	96.2	96.6	97.6	82.8	87.1	95.7	
11. PflCYP22-1	42.8	95.7	95.7	93.8	97.1	98.6	96.6	94.3	97.6	97.6		97.1	80.1	96.2	96.2	96.6	75.6	87.1	96.6	96.6	98.6	97.1	84.2	87.6	95.7	**Identity**
12. PfrCYP22-1	41.8	94.3	94.3	93.8	99.5	97.1	95.2	94.3	97.1	98.1	97.6		79.3	97.6	95.3	99.5	75.6	88	99.5	95.7	96.6	100	83.3	88	95.7	
13. PgrCYP26	37	81.3	81.3	79.7	81.7	82.5	82.1	78.9	82.1	81.3	82.5	81.3		78.5	80.9	79.7	61.8	72.5	79.7	79.7	80.1	79.3	69.6	72.5	81.3	
14. PitCYP22	42.3	94.3	94.7	92.8	98.1	97.1	95.7	93.8	98.1	99	97.6	98.6	81.3		94.3	97.1	75.6	86.6	97.1	95.7	95.7	97.6	83.3	86.6	95.2	
15. PnaCYP22-1	42.7	94.8	94.8	93.4	95.7	97.2	95.3	93.4	96.2	96.2	97.6	96.2	83.7	96.2		94.8	75	85.8	94.8	95.3	95.7	95.3	83.5	86.3	93.8	
16. PnoCYP22-1	41.8	94.7	94.7	93.3	100	96.6	95.7	93.8	97.6	97.6	97.1	99.5	81.7	98.1	95.7		75.1	87.6	100	96.2	96.2	99.5	82.8	87.6	96.2	
17. PocCYP22-2	42.9	88.2	87.7	88.2	88.2	90.1	88.2	90.1	89.2	89.2	89.6	88.7	76.4	88.2	90.1	88.2		77.6	75.1	75.6	76.1	75.6	77.1	76.6	74.6	
18. PoxCYP22	45	93.8	93.8	95.7	93.8	95.2	93.8	94.7	93.3	93.8	94.3	94.3	79.7	93.3	93.4	93.8	90.6		87.6	86.1	88	88	86.1	90.9	88	
19. PpoCYP22	41.8	94.7	94.7	93.3	100	96.6	95.7	93.8	97.6	97.6	97.1	99.5	81.7	98.1	95.7	100	88.2	93.8		96.2	96.2	99.5	82.8	87.6	96.2	
20. ProCYP22	42.8	95.7	95.7	92.8	97.1	97.6	97.6	93.3	98.6	97.6	98.1	96.6	82.5	97.6	96.2	97.1	89.2	93.3	97.1		96.2	95.7	83.3	86.6	96.2	
21. PruCYP22	43.3	95.7	95.7	94.7	96.6	100	96.6	94.7	97.1	97.6	98.6	97.1	82.5	97.1	97.2	96.6	90.1	95.2	96.6	97.6		96.6	84.2	88.5	95.7	
22. PsoCYP22	41.8	94.3	94.3	93.8	99.5	97.1	95.2	94.3	97.1	98.1	97.6	100	81.3	98.6	96.2	99.5	88.7	94.3	99.5	96.6	97.1		83.3	88	95.7	
23. PstCYP22-1	44	90.4	90.9	91.9	90.9	93.3	91.4	93.3	91.4	91.9	93.3	91.4	77.6	91.4	92.9	90.9	90.1	92.8	90.9	92.3	93.3	91.4		84.7	82.8	
24. PsuCYP22	45.5	92.8	93.3	98.1	93.3	94.7	92.8	94.3	92.8	92.8	93.8	93.8	79.7	93.3	93.4	93.3	88.7	96.2	93.3	92.8	94.7	93.8	91.4		88	
25. PvuCYP22-1	42.3	95.7	95.7	94.7	97.6	97.6	98.1	92.8	98.1	97.1	97.6	97.1	82.9	97.1	96.2	97.6	88.2	94.7	97.6	98.6	97.6	97.1	90.9	94.7		
	**Similarity**																									

Supplementary Table S3f: Percentage identity and similarity of cyclophilins of orthogroup PenCYP06

	1	2	3	4	5	6	7	8	9	10	11	12	13	14	15	16	17	18	19	20	21	22	23	24	25
1. hCYPA		34.4	37.9	38.1	35.9	45.8	36.9	37.3	36.6	36.2	36.1	36.2	36.2	29.1	29.1	45.8	49.8	48.8	36.6	37	37	36.2	36.8	37.2	37.5
2. PanCYP32	41.7		84.9	70.6	78.9	67.7	77.8	70.1	75.7	77.4	79	79.5	75.7	63.8	63.2	66.3	52.1	62.5	79.5	76.5	78.6	79.2	66.7	72.7	77.8
3. ParCYP29	45.8	87.5		80.1	86.3	73.5	86.2	76.6	85.1	85.8	86.2	87.3	84.5	68.5	68.5	72.7	57.1	67.5	88.1	86.1	86.5	87.7	73.7	78.7	86.4
4. PbrCYP29	46	80.6	89.8		78.5	71.2	78.1	76.9	76.6	77.7	79.9	79.2	77.6	63.3	63.8	69.2	60.5	72.9	79.6	77.5	80.7	79.2	78.1	94.7	77.8
5. PcaCYP29	43.3	85.1	91.9	87.8		74.4	92.2	75.3	92.6	96.3	91.9	98.9	90.8	75.3	72.9	75.2	55.9	67.4	98.1	90	91.9	97.8	72.6	78.9	87.8
6. PchCYP23	55.6	70.1	76.1	74.3	76.7		73.1	71.6	72.8	73.5	76.2	73.9	72.3	60.2	60	94.4	72.2	86.4	74.3	74.3	79.2	73.9	66.2	70.1	74.3
7. PcoCYP29	44.8	83.3	92.2	87.7	95.9	76.5		75.5	89.9	93.3	89.7	93.3	91.5	72.4	71.5	74.6	57.4	66.9	93.3	89.6	88.9	93.7	73.9	77	88.8
8. PdeCYP29	45.6	79.2	86.4	86.8	85.2	76.8	86.9		73.2	75.5	76.4	76.2	73.9	60.2	61.9	70.5	60	67.8	77	74.9	76.6	76.2	72.8	77	76.6
9. PdiCYP30	44.4	82.6	91.4	86.9	95.2	75.7	95.5	84.7		92.5	87.9	93.7	87.5	72.6	69.1	74.3	56.5	67.3	93.7	88.8	88.5	92.5	72.8	76.2	85.8
10. PexCYP29	44	83.7	91.4	86.9	97	76.9	97.4	85.8	96.3		90.8	97.4	90.4	75	71.5	75.7	57	67.7	96.6	90.7	90.4	96.3	72.8	77.8	87.3
11. PflCYP29	44.6	85.1	91.1	87.4	95.2	78.1	95.2	85.9	93.7	94.8		91.5	90.8	73.2	75.6	73.2	56.5	66.9	91.9	89.6	95.9	91.5	73.2	79.3	87.7
12. PfrCYP29	43.7	85.1	92.9	88.1	98.9	76.9	97	86.2	96.3	98.1	95.2		91.5	75	72.9	76.1	57	67.7	99.3	91.1	91.9	98.9	73.5	79.2	88.8
13. PgrCYP29	44.3	84.4	90.8	85.2	94.5	76	96.3	84.9	93.4	94.8	94.5	94.8		70.8	72.1	74.2	57.1	66.5	91.5	87.1	88.9	90.8	73.2	77.2	85.2
14. PitCYP36	35.3	73	72.7	70.6	77.4	61.7	76.6	68.2	76.6	78	76.3	77.2	75.7		73.6	60.2	45.4	53.8	75	72.5	73.2	74.4	57.9	63	69.7
15. PnaCYP37	35.6	71.8	72.1	69.7	75.6	61.8	74.7	68.5	73.8	74.7	77.1	75.6	74.7	83.5		58.2	45.6	52.9	73.2	69.4	74.4	73.2	59.2	62.6	70
16. PnoCYP23	55.7	69.8	75.4	73.2	76.3	97.7	76.9	75.7	76.1	77.2	76.2	77.2	76.4	61.1	60.3		72.9	86.9	76.5	74	74.7	76.1	67.3	68.5	73.9
17. PocCYP22-1	59.9	61.5	67	67.9	63.7	83.6	64.6	68.1	64.6	64.6	65.8	64.6	63.5	51.6	51.8	82.5		78.6	57.4	56.9	56.9	57.4	59.7	60.1	58.2
18. PoxCYP23	57.7	68.1	73.1	76.6	71.9	92.5	72	72.6	71.6	72.4	72.9	72	71.2	57.3	57.4	92	87.8		68	65.8	68.3	67.7	69.7	73.8	66.4
19. PpoCYP29	44	85.4	92.9	88.4	98.5	77.2	97.4	86.6	96.6	97.8	95.5	99.6	95.2	77.4	76.2	77.6	64.9	72.4		91.1	92.2	98.1	74.3	79.6	88.8
20. ProCYP29	45.3	83.7	92.5	87.5	94.1	78.1	95.5	87.2	95.1	95.5	94.4	95.1	93	76	73.5	77.7	65.3	72.8	95.5		88.4	90.3	73.7	77.6	90.6
21. PruCYP29	44.9	84	91.7	87.9	95.2	79.6	94.4	87.5	94	94.8	97.4	95.9	93.7	75.7	76.2	77.7	66.8	73.6	96.3	94.3		91.5	75	79.5	89.1
22. PsoCYP29	43.7	85.4	92.5	88.1	98.1	76.9	97	86.2	95.5	97.4	94.8	99.3	94.1	76.3	75.6	77.2	65.3	72	98.9	94.8	95.1		73.5	79.2	88.8
23. PstCYP29	45.9	79.2	86.8	88.3	85.2	72.2	85.1	85.7	85.8	85.8	84.8	86.2	82.7	68.5	67.9	72.9	68	74.4	86.6	86.8	86.1	86.2		76.4	74.4
24. PsuCYP29	45.5	82.6	89.5	97.7	88.5	74.4	88.1	88	87.3	88.4	88.5	88.8	86.3	70.6	70	73.3	68	76.3	89.2	88	89.1	88.4	88.3		77.5
25. PvuCYP29	45.2	82.3	91.3	87.2	93	78.5	94	86.7	93.3	92.9	92.6	94	91.5	74.2	73.5	78.2	66.7	72.8	94.4	95.1	92.8	94.4	84.2	88	

Identity

Similarity

Supplementary Table S3g: Percentage identity and similarity of cyclophilins of orthogroup PenCYP07

	1	2	3	4	5	6	7	8	9	10	11	12	13	14	15	16	17	18	19	20	21	22	23	24	25
1. hCYPA		24.9	27.5	26.9	27.6	27.3	26.9	26	26.4	27.7	27.9	27.3	27.9	22.5	17.1	17.1	25.4	17.1	22.5	16.9	24.3	17.1	23.7	17.1	25.4
2. PanCYP42	30.1		87	76.8	85	84.7	84.5	80.3	67.9	78.5	82.9	84.7	77.5	73.3	70.6	72.2	15.8	71.9	15	71.2	16.6	72.5	15.8	71.9	16.1
3. ParCYP39	32.7	90.4		80.7	87.6	86.8	87.1	87	71	82	86.6	87.4	81.2	81.2	74.5	76.2	14.4	75.9	13.8	75.1	13.8	76.5	14	75.9	13.5
4. PbrCYP41	31.7	86.5	87.1		81	81.5	81.5	77	70.4	90.2	82.3	82.3	81.3	86.3	67	66	14.5	66.5	14	66.5	14	66.8	14	66.2	14.2
5. PdiCYP41	33.3	89.6	92.2	88.7		96.2	94.6	92.2	72	82.8	93.3	94.4	82.8	76.9	75	78.8	16.7	78.8	16.4	76.6	17.7	79	16.6	78.8	17.2
6. PfrCYP41	33.1	89.9	92.2	89.4	98.7		96	93.3	72	83.3	93.8	95.2	83.4	77.2	75.8	80.6	16.7	80.1	15.6	76.3	16.9	80.1	16.1	80.4	16.9
7. PnaCYP41	32	90.1	92.5	89.2	97.3	98.1		90.9	72.8	83.6	93.3	98.1	83.9	77.4	74.5	78	16.4	78	15.9	75	16.9	78	16.6	78.5	16.7
8. PnoCYP39	32.1	84.9	92.5	84.7	93.5	94.9	93		68.3	79	88.7	90.3	79.4	80	78.9	83.7	16.6	83.1	16.9	79.2	17.2	83.1	15.2	83.9	16.9
9. PocCYP40	33.2	78.4	81.1	82.8	83.9	84.1	84.4	79.8		73.1	72.3	72.8	72.9	65.9	60.2	58.6	13.2	58.9	12.9	59.9	13.5	59.1	12.9	58.6	13.5
10. PoxCYP41	33.1	87	89.5	93.7	90.9	91.7	91.7	87.4	85.8		83.6	83.6	82.8	84.4	69.6	68.3	14	68.8	13.7	68.3	14.2	69.1	13.7	68.5	14.2
11. ProCYP41	33.3	88.8	91.4	89.7	97	97.3	96.2	92.2	83.3	91.7		93	82.6	77.4	74.2	76.6	16.1	76.3	16.1	74.5	16.7	76.3	15.8	76.3	16.4
12. PruCYP41	32.8	90.4	92.7	90	97	97.8	99.5	92.7	84.7	92.2	96		83.9	78.2	74.5	77.4	16.7	77.4	16.1	74.5	17.2	77.4	16.9	77.7	16.9
13. PstCYP40	32.7	86	88.2	90.8	89.5	90.9	91.2	86.3	85.5	92.5	90.6	91.4		76.4	67.6	68.1	13.7	67.8	14.2	66.8	14.5	67.6	14.2	68.1	14.2
14. PsuCYP38	29.4	80.8	88.1	88.9	84.4	85.2	85.2	88.5	77.4	89.2	84.9	85.8	85		74.6	74.6	11.7	75.2	12.5	74.6	13.1	75.5	12	74.6	12
15. PvuCYP33	25.8	74.8	79.8	74.1	78.2	78.5	78.2	81.7	68.7	76.3	78	78.5	74.5	83.1		93.4	10.3	93.7	8.3	93	8.9	94	8.6	93.7	7.9
16. PcaCYP33	25.8	74.8	80.1	72.6	79.8	80.9	79.3	84.2	68.5	75	78.8	79	73.7	81.9	96.4		9.9	98.7	8.6	94	8.9	98.7	8.3	99	8.9
17. PcaCYP7	30.3	16.6	17.5	16.1	18	18	17.7	18.3	16.2	15.9	17.5	17.7	16.1	14.9	13.9	13.9		10.6	89	9.6	91.8	10.6	87.7	8.9	95.9
18. PexCYP33	25.8	74.3	79.5	73.4	79.8	80.4	79.3	83.7	68.5	75.8	78.5	79	73.7	82.8	96.7	98.7	13.9		8.3	94	9.3	99.3	7.9	98.7	8.3
19. PexCYP8	29.1	16.1	16.1	15.8	17.2	17.5	17.2	18.9	15.6	15.9	17.7	17.2	16.4	14.9	12.9	13.2	91.8	11.9		8.3	94.5	7.9	93.2	9.3	91.8
20. PgrCYP33	26.2	75.1	80.1	73.1	79.8	80.1	79.6	83.4	68.5	75.5	78.8	79.3	73.7	82.5	97.4	98.3	14.6	98.3	13.2		8.3	94.7	8.3	94	8.3
21. PgrCYP7	29.7	17.1	16.3	15.8	18.3	18.5	18.3	19.4	16.2	16.4	18	18.3	16.4	15.5	11.9	11.9	94.5	12.6	94.5	12.3		9.3	93.2	9.3	93.2
22. PitCYP33	25.8	74.5	79.8	73.1	80.1	80.6	79.6	83.9	68.2	75.5	78.5	79.3	73.5	82.5	96.7	99	13.9	99.7	12.3	98.7	12.6		7.9	98.7	8.3
23. PitCYP8	29.7	16.9	17.2	15.8	17.7	18	18.3	17.2	15.6	15.9	17.5	18.3	16.4	14	12.3	12.9	89	12.9	93.2	12.9	93.2	12.9		7.9	90.4
24. PsoCYP33	25.8	74.5	79.8	72.8	80.1	80.9	79.6	84.2	68.2	75.3	78.8	79.3	73.7	82.2	96.7	99.3	14.6	99	13.6	98.7	12.3	99.3	12.9		9.3
25. PsoCYP7	30.9	17.1	17.2	16.4	18.5	18.8	18.5	18.3	16.4	16.4	18.3	18.5	16.6	15.2	11.9	14.2	97.3	12.9	94.5	12.6	94.5	12.9	91.8	14.9	

Identity

Similarity

Supplementary Table S3h: Percentage identity and similarity of cyclophilins of orthogroup PenCYP08																							
	1	2	3	4	5	6	7	8	9	10	11	12	13	14	15	16	17	18	19	20	21	22	**Identity**
1. hCYPA		14.3	12.3	12.7	12.9	13.6	14.1	13.4	13.5	13.1	14	12.8	13.6	12.7	14	12.4	13.2	13.6	13	14.4	12.9	13.3	
2. PanCYP52	19.5		74.6	78.1	87	86.9	76.6	77.7	87.3	87.4	86.3	77.1	78.8	87	63.6	77.7	86.2	86.9	87	81.7	80.2	85.8	
3. ParCYP67	16.3	76.4		61	66.4	66.8	58.7	59.7	67.1	66.6	65.9	59.9	60.8	66.1	49.9	61.2	66.3	66.8	66.4	63.6	62.6	66.1	
4. PbrCYP55	18.6	85.3	71		75.5	75.5	67.8	69	75.1	75.9	74.4	69.3	69.4	76.1	61.3	87.9	74.4	75.5	75.5	77.5	94	75	
5. PcaCYP51	19.5	92.8	72	84.5		91.9	80.5	83.6	91.3	99.1	92.7	81.5	82.2	98.2	63	75.3	93.8	91.9	99.1	79.7	76.2	94.3	
6. PchCYP50	19.8	91.2	70.8	83.3	96		81.5	82.3	98.2	92.5	89.8	80.6	84.1	92.1	64.6	74.6	93.3	100	92.1	82.1	76.6	90.3	
7. PdiCYP47	20.6	82.5	63.7	76.7	84.1	85.8		91.1	80.8	80.5	82.4	88.2	85.7	80.4	66.5	66.4	81.8	81.5	80.9	72.4	69.3	81	
8. PexCYP49	19.8	84.7	65.5	77.1	86.9	88.1	92.6		81.8	83.8	84	92.5	89.9	82.8	66	67.1	83.6	82.3	84	73.1	69.9	82.6	
9. PflCYP51	19.6	91.7	71	83.1	96	98.7	84.8	87.5		91.9	89.5	81.3	83.5	91.5	64.1	74.2	92.7	98.2	91.5	81.9	76.8	89.5	
10. PfrCYP51	19.7	93	72.2	84.9	99.8	96.2	84.3	87.2	96.2		92.9	81.7	82.2	97.8	63.2	75.5	93.8	92.5	99.1	80	76.6	94.3	
11. PgrCYP50	20.3	89.7	69.4	81.8	95.1	94.4	86.6	88.5	93.1	95.4		81.1	83.9	91.6	64.4	74.8	91.1	90.1	92.5	79.8	75.9	92.2	
12. PitCYP51	18.8	83.4	66.2	77.7	84.1	85.3	89.2	93.5	84.1	84.3	84.3		87.3	80	64.6	67.8	81.9	80.6	81.9	71.3	69.9	80.7	
13. PnaCYP48	20	83.8	65	77.5	86.3	88.5	90.4	93	88.2	86.5	89.1	89.4		82.3	65.9	68.5	82.3	84.1	82.3	72.8	70.1	82.4	
14. PnoCYP51	19	92.6	72	84.5	99.3	96	85.4	86.9	96	99.6	94.9	85.1	86.3		63.1	75.1	92.9	92.1	97.8	80.2	76.8	93.2	
15. PocCYP49	19.5	75.9	58.1	70.9	74.6	74.6	75.4	77	74.3	75	74.1	75	75.2	74.6		61.6	64.4	64.6	63.1	62.9	61.9	63.5	
16. PoxCYP54	19	84.3	69.8	92.8	84.1	82	75.2	77.7	81.6	84.6	82.3	77.9	77.5	84.1	72.7		73.7	74.6	74.8	77.9	87.9	73.9	
17. ProCYP50	19.4	90.6	70.6	82.2	96.2	97.1	86.3	88.1	96.2	96.5	94.6	84.5	86.7	96	73	82		93.3	93.6	79.2	75.3	92.5	
18. PruCYP50	19.8	91.2	70.8	83.3	96	100	85.8	88.1	98.7	96.2	94.1	85.1	88.5	96	74.6	82	97.1		92.1	82.1	76.6	90.3	
19. PsoCYP51	19.5	92.8	72	84.3	99.3	96	84.5	86.7	96	99.6	95.1	84.9	86	99.1	74.6	83.7	96.2	96		79.5	76.8	94.5	
20. PstCYP51	19	88.4	68.9	85.3	87.8	88.6	80.6	82.1	88.4	88.1	86.6	79.3	82.1	88.5	74.8	84.8	86.6	88.6	87.8		78.5	79.7	
21. PsuCYP54	18.8	87	71.5	96.9	85.1	84.1	77.2	79.1	84.9	85.6	83.5	78.7	78.9	85.1	72.6	93.1	83.9	84.1	85.6	86.6		76.3	
22. PvuCYP51	19.6	92.6	70.6	83.5	96.5	94.6	85	87.7	94.4	96.7	94.4	84.1	86.4	96.5	74.8	83.3	94.9	94.6	96.5	88.4	84.9		
	**Similarity**																						

Supplementary Table S3i: Percentage identity and similarity of cyclophilins of orthogroup PenCYP09

	1	2	3	4	5	6	7	8	9	10	11	12	13	14	15	16	17	18	19	20	21
1. hCYPA		13.7	13.7	12.7	13.2	13.4	13.2	13.2	13.1	13.6	13.2	13.5	12	12.3	11.7	13.2	13.2	13.4	12.9	13.1	12.9
2. PanCYP60	19.1		93.6	74.6	83.2	81.6	80.4	82.2	82.1	82.5	82.2	81.1	76	62.1	71.4	82.5	82.2	81.6	82.7	73.8	75.3
3. ParCYP60	19	96.5		74.6	83.6	81.8	80.9	82.7	82.5	83.2	82.8	81.4	76.1	62.6	71.2	82.7	82.4	81.8	83.2	73.7	75.3
4. PbrCYP60	18.1	84.5	85.2		75	75.8	73.4	75.8	74.5	74.9	74.5	75.4	68.2	61.5	80.6	74.8	74.9	75.8	75	73.4	87.8
5. PcaCYP60	18.6	91.2	91	84.8		93.8	91.9	96	97.4	92.6	95.6	93	89.2	61.9	70.8	98	94.7	93.8	98.3	72.2	73.8
6. PchCYP60	18.4	89	88.8	85	96.9		90.5	93.6	92.7	89.9	93	96.9	84.7	61.6	70.2	93.6	92.7	100	93.6	72.2	74.6
7. PdiCYP60	18.5	88.5	88.3	83.2	94.5	93.6		93.4	89.9	88.8	92.7	89.2	82.7	61.6	69.4	90.7	90.8	90.5	91.8	70.6	71.7
8. PexCYP60	18.2	90.1	90.1	85.2	98	96.7	95.8		93.9	92.1	96.5	92.9	86.4	62.4	70.4	94.7	93.8	93.6	95.8	71.4	74.1
9. PfrCYP60	18.4	90.3	90.1	84.1	98.9	96.7	93.8	97.2		91.4	93.9	92.1	87.9	61.5	70.3	97.2	93	92.7	96.7	70.8	72.9
10. PgrCYP60	18.8	90.6	90.9	84.5	96.1	95.2	93	95.6	95		91.7	89.2	84	62	69.9	91.5	91.4	89.9	92.3	70.7	73.9
11. PitCYP60	18.2	89.7	89.8	84.1	97.1	95.2	94.7	98	96.5	94.9		92.3	86.1	61.9	69.7	94.1	93.4	93	95.4	71.7	73
12. PnaCYP59	18.5	88.4	88.1	84.1	95.8	98	92.9	96	95.8	93.8	94.5		84.1	62.1	71.1	93	91.8	96.9	92.7	72.1	74.9
13. PnoCYP65	17.5	82.5	82.8	78.6	90.4	87.9	86	89.1	89.6	87.4	88.2	87		56.3	64.6	88.7	85.4	84.7	88.9	66	67.3
14. PocCYP62	17.9	76.1	75.2	74.5	75	74.2	75	74.3	74.2	74.7	74.5	75	70.7		59.8	61.6	61.4	62.2	62	59.7	60.9
15. PoxCYP62-2	17.6	82.7	82.5	88.4	82.5	82.5	81.7	82.7	82.2	82.4	81.7	82.9	78.5	73.9		70.4	69.6	70.2	70.6	72.8	80.8
16. PpoCYP60	18.4	90.3	89.9	84.3	98.7	97.1	94	97.4	98.7	95.6	96.3	96.1	90.1	74.5	82.4		93.9	93.6	97.4	71.3	72.8
17. ProCYP60	18.5	90.3	89.6	85.7	97.1	96.3	94.1	96.9	96.7	95.6	96	95.4	88	74	82.5	96.7		92.7	93.9	72.8	73.9
18. PruCYP60	18.4	89	88.8	85	96.9	100	93.6	96.7	96.7	95.2	95.2	98	87.9	75	82.5	97.1	96.3		93.6	72.2	74.6
19. PsoCYP60	18.2	90.3	90.1	84.5	98.9	96.9	94.5	98	98.2	96	96.9	95.6	89.9	75.4	82.2	98.3	96.9	96.9		71.5	73.6
20. PstCYP60	18.9	84.6	84.3	83.9	84.4	83.5	83.3	84.2	83.6	83.5	83.1	82.5	77.1	73.5	82.7	83.6	84.2	83.5	84		74
21. PsuCYP60	17.9	85.2	85.7	93	85.2	85.4	83.5	84.6	84.3	84.4	84.1	84.6	78.1	75.4	86.2	83.7	85.2	85.4	84.3	84.1	

Identity

Similarity

Supplementary Table S3j: Percentage identity and similarity of cyclophilins of orthogroup PenCYP10

	1	2	3	4	5	6	7	8	9	10	11	12	13	14	15	16	17	18	19	20	21	22	23	24
1. hCYPA		14.2	7.2	13.6	13.7	13.9	14	14.7	14.2	14.3	13.7	14.2	13.3	14.3	14	14.9	13.9	13.7	14.2	13.9	14	13.8	14.1	14.7
2. PanCYP62	17.6		46.6	76.5	89.1	88.2	88.7	89.4	89.6	88.7	89.1	87.7	84.2	88.7	88.2	71.2	81.6	89.2	88.9	88.2	89.2	83.4	82.8	88.5
3. ParCYP126	9.1	47.8		38.9	43.4	43	43	43.4	43.5	43	43.4	42.7	44.7	43.2	43	35.6	39.8	43.5	43.3	43	43.5	40.7	40.8	42.7
4. PbrCYP66	16.8	84.8	44.2		77	75.9	76.4	76.1	77.1	76.6	76.9	75.8	72.3	76.4	75.7	66.8	81.7	76.6	75.5	75.9	76.2	78.9	86.9	76.2
5. PcaCYP62	17.1	95.5	46.3	84.7		93.5	92.8	95.5	96.9	93.7	96.5	93.4	90.9	94.1	96.7	69.9	81.1	97	93.5	93.5	99	81.8	83.7	92.3
6. PchCYP62	17.2	94.3	46	83.5	96.2		91.1	93.7	94.4	98.1	92.8	92.1	89.1	97.7	93	70.1	81.1	93.4	93.5	100	93.7	81.3	83	91.1
7. PcoCYP62	17.5	95.1	46.3	83.7	97	95.5		92.3	93.4	91.8	93	92.7	88.7	92.1	92.7	70.9	80.4	93.2	92	91.1	92.8	81.5	83.9	91.4
8. PdiCYP62	17.6	95.3	46.2	83.2	98.1	95.8	96.5		96.7	93.9	94.8	93.9	91.6	94.4	94.6	70.1	80.8	95.3	93.7	93.7	95.5	82	83.4	93
9. PexCYP62	17.5	95.8	46.5	84.5	98.3	96.3	97.4	98.1		94.6	96.2	93.9	92.2	94.9	95.6	69.9	81	96.7	94.6	94.4	97.2	82	84.3	93.2
10. PflCYP61	17.2	95.3	46.3	84	96.7	98.6	96.2	96.3	96.9		93.7	92	89.2	98.1	92.8	70.6	81.1	93.9	93.4	98.1	93.9	81.7	83.2	91.3
11. PfrCYP62	17.1	94.9	46.1	84.3	98.1	95.6	96.9	96.9	97.4	96.5		93.2	90.9	93.9	96.5	69.9	81.5	99.1	93.7	92.8	96.3	81.8	83.9	92.3
12. PgrCYP62	17.7	93.4	45.6	83.4	95.8	94.6	96.5	96	95.8	94.6	95.5		89.1	92.8	93.4	69.9	81.1	93.5	91.6	92.1	93.4	81.7	83.2	91.1
13. PitCYP65	16.6	90.4	48.1	83.2	93	91.1	92.5	92.5	93.2	91.6	92.4	91.2		89.7	90.2	66.9	76.5	91.4	89.4	89.1	90.9	77.3	79.3	88.2
14. PnaCYP62	17.2	94.6	46.2	84.2	96.5	98.4	96.3	96.3	96.7	98.9	96.5	95.1	91.6		93.2	69.9	81.1	94.4	93.9	97.7	94.2	81.8	83.4	91.6
15. PnoCYP62	17.3	94.4	45.8	83.8	98.3	95.8	96.5	96.9	97	96	97.4	95.6	92.1	95.8		69.2	80.1	96.7	93	93	96.5	81.1	83.2	92
16. PocCYP64	18.4	82.4	41.3	77.3	82.6	81.9	82.8	82.1	82.1	82.1	82.1	82.1	79.3	82.1	81.6		72.6	69.9	69.3	70.1	70.1	70.9	72.8	70
17. PoxCYP62-1	17	89.4	43.7	86.5	90.2	88.8	89.4	89.2	90.1	89.2	90.2	89	84.6	89.3	89.2	82.6		81.1	79.9	81.3	80.8	84.4	89.7	79.9
18. PpoCYP62	17.1	95.1	46.2	84.5	98.3	95.8	97	97	97.6	96.7	99.8	95.6	92.5	96.7	97.6	82.1	90.4		94.1	93.4	96.9	81.8	83.9	92.8
19. ProCYP62	17.6	94.4	45.8	83.5	96.2	95.8	96.2	96.3	96.7	96.2	96.5	94.2	91.6	96.2	95.6	82.3	89	96.5		93.5	93.5	81.1	82.7	92
20. PruCYP62	17.2	94.3	46	83.5	96.2	100	95.5	95.8	96.3	98.6	95.6	94.6	91.1	98.4	95.8	81.9	89	95.8	95.8		93.7	81.3	83	91.1
21. PsoCYP62	17.5	95.3	46.3	84.7	99.5	96	97	97.9	98.4	96.5	97.7	95.8	92.9	96.3	98.1	82.6	89.9	97.9	96.2	96		81.5	83.6	92.1
22. PstCYP62	17.4	91.5	44.7	86.3	90.8	90.3	91	90.6	91.5	90.6	91.1	90.8	86.3	90.3	90.6	83	91.1	91.3	90.5	90.3	91.3		84.9	81.1
23. PsuCYP61	17.5	91.5	44.8	89.6	91.4	90.2	90.1	90.1	91.8	90.5	91.4	90	85.8	90.7	90.6	81.9	94.2	91.6	90.1	90.2	91.3	92.7		82.1
24. PvuCYP62	17.6	94.4	45.8	83.8	96	94.4	96	96.5	96.2	94.9	95.6	94.6	91.1	94.9	95.6	82.6	88.5	95.8	95.5	94.4	95.8	90.5	89.5	

Identity

Similarity

Supplementary Table S3k: Percentage identity and similarity of cyclophilins of orthogroup PenCYP11

	1	2	3	4	5	6	7	8	9	10	11	12	13	14	15	16	17	18	19	20	21	22	23	24	25
1. hCYPA		14	14.1	13.1	14.8	13.8	14.6	13.2	14.6	14.5	14.1	14.8	14.6	14.3	14.1	14.6	13.6	13.6	14.8	14.3	13.8	14.6	13.3	13.9	14.6
2. PanCYP69	17.2		97	84.2	91.4	90.4	90.9	87.9	90.3	90.9	90.6	91.2	91.2	90.3	90.6	90.3	76	87.6	91.1	90.3	90.4	91.2	86.2	87.6	91.1
3. ParCYP69	17.4	98.7		84.5	91.2	90.4	90.4	87.8	90.3	91.1	90.6	90.7	91.4	90.3	90.6	90.6	75.8	87.5	90.6	89.8	90.4	90.4	85.9	87.9	90.6
4. PbrCYP72	16.4	90	90.4		84.5	83	83.9	85.4	84.6	84.3	83.6	84.2	83.6	83.7	83.3	83.7	72.8	90	83.9	83.4	83	83.9	84.2	92.2	84.3
5. PcaCYP69	17.9	96.5	96.3	89.6		95.2	95.5	87.9	95.9	97	95.9	98.4	94.6	96.3	95.7	96.5	75.5	87.3	98.1	95.7	95.2	98.4	86.3	87.4	96.3
6. PchCYP69	17.4	95.7	95.7	89.5	98.4		94.3	87.6	94.3	95.9	97.9	95.5	94.9	94.9	98.1	93.9	74.9	86.2	95.1	94.1	100	94.6	85.7	86.5	95.1
7. PcoCYP69	17.9	95.9	95.5	89.2	97.9	97.6		87.4	94.6	95.9	93.9	96	95.9	95.1	93.9	94.1	75.4	86.8	95.9	93.9	94.3	95.5	85.1	86.8	95.9
8. PdeCYP69	16.9	94.6	94.4	90.6	94.1	94	93		87.6	88.2	87.8	88.4	88.1	87.4	87.9	87.3	76.6	88.4	88.4	87.6	87.6	88.2	89.7	89.3	88.4
9. PdiCYP69	17.7	96.5	96	89.3	99	97.9	98.1	93.6		97.4	95.2	96.2	94.1	96.5	94.7	94.9	74.7	87	96	94.4	94.3	95.7	85.9	87.3	95.4
10. PexCYP69	17.5	96.7	96.3	89.5	99.4	98.2	98.4	94	99.7		96.2	97.4	95.4	98.4	96	95.9	74.9	87.1	97.3	96	95.9	97.3	86.3	87.3	96.5
11. PflCYP69	17.4	96	96	89.5	98.9	99.2	97.4	94.4	98.4	98.7		96.2	94.4	95.5	98.4	95.2	74.9	86.8	95.7	94.7	97.9	95.2	86.2	86.8	95.2
12. PfrCYP69	17.9	96.3	96.2	89.3	99.5	98.2	97.9	93.8	99.2	99.5	98.7		95.1	96.5	96	96.5	75.7	87.6	99.4	95.7	95.5	98.2	86.5	87.4	96.2
13. PgrCYP69	17.7	95.7	95.7	89.3	97.8	97.8	98.2	93.3	97.9	98.2	97.3	97.8		94.3	94.7	93.6	75.8	86.8	95.2	93.6	94.9	94.6	87	87.4	95.7
14. PitCYP69	17.5	96.3	96.2	89.3	98.9	97.8	98.1	93.6	99.2	99.5	98.2	99	97.9		95.1	95.7	74.9	86.6	96.3	95.1	94.9	96.3	85.5	87	95.5
15. PnaCYP69	17.4	95.5	95.5	89.2	98.4	98.7	97	93.8	97.9	98.2	99.2	98.2	97.1	97.8		94.4	75.5	87	95.5	94.7	98.1	95.1	86.3	86.8	95.1
16. PnoCYP69	17.9	96.2	96.5	89.6	98.7	97.8	97.4	93.5	98.6	98.9	98.2	98.6	97.3	98.6	97.8		75.5	87.1	96.3	94.4	93.9	96.5	85.4	87.1	94.4
17. PocCYP70	18.2	86.7	86.7	84.2	86.5	86.4	86.1	87.8	85.9	86.1	86.1	86.4	86.5	86.5	86.2	86.2		76.7	75.2	74.9	74.9	75.5	76.3	76.3	75.5
18. PoxCYP69	17.2	93.8	94.1	93.3	92.7	93	92.2	94.6	92.4	92.8	93	92.7	92.7	92.7	92.8	93	88.3		87.3	86.5	86.2	87.3	86.6	94	87.6
19. PpoCYP69	17.9	96.5	96.3	89.3	99.4	98.1	98.1	93.8	99.4	99.7	98.6	99.7	97.9	99.2	98.1	98.7	86.2	92.7		95.2	95.1	98.2	86.6	87.1	96.2
20. ProCYP69	17.5	96.3	95.9	90	98.7	97.8	97.3	94.1	98.2	98.6	98.2	98.6	97.1	98.1	97.9	98.1	86.1	92.7	98.4		94.1	95.9	85.7	86.8	94.3
21. PruCYP69	17.4	95.7	95.7	89.5	98.4	100	97.6	94	97.9	98.2	99.2	98.2	97.8	97.8	98.7	97.8	86.4	93	98.1	97.8		94.6	85.7	86.5	95.1
22. PsoCYP69	17.9	96.8	96.3	89.6	99.4	98.1	97.9	94	99.4	99.7	98.6	99.5	97.8	99.2	98.1	98.7	86.2	92.5	99.7	98.7	98.1		86.8	87.3	96
23. PstCYP69	16.9	92.8	92.8	89.8	92.5	92.7	92.1	94.9	92.4	92.4	92.7	92.2	92.8	92.2	92.5	92.1	87.8	93.5	92.2	92.4	92.7	92.5		87.9	86.2
24. PsuCYP69	17.3	93.3	93.6	93.8	92.4	92.8	92.2	94.8	92.2	92.5	92.7	92.4	92.8	92.5	92.5	92.5	87.5	96.7	92.4	92.8	92.8	92.4	94.1		87.8
25. PvuCYP69	17.7	96	95.7	89.5	98.4	98.4	97.9	94	98.6	98.9	98.2	98.4	98.4	98.4	97.8	97.9	86.1	93	98.6	97.8	98.4	98.4	92.5	92.8	

Identity

Similarity

Supplementary Table S3l: Percentage identity and similarity of cyclophilins of orthogroup PenCYP12

	1	2	3	
1. hCYPA		9.7	9.8	**Identity**
2. PcoCYP121	12.2		94	
3. PgrCYP121	12.1	97.1		
**Similarity**				

Supplementary Table S3m: Percentage identity and similarity of cyclophilins of *Penicillium oxalicum*

	1	2	3	4	5	6	7	8	9	10	11	
1. hCYPA		47.1	53.9	59.2	31.8	48.8	27.7	12.4	11.7	13.9	13.6	**Identity**
2. PoxCYP17	60.6		39.6	44.4	33.2	35.5	22.2	14.8	12.9	14.9	13.3	
3. PoxCYP19	67.6	54.7		49.7	27.4	45.9	27.2	11.5	12	14.8	16	
4. PoxCYP18	71.7	56.1	62.6		28.9	44.7	26.5	13.1	12.9	14.4	13.4	
5. PoxCYP22	45	44.5	41.6	40.7		29.4	21	17.2	13.6	12.4	13	
6. PoxCYP23	57.7	50.2	57.7	54.5	46		28.4	14	12.5	16.6	16.2	
7. PoxCYP41	33.1	28.2	34.1	33.9	30.6	37.1		19.8	20.3	16.9	17.4	
8. PoxCYP54	19	21.1	18.8	19	24.4	21.3	34.4		20.9	17.6	16.6	
9. PoxCYP62-2	17.6	18.2	17.1	18.2	19.2	19.9	31.6	38.1		19	15.6	
10. PoxCYP62-1	17	19.4	19.9	18	18	22.7	28.5	33	34.4		20.7	
11. PoxCYP69	17.2	17.6	21	17.8	19.4	21	27.7	33.5	29.4	38.8		
**Similarity**												

Supplementary Table S3n: Minimum and maximum Percentage similarity of cyclophilins in PPIase orthogroups.					
S. No.	Orthogroups	Minimum percentage similarity		Maximum percentage similarity	
1	PenCYP01	90.7	PocCYP17	100	PvuCYP17
2	PenCYP02	78.2	PocCYP19	100	ProCYP18-3
3	PenCYP03	62.6	PocCYP18	100	PruCYP18-2
4	PenCYP04	71.4	PstCYP17-1	100	PruCYP18-1
5	PenCYP05	76.4	PocCYP22-2	100	PsoCYP22
6	PenCYP06	51.6	PocCYP22-1	99.3	PsoCYP29
7	PenCYP07	78.4	PocCYP40	99.5	PruCYP41
8	PenCYP08	58.1	PocCYP49	100	PruCYP50
9	PenCYP09	70.7	PocCYP62	100	PruCYP60
10	PenCYP10	41.3	PocCYP64	100	PruCYP62
11	PenCYP11	84.2	PocCYP70	100	PruCYP69
12	PenCYP12	97.1	PgrCYP121	97.1	PgrCYP121

Supplementary Table S4: Conservation of active site residues (ASRs) with respect to the human orthologue, hCYPA, hFKBP12, and hPIN1 in the cyclophilin-like domains of cyclophilins, FK506 binding proteins (FKBPs) domains, and Parvulin domains of *Penicillium oxalicum*.

Cyclophilins

Cyclophilins	Orthogroups	Conserved (ASRs)	ARG (R) 55	PHE (F) 60	MET (M) 61	GLN (Q) 63	ALA (A) 101	PHE (F) 113	TRP (W) 121	LEU (L) 122	HIS (H) 126
PoxCYP17	PenCYP01	9	R	F	M	Q	A	F	W	L	H
PoxCYP19	PenCYP02	8	R	F	M	Q	A	F	F	L	H
PoxCYP18	PenCYP03	9	R	F	M	Q	A	F	W	L	H
PoxCYP22	PenCYP05	7	R	F	M	Q	A	F	H	L	S
PoxCYP23	PenCYP06	9	R	F	M	Q	A	F	W	L	H
PoxCYP41	PenCYP07	8	R	F	M	Q	A	F	H	L	H
PoxCYP54	PenCYP08	4	S	F	S	Q	A	L	Y	L	A
PoxCYP62-2	PenCYP09	5	R	F	V	Q	A	F	E	M	C
PoxCYP62-1	PenCYP10	8	R	F	M	Q	A	F	H	L	H
PoxCYP69	PenCYP11	9	R	F	M	Q	A	F	W	L	H

FKBPs

FKBPs	Orthogroups	ASRs	TYR (Y) 27	PHE (F) 37	ASP (D) 38	ARG (R) 43	PHE (F) 47	PHE (F) 49	GLN (Q) 54	GLU (E) 55	ILE (I) 57	TRP (W) 60	TYR (Y) 83	HIS (H) 88	PHE (F) 100
PoxFKBP12-2	PenFKBP1	7	Y	F	D	P	L	V	G	Q	I	W	Y	-	F
PoxFKBP12-1	PenFKBP2	8	Y	F	D	R	L	S	G	R	I	W	Y	F	F
PoxFKBP14	PenFKBP3	9	Y	F	D	R	L	F	G	R	I	W	Y	I	F
PoxFKBP52	PenFKBP4	10	Y	F	D	K	F	F	G	E	I	W	Y	L	F

Parvulins

Parvulins	Orthogroups	Conserved (ASRs)	HIS (H) 59	LYS (K) 63	ARG (R) 68	ARG (R) 69	CYS (C)/ ASP (D)* 113	LEU (L) 122	MET (M) 130	PHE (F) 134	SER (S)/ PHE (F)* 154	HIS (H) 157
PoxPAR14 **(vs hPAR14)**	PenPAR01	6	H	#	#	#	D	L	L	F	F	H
PoxPIN19 **(vs hPIN1)**	PenPIN01	10	H	K	R	R	C	L	M	F	S	H

#: residues absent in hPAR14; *: residues present in hPAR14

Supplementary Table S5a: Percentage identity and similarity of FKBPs of orthogroup PenFKBP01

	1	2	3	4	5	6	7	8	9	10	11	12	13	14	15	16	17	18	19	20	21	22
1. hfkbp12		42.6	42.6	42.6	43.4	43.4	43.4	43.4	43.4	43.4	39	44.3	41.8	43.4	43.4	42.6	43.4	42.6	43.4	42.6	10.2	45.1
2. PanFKBP12-2	61.2		95.9	89.3	90.1	91.7	86	90.1	91.7	91.7	78.5	85.1	91.7	90.9	88.4	90.9	90.1	89.3	91.7	90.9	18.2	89.3
3. ParFKBP12	60.3	99.2		91.7	94.2	94.2	88.4	92.6	94.2	94.2	82.6	89.3	94.2	93.4	92.6	93.4	94.2	91.7	94.2	95	18.8	92.6
4. PbrFKBP12-2	61.2	95.9	96.7		90.9	93.4	87.6	91.7	91.7	93.4	80.2	86.8	91.7	92.6	89.3	98.3	90.9	89.3	93.4	90.9	19.8	90.9
5. PcaFKBP12	60.3	98.3	99.2	96.7		97.5	87.6	96.7	98.3	97.5	88.4	88.4	96.7	96.7	98.3	92.6	100	91.7	97.5	98.3	18.2	92.6
6. PchFKBP12	61.2	98.3	99.2	97.5	99.2		88.4	95.9	97.5	100	86	87.6	95.9	99.2	95.9	95	97.5	94.2	100	95.9	18.8	92.6
7. PcoFKBP12	60.3	94.2	95	94.2	95	95.9		87.6	89.3	88.4	77.7	96.7	87.6	87.6	86	87.6	87.6	88.4	88.4	86.8	17.9	93.4
8. PdiFKBP12	60.3	97.5	98.3	97.5	99.2	98.3	94.2		98.3	95.9	85.1	87.6	96.7	95	95	93.4	96.7	90.1	95.9	95	18.4	91.7
9. PexFKBP12	60.3	98.3	99.2	96.7	100	99.2	95	99.2		97.5	86.8	88.4	98.3	96.7	96.7	93.4	98.3	91.7	97.5	96.7	18.4	93.4
10. PflFKBP12	61.2	98.3	99.2	97.5	99.2	100	95.9	98.3	99.2		86	87.6	95.9	99.2	95.9	95	97.5	94.2	100	95.9	18.8	92.6
11. PfrFKBP11	60.6	88.4	89.3	86.8	90.1	89.3	86	89.3	90.1	89.3		77.7	85.1	85.1	86.8	81	88.4	80.2	86	86.8	15.9	81
12. PgrFKBP12	61.2	94.2	95	95.9	95	95.9	97.5	95.9	95	95.9	85.1		86.8	86.8	86.8	87.6	88.4	87.6	87.6	87.6	17.7	94.2
13. PitFKBP12	59.5	97.5	98.3	95.9	99.2	98.3	94.2	98.3	99.2	98.3	89.3	94.2		95	95	93.4	96.7	91.7	95.9	96.7	18.4	91.7
14. PnaFKBP12	61.2	98.3	99.2	97.5	99.2	100	95.9	98.3	99.2	100	89.3	95.9	98.3		95	94.2	96.7	94.2	99.2	95	18.6	91.7
15. PnoFKBP12	60.3	97.5	98.3	95.9	99.2	98.3	94.2	98.3	99.2	98.3	89.3	94.2	98.3	98.3		90.9	98.3	90.1	95.9	96.7	17.9	90.9
16. PoxFKBP12-2	61.2	96.7	97.5	99.2	97.5	98.3	94.2	98.3	97.5	98.3	87.6	95.9	96.7	98.3	96.7		92.6	90.9	95	92.6	19.8	91.7
17. PpoFKBP12	60.3	98.3	99.2	96.7	100	99.2	95	99.2	100	99.2	90.1	95	99.2	99.2	99.2	97.5		91.7	97.5	98.3	18.2	92.6
18. ProFKBP12	60.3	96.7	97.5	95.9	97.5	98.3	93.4	96.7	97.5	98.3	87.6	93.4	98.3	99.2	96.7	96.7	97.5		94.2	90.9	18.2	90.1
19. PruFKBP12	61.2	98.3	99.2	97.5	99.2	100	95.9	98.3	99.2	100	89.3	95.9	98.3	100	98.3	98.3	99.2	98.3		95.9	18.8	92.6
20. PsoFKBP12	60.3	97.5	98.3	95.9	99.2	98.3	94.2	98.3	99.2	98.3	89.3	94.2	98.3	98.3	98.3	96.7	99.2	96.7	98.3		18.2	91.7
21. PsuFKBP61	13.4	19.9	20.1	20.4	20.1	20.3	19.2	20.3	20.1	20.3	17.9	19.5	19.9	20.3	19.9	20.6	20.1	19.9	20.3	19.9		18
22. PvuFKBP12	61.2	95	95.9	95	95.9	96.7	96.7	95	95.9	96.7	86	97.5	95	96.7	95	95	95.9	95	96.7	95	19.5	

Identity

Similarity

Red color represents lowest and green shows highest percentage value.

Supplementary Table S5b: Percentage identity and similarity of FKBPs of orthogroup PenFKBP02

	1	2	3	4	5	6	7	8	9	10	11	12	13	14	15	16	17	18	19	20	21	22	23	24	25	26
1. hfkbp12		53.9	46.7	45.1	47.1	44.3	45.1	45.9	51.6	45.1	44.3	45.1	31.2	43.4	45.1	44.3	45.1	41.7	47.9	44.3	44.3	45.1	44.3	48	47.1	45.1
2. PanFKBP12-1	71.3		53.7	53.7	59	52	55.3	55.3	55.7	53.7	52	55.3	36.8	52.8	52.8	54.5	52.8	47.9	56.6	52	52.8	55.3	52	60.5	58.2	53.7
3. PanFKBP13	64.8	72.1		93.4	75.4	89.3	90.2	83.6	75.6	89.3	89.3	90.2	60.7	86.1	87.7	89.3	87.7	54.1	73.8	87.7	89.3	90.2	87.7	81.3	75.4	89.3
4. ParFKBP13	63.9	72.1	95.1		76.2	90.2	92.6	86.9	78	90.2	90.2	92.6	62.4	88.5	88.5	93.4	90.2	56.6	73	90.2	90.2	92.6	90.2	79.7	75.4	90.2
5. PbrFKBP12-1	63.6	75.2	84.4	84.4		73.8	76.2	76.2	80.3	73.8	73.8	76.2	52	77	73.8	77	76.2	57	88.4	75.4	73	76.2	75.4	87	94.2	75.4
6. PcaFKBP13-1	63.9	70.5	93.4	92.6	84.4		94.3	91	74.8	98.4	99.2	95.1	67.1	94.3	97.5	93.4	95.1	52.5	70.5	96.7	98.4	94.3	96.7	79.7	72.1	97.5
7. PchFKBP13-1	63.1	73	91.8	92.6	84.4	98.4		88.5	78	95.1	94.3	99.2	64.7	91.8	92.6	99.2	93.4	54.9	73	93.4	93.4	100	93.4	80.5	74.6	94.3
8. PcoFKBP13	63.1	70.5	90.2	91.8	84.4	95.9	94.3		77.2	90.2	91	89.3	64.7	91	92.6	89.3	93.4	55.7	68.9	92.6	90.2	88.5	92.6	80.5	73.8	91
9. PdeFKBP13	64.8	73.8	86.1	86.1	87.7	86.9	86.1	86.1		74.8	74.8	77.2	53.4	78	74.8	78	78	58.2	77.9	77.2	75.6	77.2	77.2	78.2	80.3	76.4
10. PdiFKBP13-1	64.8	72.1	92.6	92.6	83.6	98.4	98.4	95.1	86.9		98.4	95.9	66.5	94.3	96.7	94.3	94.3	52.5	70.5	95.9	97.5	95.1	95.9	80.5	72.1	96.7
11. PexFKBP13-1	63.9	71.3	93.4	92.6	84.4	99.2	99.2	95.9	86.9	99.2		95.1	67.6	94.3	98.4	93.4	95.1	52.5	70.5	97.5	99.2	94.3	97.5	79.7	72.1	98.4
12. PflFKBP13-1	63.9	72.1	91.8	92.6	84.4	98.4	100	94.3	86.9	98.4	99.2		65.3	92.6	93.4	98.4	94.3	54.9	73	94.3	94.3	99.2	94.3	80.5	74.6	95.1
13. PfrFKBP19	44.5	49.7	63.6	64.2	58.4	68.2	68.2	67.1	60.7	68.2	68.8	68.2		65.9	68.2	65.3	67.6	39.3	48	69.4	67.1	64.7	69.4	55.2	51.4	67.6
14. PgrFKBP13-1	63.1	70.5	91	91.8	85.2	96.7	96.7	96.7	87.7	96.7	97.5	96.7	68.2		94.3	92.6	93.4	54.1	70.5	95.1	93.4	91.8	95.1	79.7	75.4	95.9
15. PitFKBP13-1	63.9	71.3	92.6	91.8	83.6	98.4	98.4	96.7	86.1	98.4	99.2	98.4	68.8	96.7		91.8	95.1	54.1	68.9	97.5	97.5	92.6	97.5	79.7	72.1	98.4
16. PnaFKBP13-1	62.3	72.1	91	93.4	85.2	97.5	99.2	95.1	87.7	97.5	98.4	99.2	68.8	97.5	97.5		94.3	55.7	72.1	94.3	92.6	99.2	94.3	79.7	75.4	93.4
17. PnoFKBP13-1	63.1	69.7	91.8	92.6	85.2	97.5	96.7	95.9	88.5	96.7	97.5	96.7	68.2	96.7	96.7	97.5		54.9	69.7	97.5	94.3	93.4	97.5	79.7	74.6	95.1
18. PocFKBP12	58	65.5	65.6	67.2	70.2	64.8	65.6	67.2	69.7	64.8	64.8	65.6	47.4	65.6	65.6	66.4	66.4		55.4	54.1	53.3	54.9	54.1	56.1	57.9	54.1
19. PoxFKBP12-1	64.5	76.9	85.2	83.6	95	82.8	83.6	81.1	84.4	82	82.8	83.6	56.6	82	82	82.8	82.8	68.6		68.9	69.7	73	68.9	79.7	90.9	70.5
20. PpoFKBP13-1	63.1	70.5	91.8	92.6	84.4	98.4	98.4	95.9	87.7	98.4	99.2	98.4	69.4	98.4	98.4	99.2	98.4	65.6	82		96.7	93.4	100	79.7	73.8	97.5
21. ProFKBP13-1	63.9	73	93.4	92.6	83.6	98.4	98.4	95.1	87.7	98.4	99.2	98.4	68.2	96.7	98.4	97.5	96.7	65.6	82	98.4		93.4	96.7	78.9	71.3	97.5
22. PruFKBP13-1	63.1	73	91.8	92.6	84.4	98.4	100	94.3	86.9	98.4	99.2	100	68.2	96.7	98.4	99.2	96.7	65.6	83.6	98.4	98.4		93.4	80.5	74.6	94.3
23. PsoFKBP13-1	63.1	70.5	91.8	92.6	84.4	98.4	98.4	95.9	87.7	98.4	99.2	98.4	69.4	98.4	98.4	99.2	98.4	65.6	82	100	98.4	98.4		79.7	73.8	97.5
24. PstFKBP13	65.9	77.2	90.2	88.6	91.1	89.4	88.6	88.6	87	90.2	89.4	88.6	61.8	87	88.6	87.8	88.6	68.3	88.6	88.6	88.6	88.6	88.6		84.6	81.3
25. PsuFKBP12	63.6	74.4	84.4	83.6	96.7	82.8	82.8	82	86.9	82	82.8	82.8	57.2	83.6	82	83.6	83.6	71.1	96.7	82.8	82	82.8	82.8	87.8		73.8
26. PvuFKBP13-1	63.9	71.3	93.4	92.6	84.4	99.2	99.2	95.9	86.9	99.2	100	99.2	68.8	97.5	99.2	98.4	97.5	64.8	82.8	99.2	99.2	99.2	99.2	89.4	82.8	

Identity

Similarity

Supplementary Table S5c: Percentage identity and similarity of FKBPs of orthogroup PenFKBP03

	1	2	3	4	5	6	7	8	9	10	11	12	13	14	15	16	17	18	19	20	21	22	23	24	25
1. hfkbp12		33.1	33.8	34.8	32.8	32.1	26.2	33.8	34.3	33.6	32.8	32.8	32.8	33.6	32.8	32.8	32.6	34.8	33.6	32.8	32.1	32.8	25.6	35.8	34.3
2. PanFKBP14	49.2		97	84.8	90.2	89.4	69.2	84.8	89.4	88.6	89.4	89.4	90.2	88.6	90.2	90.2	66.4	81.8	87.1	88.6	89.4	89.4	55.3	84.8	90.2
3. ParFKBP14	50	99.2		83.3	90.2	89.4	69.2	84.1	90.2	89.4	89.4	90.9	90.2	89.4	90.2	90.2	67.9	83.3	88.6	90.2	89.4	89.4	54.8	84.8	90.2
4. PbrFKBP14	50.4	92.4	91.7		84.8	81.8	65.1	93.9	84.1	83.3	82.6	84.1	83.3	83.3	82.6	83.3	69.4	92.4	83.3	83.3	81.8	84.1	57.6	95.4	84.8
5. PcaFKBP13-2	48.9	94.7	93.9	91.6		96.9	76.6	85.6	95.4	96.2	96.9	99.2	97.7	96.2	97.7	97.7	70.9	83.3	96.2	96.9	96.9	99.2	54.8	84.8	96.2
6. PchFKBP13-2	48.1	93.9	93.2	90.8	99.2		74.3	82.6	93.9	94.7	98.5	96.2	96.2	94.7	99.2	96.9	68.7	81.8	93.1	95.4	100	96.2	52.8	81.8	93.1
7. PcoFKBP18	39.2	73.1	72.5	70.2	76.6	76		65.7	73.1	73.7	74.3	76	74.9	73.7	74.9	74.9	58.1	64	73.7	74.3	74.3	76	62.3	65.1	73.7
8. PdeFKBP14	50.8	90.9	90.2	96.2	91.6	90.8	70.2		82.6	83.3	83.3	84.8	85.6	82.6	83.3	84.1	71.6	88.5	83.3	84.1	82.6	84.8	58.6	92.4	84.8
9. PdiFKBP13-2	49.6	93.9	94.7	91.6	97.7	96.9	74.9	90.8		96.9	93.9	95.4	94.7	99.2	94.7	93.1	69.4	84.1	94.7	93.1	93.9	94.7	53.8	84.1	93.1
10. PexFKBP13-2	49.6	94.7	94.7	91.6	98.5	97.7	75.4	91.6	98.5		94.7	96.2	95.4	97.7	95.4	93.9	70.1	82.6	96.9	95.4	94.7	95.4	54.8	83.3	95.4
11. PflFKBP13-2	48.9	94.7	93.9	91.6	100	99.2	76.6	91.6	97.7	98.5		96.2	96.2	94.7	99.2	96.9	69.4	82.6	93.1	95.4	98.5	96.2	52.8	82.6	93.1
12. PfrFKBP13	49.6	93.9	94.7	90.8	99.2	98.5	76	90.8	98.5	98.5	99.2		96.9	96.2	96.9	96.9	70.9	82.6	96.9	97.7	96.2	98.5	54.8	84.1	95.4
13. PgrFKBP13-2	48.9	93.9	93.2	91.6	99.2	98.5	76	92.4	96.9	97.7	99.2	98.5		95.4	96.9	95.4	71.6	84.1	93.9	94.7	96.2	96.9	54.8	83.3	93.9
14. PitFKBP13-2	49.6	93.9	94.7	91.6	97.7	96.9	74.9	90.8	100	98.5	97.7	98.5	96.9		95.4	93.9	70.1	83.3	95.4	93.9	94.7	95.4	54.3	83.3	93.9
15. PnaFKBP13-2	48.9	94.7	93.9	91.6	100	99.2	76.6	91.6	97.7	98.5	100	99.2	99.2	97.7		97.7	69.4	82.6	93.9	96.2	99.2	96.9	53.3	82.6	93.9
16. PnoFKBP13-2	49.6	96.2	95.5	90.8	98.5	97.7	75.4	90.8	96.2	96.9	98.5	97.7	97.7	96.2	98.5		68.7	81.8	93.9	96.9	96.9	96.9	53.8	83.3	94.7
17. PocFKBP14	49.3	79.1	79.9	81.3	81.3	80.6	67.8	82.8	81.3	82.1	81.3	82.1	81.3	81.3	81.3	79.9		71.1	69.4	69.4	68.7	71.6	50	71.9	69.4
18. PoxFKBP14	51.1	91.7	90.9	96.9	90.8	90.1	69.6	94.7	90.8	90.8	90.8	90.1	90.8	90.8	90.8	90.1	82.1		81.1	81.1	81.8	82.6	54.5	92.4	82.6
19. PpoFKBP13-2	50.4	93.9	94.7	90.8	97.7	96.9	74.9	90.8	98.5	98.5	97.7	98.5	96.9	98.5	97.7	96.2	82.1	90.1		96.2	93.1	95.4	54.8	83.3	95.4
20. ProFKBP13-2	49.6	94.7	95.5	90.8	98.5	97.7	75.4	90.8	97.7	97.7	98.5	99.2	97.7	97.7	98.5	98.5	82.1	90.1	97.7		95.4	96.2	54.8	83.3	96.2
21. PruFKBP13-2	48.1	93.9	93.2	90.8	99.2	100	76	90.8	96.9	97.7	99.2	98.5	98.5	96.9	99.2	97.7	80.6	90.1	96.9	97.7		96.2	52.8	81.8	93.1
22. PsoFKBP13-2	48.9	94.7	93.9	91.6	100	99.2	76.6	91.6	97.7	98.5	100	99.2	99.2	97.7	100	98.5	81.3	90.8	97.7	98.5	99.2		54.3	84.1	95.4
23. PstFKBP21	36.4	59.6	60.1	61.6	59.1	58.6	69.2	61.1	59.6	59.6	59.6	59.6	58.6	59.6	59.1	58.6	56.6	60.6	59.6	59.6	58.6	59.1		56.1	55.3
24. PsuFKBP14	51.1	93.2	92.4	97.7	92.4	91.6	70.8	96.2	92.4	92.4	92.4	91.6	91.6	92.4	92.4	91.6	82.1	95.4	91.6	91.6	91.6	92.4	60.6		84.8
25. PvuFKBP13-2	49.6	97	96.2	93.1	97.7	96.9	74.9	93.1	96.9	97.7	97.7	96.9	96.9	96.9	97.7	97.7	82.1	92.4	96.9	97.7	96.9	97.7	59.6	93.9	

Identity

Similarity

Supplementary Table S5d: Percentage identity and similarity of FKBPs of orthogroup PenFKBP04

	1	2	3	4	5	6	7	8	9	10	11	12	13	14	15	16	17	18	19	20	21	22	23	24	Identity
1. hfkbp12		10.8	10.8	11.5	11.2	11.2	11.2	10.6	11.1	11.5	11.2	11.2	11.1	11.2	11.2	11.3	11.1	11.2	11.5	11.2	11.1	11.9	11.9	11.2	
2. PanFKBP51	14.9		97.7	80.8	88.2	89	87.8	84.4	86.5	88.3	87.4	87.2	85	88.4	87	66.9	80.4	87.4	88.6	89	87.3	80.8	81.8	86.9	
3. ParFKBP52	14.8	99		81.8	89.6	90.2	88.6	85.1	87.1	89.6	88.8	88.2	85.6	89.6	88.5	67.5	80.8	88.8	89.6	90.2	88.7	81.9	82.8	87.9	
4. PbrFKBP51	15.1	90.8	91		79.8	79.9	80.4	76.5	78.4	80.6	79.3	80.7	77.8	80.3	78.4	65.9	88.3	79.5	81.8	79.9	78.8	82.4	97.1	79.2	
5. PcaFKBP53	15.4	94.9	95.1	88.5		93.7	96.1	92.6	95.8	93.9	97.8	94.3	93.5	94.3	97.1	67.1	79.5	97.8	93.6	93.7	98.8	80.6	80.8	94.7	
6. PchFKBP53	15.4	94.1	94.3	88.7	98		93.3	89.5	91.1	97.8	93.5	92.4	90.4	97.6	92.1	67.8	80.3	93.5	91.2	100	93.1	81.3	80.8	91.9	
7. PcoFKBP53	15.4	94.3	94.5	88.5	99.2	97.5		90.9	94.4	93.9	94.1	96.2	92.3	93.3	93.5	67.7	80.1	94.1	93.4	93.3	95.3	80.8	81.6	95.9	
8. PdiFKBP53	15.1	91.8	91.5	85.7	96	95	95.4		93.2	89.1	92.6	88.8	92.8	89.3	91.8	66	76.1	92.8	90.2	89.9	93.3	77.7	77.1	90.5	
9. PexFKBP54	15.2	93.1	93.3	87.4	97.8	96.4	97.2	97		91.2	95.2	92	94.5	91.2	94.5	66	78	95.2	91.1	91.1	96.4	79.1	79.3	94.2	
10. PflFKBP53	15.4	94	94.2	89.1	97.5	98.8	97.5	94	95.5		91.9	93.7	89.3	96.1	91.7	67.7	81.4	91.9	91.8	97.8	93.1	81.3	81.6	91.5	
11. PfrFKBP53	15.3	94.1	94.3	88.1	99	97.5	98.2	96.6	98.2	96.5		92.4	93.3	93.7	98.2	66.9	77.9	99.6	92.6	93.5	98.6	79.5	79.9	94.3	
12. PgrFKBP53	15.3	93.5	93.7	88.4	97.6	97.1	97.8	94.4	96.4	97.1	96.9		90	92.6	91.8	66.8	80.4	92.4	92.7	92.4	93.6	80.9	81.9	93.2	
13. PitFKBP52	15.5	93.2	93.4	88.4	97.1	95.5	96.7	95.8	96.6	94.9	97.3	94.7		89.9	92.1	66.5	77.2	93.3	90.6	90	93.7	79.6	78.2	92.3	
14. PnaFKBP53	15.3	94.1	94.3	89	98	99	97.5	94.8	96.6	98	98	97.4	95.3		92.5	67.4	80.3	93.7	90.6	97.6	93.5	80.8	81.5	92.1	
15. PnoFKBP53	14.9	93.9	94.1	87.8	98.2	96.9	97.4	96	97.8	96.1	99	96.5	96.3	97.4		66.9	77.3	97.8	92.1	92.1	98	79.3	79.1	93.7	
16. PocFKBP52	15.5	81.6	81.4	81.8	79.9	80.1	79.9	78.1	79.6	80.2	79.6	78.8	81	79.6	79.6		66.4	67.1	68.1	68.1	66.8	67.9	66.6	67.7	
17. PoxFKBP52	15.1	90.1	89.7	93.4	89.5	89.3	88.7	86.5	88.1	89.5	88.5	89.2	87.6	88.8	88.6	80		78	80.7	80.3	78.4	80.8	90.4	79	
18. PpoFKBP53	15.3	94.1	94.3	88.3	99	97.5	98.2	96.8	98.2	96.5	99.6	96.9	97.3	97.8	98.6	79.8	88.3		93	93.5	98.6	79.9	79.7	94.7	
19. ProFKBP51	16	95.4	95.6	90.2	96.9	95.5	96.3	93.8	94.9	95.7	96.3	95.3	94.8	95.3	95.3	81	88.8	96.7		91.2	92.9	81.6	82.2	91.7	
20. PruFKBP53	15.4	94.1	94.3	88.7	98	100	97.5	95	96.4	98.8	97.5	97.1	95.5	99	96.9	80.3	89.3	97.5	95.5		93.1	81.3	80.8	91.9	
21. PsoFKBP53	15.2	93.9	94.1	87.8	99	97.6	98.2	96.8	98.8	96.7	99.2	97.4	97	97.8	98.8	79.3	88.8	99.2	95.9	97.6		79.7	79.7	95.1	
22. PstFKBP51	15.8	91	90.8	91	89.3	89.5	89.5	86.5	88.1	89.1	88.8	89.6	89.4	89.2	88.4	82	89.5	89.2	90.9	89.5	88.6		83.1	79.4	
23. PsuFKBP51	15.5	90.8	91	99.2	88.7	88.9	88.7	85.5	87.9	89.1	88.1	88.6	88	89	87.8	81.2	94.6	87.9	89.5	88.9	88	90.8		79.9	
24. PvuFKBP53	15.3	93.5	93.7	87.8	98.4	96.9	98	96.2	98.2	96.1	98.4	96.7	96.3	97.1	98	79.8	88	98.8	95.7	96.9	98.8	88.8	87.6		

Similarity

Supplementary Table S5e: Percentage identity and similarity of *Penicillium oxalicum* FKBPs

	1	2	3	4	5	
1. hFKBP12		42.6	47.9	34.8	11.1	**Identity**
2. PoxFKBP12-2	61.2		57.4	35.7	11.3	
3. PoxFKBP12-1	64.5	73.6		37.1	10.4	
4. PoxFKBP14	51.1	55.7	52.7		11.4	
5. PoxFKBP52	15.1	15.5	15.3	16.7		
**Similarity**						

Supplementary Table S6a: Comparative analysis of FKBPs of orthogroup PenFKBP01 in different species of *Penicillium*

S. No.	*Penicillium spp.*	Protein name	Protein accession no.	AA	MW (kDa)	pI	FKBP domain	Loc
1	*P. antarcticum*	PanFKBP12-2	OQD86502.1	121	12.96	9.36	116 (2-117)	C
2	*P. arizonense*	ParFKBP12	XP_022493302.1	121	12.89	9.36	116 (2-117)	C
3	*P. brasilianumn*	PbrFKBP12-2	OOQ88851.1	121	12.97	9.36	102 (13-114)	C
4	*P. camemberti*	PcaFKBP12	CRL19576.1	121	12.92	9.36	102 (13-114)	C
5	*P. chrysogenum*	PchFKBP12	KZN93041.1	121	12.90	9.36	102 (13-114)	C
6	*P. coprophilum*	PcoFKBP12	OQE37716.1	121	12.88	9.52	102 (13-114)	C
7	*P. decumbens*	-	-	-	-	-	-	-
8	*P. digitatum*	PdiFKBP12	XP_014536553.1	121	12.98	9.36	116 (2-117)	C
9	*P. expansum*	PexFKBP12	XP_016601872.1	121	12.95	9.36	116 (2-117)	C
10	*P. flavigenum*	PflFKBP12	OQE30842.1	121	12.90	9.36	102 (13-114)	C
11	*P. freii*	PfrFKBP11	KUM63087.1	109	11.53	9.43	96 (13-108)	C
12	*P. griseofulvum*	PgrFKBP12	KXG46819.1	121	12.90	9.52	102 (13-114)	C
13	*P. italicum*	PitFKBP12	KGO67765.1	121	12.98	9.36	116 (2-117)	C
14	*P. nalgiovense*	PnaFKBP12	OQE94790.1	121	12.87	9.36	102 (13-114)	C
15	*P. nordicum*	PnoFKBP12	KOS46094.1	121	12.95	9.36	116 (2-117)	C
16	*P. occitanis*	-	-	-	-	-	-	-
17	*P. oxalicum*	PoxFKBP12-2	EPS29132.1	121	12.97	9.36	116 (2-117)	C
18	*P. polonicum*	PpoFKBP12	OQD63523.1	121	12.92	9.36	102 (13-114)	C
19	*P. roqueforti*	ProFKBP12	CDM30455.1	121	12.89	9.36	102 (13-114)	C
20	*P. rubens*	PruFKBP12	XP_002559257.1	121	12.90	9.36	102 (13-114)	C
21	*P. solitum*	PsoFKBP12	OQD99514.1	121	12.92	9.36	102 (13-114)	C
22	*P. steckii*	-	-	-	-	-	-	-
23	*P. subrubescens*	PsuFKBP61	OKP15160.1	553	61.22	5.6	117 (433-549)	N
24	*P. vulpinum*	PvuFKBP12	OQE05312.1	121	12.89	9.40	102 (13-114)	C

AA: amino acids; C: cytosol; FKBP: FK506 binding domain; kDa: kilodalton; Loc: localization; MW: molecularII8 weight; N: nucleus; pI: isoelectric point. Red color represents lowest and green shows highest value.

Supplementary Table S6b: Comparative analysis of FKBPs of orthogroup PenFKBP02 in different species of *Penicillium*

S. No.	*Penicillium spp.*	Protein name	Protein accession no.	AA	MW (kDa)	pI	FKBP domain	Loc
1. A	*P. antarcticum*	PanFKBP12-1	OQD87264.1	115	12.33	4.95	109 (2- 110)	C
1. B	*P. antarcticum*	PanFKBP13	OQD80475.1	122	13.22	6.41	116 (2- 117)	C
2	*P. arizonense*	ParFKBP13	XP_022490436.1	122	13.11	6.06	116 (2- 117)	C
3	*P. brasilianumn*	PbrFKBP12-1	OOQ88308.1	121	12.87	6.71	116 (2- 117)	C
4	*P. camemberti*	PcaFKBP13-1	CRL23945.1	122	13.11	6.41	116 (2- 117)	C
5	*P. chrysogenum*	PchFKBP13-1	KZN92841.1	122	13.09	6.05	116 (2- 117)	C
6	*P. coprophilum*	PcoFKBP13	OQE39366.1	122	13.15	6.83	116 (2- 117)	C
7	*P. decumbens*	PdeFKBP13	OQD73694.1	122	13.09	6.72	116 (2- 117)	C
8	*P. digitatum*	PdiFKBP13-1	XP_014537689.1	122	13.08	6.05	116 (2- 117)	C
9	*P. expansum*	PexFKBP13-1	XP_016601077.1	122	13.12	6.41	116 (2- 117)	C
10	*P. flavigenum*	PflFKBP13-1	OQE17497.1	122	13.11	6.05	116 (2- 117)	C
11	*P. freii*	PfrFKBP19	KUM60586.1	173	19.03	8.6	116 (53- 168)	C
12	*P. griseofulvum*	PgrFKBP13-1	KXG53875.1	122	13.19	6.83	116 (2- 117)	C
13	*P. italicum*	PitFKBP13-1	KGO70915.1	122	13.17	6.41	116 (2- 117)	C
14	*P. nalgiovense*	PnaFKBP13-1	OQE80745.1	122	13.08	6.05	116 (2- 117)	C
15	*P. nordicum*	PnoFKBP13-1	KOS40134.1	122	13.13	6.41	116 (2- 117)	C
16	*P. occitanis*	PocFKBP12	PCG97418.1	119	12.77	6.57	100 (13- 112)	C
17	*P. oxalicum*	PoxFKBP12-1	EPS30742.1	121	12.93	6.05	116 (2- 117)	C
18	*P. polonicum*	PpoFKBP13-1	OQD64521.1	121	13.16	6.41	116 (2- 117)	C
19	*P. roqueforti*	ProFKBP13-1	CDM26757.1	122	13.15	6.41	116 (2- 117)	C
20	*P. rubens*	PruFKBP13-1	XP_002559045.1	122	13.09	6.05	116 (2- 117)	C
21	*P. solitum*	PsoFKBP13-1	OQD94064.1	122	13.16	6.41	116 (2- 117)	C
22	*P. steckii*	PstFKBP13	OQE16769.1	123	13.17	6.71	118 (2- 119)	C
23	*P. subrubescens*	PsuFKBP12	OKO99241.1	121	12.87	6.06	116 (2- 117)	C
24	*P. vulpinum*	PvuFKBP13-1	OQE11422.1	122	13.15	6.41	116 (2- 117)	C

AA: amino acids; C: cytosol; FKBP: FK506 binding domain; kDa: kilodalton; Loc: localization; MW: molecular weight; pI: isoelectric point. Red color represents lowest and green shows highest value.

Supplementary Table S6c: Comparative analysis of FKBPs of orthogroup PenFKBP03 in different species of *Penicillium*

S. No.	***Penicillium spp.***	**Protein name**	**Protein accession no.**	**AA**	**MW (kDa)**	**pI**	**FKBP domain**	**Loc**
1	*P. antarcticum*	PanFKBP14	OQD87320.1	132	14.11	5.33	93 (30-122)	ER
2	*P. arizonense*	ParFKBP14	XP_022493850.1	132	14.09	5.13	93 (30-122)	ER
3	*P. brasilianumn*	PbrFKBP14	OOQ83031.1	131	14.29	5.38	93 (29-121)	ER
4	*P. camemberti*	PcaFKBP13-2	CRL17162.1	131	13.88	5.35	93 (30-122)	ER
5	*P. chrysogenum*	PchFKBP13-2	KZN92183.1	131	13.87	5.33	93 (30-122)	ER
6	*P. coprophilum*	PcoFKBP18	OQE46659.1	171	18.37	6.97	93 (70-162)	ER
7	*P. decumbens*	PdeFKBP14	OQD77929.1	130	14.15	5.59	92 (29-120)	ER
8	*P. digitatum*	PdiFKBP13-2	XP_014531289.1	131	13.93	4.85	93 (30-122)	ER
9	*P. expansum*	PexFKBP13-2	XP_016593774.1	131	13.83	4.94	93 (30-122)	ER
10	*P. flavigenum*	PflFKBP13-2	OQE30542.1	131	13.87	5.33	93 (30-122)	ER
11	*P. freii*	PfrFKBP13	KUM63881.1	131	13.87	5.12	93 (30-122)	ER
12	*P. griseofulvum*	PgrFKBP13-2	KXG45427.1	131	13.92	5.13	93 (30-122)	ER
13	*P. italicum*	PitFKBP13-2	KGO77266.1	131	13.91	4.83	93 (30-122)	ER
14	*P. nalgiovense*	PnaFKBP13-2	OQE90368.1	131	13.85	5.33	93 (30-122)	ER
15	*P. nordicum*	PnoFKBP13-2	KOS38063.1	131	13.89	5.66	93 (30-122)	ER
16	*P. occitanis*	PocFKBP14	PCG94697.1	134	14.55	5.12	93 (32-124)	ER
17	*P. oxalicum*	PoxFKBP14	EPS30398.1	131	14.34	5.06	93 (29-121)	ER
18	*P. polonicum*	PpoFKBP13-2	OQD66654.1	131	13.88	4.94	93 (30-122)	ER
19	*P. roqueforti*	ProFKBP13-2	CDM33522.1	131	13.87	5.1	93 (30-122)	ER
20	*P. rubens*	PruFKBP13-2	XP_002562332.1	131	13.87	5.33	93 (30-122)	ER
21	*P. solitum*	PsoFKBP13-2	OQE01003.1	131	13.88	5.66	93 (30-122)	ER
22	*P. steckii*	PstFKBP21	OQE28442.1	198	21.57	7.09	93 (96-188)	ER
23	*P. subrubescens*	PsuFKBP14	OKO90935.1	131	14.29	5.38	93 (29-121)	ER
24	*P. vulpinum*	PvuFKBP13-2	OQE07531.1	131	13.91	4.97	93 (30-122)	ER

AA: amino acids; ER: endoplasmic reticulum; FKBP: FK506 binding domain; kDa: kilodalton; Loc: localization; MW: molecular weight; pI: isoelectric point. Red color represents lowest and green shows highest value.

Supplementary Table S6d: Comparative analysis of FKBPs of orthogroup PenFKBP04 in different species of *Penicillium*

S. No.	*Penicillium spp.*	Protein name	Protein accession no.	AA	MW (kDa)	pI	NPL Domain	FKBP Domain	Loc
1	*P. antarcticum*	PanFKBP51	OQD82859.1	478	51.91	4.34	48 (160-208)	188 (291-478)	N
2	*P. arizonense*	ParFKBP52	XP_022485484.1	479	52.05	4.34	48 (160-208)	188 (292-479)	N
3	*P. brasilianumn*	PbrFKBP51	OOQ84335.1	478	51.85	4.35	59 (148-207)	160 (319-478)	N
4	*P. camemberti*	PcaFKBP53	CRL24243.1	487	53.24	4.4	59 (148-207)	170 (318-487)	N
5	*P. chrysogenum*	PchFKBP53	KZN85312.1	488	53.24	4.38	44 (163-207)	104 (385-488)	N
6	*P. coprophilum*	PcoFKBP53	OQE46569.1	487	53.22	4.39	59 (149-208)	129 (359-487)	N
7	*P. decumbens*	-	-	-	-	-	-	-	-
8	*P. digitatum*	PdiFKBP53	XP_014532122.1	497	54.37	4.49	59 (149-208)	129 (369-497)	N
9	*P. expansum*	PexFKBP54	XP_016598739.1	494	54.12	4.44	59 (145-204)	129 (366-494)	N
10	*P. flavigenum*	PflFKBP53	OQE29017.1	486	53.04	4.37	48 (161-209)	205 (282-486)	N
11	*P. freii*	PfrFKBP53	KUM61122.1	489	53.43	4.42	48 (163-211)	129 (361-489)	N
12	*P. griseofulvum*	PgrFKBP53	KXG48330.1	491	53.56	4.33	59 (150-209)	129 (363-491)	N
13	*P. italicum*	PitFKBP52	KGO74787.1	483	52.83	4.42	59 (157-216)	129 (355-483)	N
14	*P. nalgiovense*	PnaFKBP53	OQE84911.1	489	53.25	4.35	59 (149-208)	104 (386-489)	N
15	*P. nordicum*	PnoFKBP53	KOS46420.1	491	53.58	4.43	59 (148-207)	129 (363-491)	N
16	*P. occitanis*	PocFKBP52	PCG97643.1	478	52.10	4.33	45 (168-213)	162 (317-478)	N
17	*P. oxalicum*	PoxFKBP52	EPS34034.1	484	52.72	4.38	48 (163-211)	164 (321-484)	N
18	*P. polonicum*	PpoFKBP53	OQD71048.1	489	53.44	4.42	48 (162-210)	129 (361-489)	N
19	*P. roqueforti*	ProFKBP51	CDM34515.1	475	51.70	4.43	48 (161-209)	190 (286-475)	N
20	*P. rubens*	PruFKBP53	XP_002557951.1	488	53.24	4.38	48 (164-212)	104 (385-488)	N
21	*P. solitum*	PsoFKBP53	OQE03761.1	492	53.85	4.4	59 (148-207)	129 (364-492)	N
22	*P. steckii*	PstFKBP51	OQE16366.1	475	51.56	4.31	59 (149-208)	164 (312-475)	N
23	*P. subrubescens*	PsuFKBP51	OKP02410.1	478	51.87	4.36	59 (148-207)	160 (319-478)	N
24	*P. vulpinum*	PvuFKBP53	OQE05709.1	491	53.64	4.43	48 (164-212)	129 (363-491)	N

AA: amino acids; FKBP: FK506 binding domain; kDa: kilodalton; Loc: localization; MW: molecular weight; N: nucleus; NPL: nucleoplasmin like domain; pI: isoelectric point. Red color represents lowest and green shows highest value.

Supplementary Table S7a: Percentage Identity and similarity of Orthogroup PenPAR01

	1	2	3	4	5	6	7	8	9	10	11	12	13	14	15	16	17	18	19	20	21	22	23	24	25
1. hPAR14		52.2	53	49.6	50	50.8	50.8	43	50.8	50.8	50.8	50.4	51.1	50.8	50	50.4	50	52.6	50.4	50.8	50.8	50.8	51.9	50.8	50.8
2. PanPAR13	67.7		98.4	81.5	91.5	92.2	91.5	67.9	93	93	93	90.6	89.9	93	92.2	89.8	72.6	82.1	90.6	93	92.2	92.2	79.7	80.6	93
3. ParPAR13	68.5	99.2		83	93	93.8	93	69.1	94.6	94.6	94.6	92.2	91.5	94.6	93.8	91.4	74.1	83.6	92.2	94.6	93.8	93.8	81.3	82.1	94.6
4. PbrPAR14	63.8	86.9	87.7		80	81	80.3	62.9	81.8	81.8	81.8	81.7	80	81.8	81	82.4	72.4	93.1	81.7	81.8	81	81	72.1	96.2	81.8
5. PcaPAR13	64.6	93.8	94.6	80.8		97.7	96.9	64.7	98.4	98.4	98.4	96.9	94.6	98.4	97.7	96.1	72.8	80.9	96.9	98.4	97.7	97.7	80	77.9	98.4
6. PchPAR13	65.4	94.6	95.3	86.2	97.7		97.7	65.3	99.2	99.2	99.2	97.7	95.3	99.2	98.4	96.9	72.1	80.9	97.7	99.2	100	98.4	80.8	78.7	99.2
7. PcoPAR13	64.6	95.3	96.1	86.9	98.4	99.2		65.9	98.4	98.4	98.4	96.9	94.6	98.4	97.7	96.1	71.3	80.1	96.9	98.4	97.7	97.7	80	77.9	98.4
8. PdePAR17	**53.7**	72.2	72.8	69.1	69.1	69.8	70.4		65.9	65.9	65.9	66.3	64.1	65.9	65.3	65.7	58.7	64.9	66.3	65.9	65.3	65.3	62	62.1	65.9
9. PdiPAR13	65.4	95.3	96.1	86.9	98.4	99.2	100	70.4		100	100	98.4	96.1	100	99.2	97.7	72.8	81.6	98.4	100	99.2	99.2	81.5	79.4	100
10. PexPAR13	65.4	95.3	96.1	86.9	98.4	99.2	100	70.4	100		100	98.4	96.1	100	99.2	97.7	72.8	81.6	98.4	100	99.2	99.2	81.5	79.4	100
11. PflPAR13	65.4	95.3	96.1	86.9	98.4	99.2	100	70.4	100	100		98.4	96.1	100	99.2	97.7	72.8	81.6	98.4	100	99.2	99.2	81.5	79.4	100
12. PfrPAR13	66.9	93	93.8	84.6	97.7	98.4	99.2	71	99.2	99.2	99.2		94.6	98.4	97.7	99.2	70.7	81.3	100	98.4	97.7	97.7	79.1	78.2	98.4
13. PgrPAR13	**68.5**	93.8	94.6	80.8	96.1	96.9	97.7	69.8	97.7	97.7	97.7	96.9		96.1	95.3	95.3	69.4	80.9	94.6	96.1	95.3	95.3	80.8	79.4	96.1
14. PitPAR13	65.4	95.3	96.1	86.9	98.4	99.2	100	70.4	100	100	100	99.2	97.7		99.2	97.7	72.8	81.6	98.4	100	99.2	99.2	81.5	79.4	100
15. PnaPAR13	65.4	95.3	96.1	86.9	98.4	99.2	100	70.4	100	100	100	99.2	97.7	100		96.9	72.1	80.9	97.7	99.2	98.4	98.4	80.8	78.7	99.2
16. PnoPAR13	66.9	93	93.8	84.6	97.7	98.4	99.2	71	99.2	99.2	99.2	100	96.9	99.2	99.2		69.9	80.5	99.2	97.7	96.9	96.9	78.3	78.9	97.7
17. PocPAR14	63.9	81.2	82	79.7	79.7	78.9	79.7	65.4	79.7	79.7	79.7	76.7	78.2	79.7	79.7	76.7		76.1	70.7	72.8	72.1	72.1	81.3	73.1	72.8
18. PoxPAR14	**66.9**	87.5	88.3	95.4	86.8	86.8	87.6	69.8	87.6	87.6	87.6	83.6	88.4	87.6	87.6	83.6	81.2		81.3	81.6	80.9	80.9	74.8	95.3	81.6
19. PpoPAR13	66.9	93	93.8	84.6	97.7	98.4	99.2	71	99.2	99.2	99.2	100	96.9	99.2	99.2	100	76.7	83.6		98.4	97.7	97.7	79.1	78.2	98.4
20. ProPAR13	65.4	95.3	96.1	86.9	98.4	99.2	100	70.4	100	100	100	99.2	97.7	100	100	99.2	79.7	87.6	99.2		99.2	99.2	81.5	79.4	100
21. PruPAR13	65.4	94.6	95.3	86.2	97.7	100	99.2	69.8	99.2	99.2	99.2	98.4	96.9	99.2	99.2	98.4	78.9	86.8	98.4	99.2		98.4	80.8	78.7	99.2
22. PsoPAR13	65.4	94.6	95.3	86.2	97.7	98.4	99.2	69.8	99.2	99.2	99.2	98.4	96.9	99.2	99.2	98.4	78.9	86.8	98.4	99.2	98.4		80.8	78.7	99.2
23. PstPAR13	65.4	85.2	85.9	80.8	84.5	85.3	86	66.7	86	86	86	85.2	86.8	86	86	85.2	85.7	82	85.2	86	85.3	85.3		72.6	81.5
24. PsuPAR14	64.6	86.8	87.6	98.5	85.3	86	86.8	68.5	86.8	86.8	86.8	83.7	86.8	86.8	86.8	83.7	80.5	96.9	83.7	86.8	86	86	82.2		79.4
25. PvuPAR13	65.4	95.3	96.1	86.9	98.4	99.2	100	70.4	100	100	100	99.2	97.7	100	100	99.2	79.7	87.6	99.2	100	99.2	99.2	86	86.8	

Similarity

Identity

Red color represents lowest and green shows highest percentage value.

Supplementary Table S7b: Percentage Identity and similarity of Orthogroup PenPIN01

	1	2	3	4	5	6	7	8	9	10	11	12	13	14	15	16	17	18	19	20	21
1. hPIN1		49.4	50	49.4	51.4	49.4	50	49.4	50	47.5	48.3	48.9	50	47.9	48.9	50	50	49.4	43.4	44.1	49.7
2. PbrPIN19	65.3		83	84.7	85.8	81.8	83.5	84.7	83.5	80.7	81.3	84.1	83.5	69.2	92.6	82.4	83	84.7	77.7	88.7	83.6
3. PcaPIN19	64.8	91.5		97.2	82.4	97.7	98.9	97.2	99.4	93.9	98.3	95.5	99.4	71.9	83	98.9	98.9	97.2	74.6	74.2	95.5
4. PchPIN19	64.8	92	98.3		84.1	95.5	96	100	97.2	93.4	95.5	98.3	97.7	70.4	83.5	96	97.2	100	74.1	75.8	93.8
5. PdePIN19	65.9	93.2	92.6	93.2		81.3	83	84.1	82.4	80.7	80.7	82.4	83	70.8	84.1	81.8	83	84.1	75.1	78.4	84.7
6. PdiPIN19	64.8	91.5	99.4	97.7	92.6		96.6	95.5	97.2	91.7	96	93.8	97.7	70.3	81.3	96.6	97.2	95.5	72.6	73.2	93.2
7. PexPIN19	64.8	91.5	100	98.3	92.6	99.4		96	98.3	92.8	97.2	94.3	98.3	71.9	83.5	97.7	97.7	96	75.1	74.7	96
8. PflPIN19	64.8	92	98.3	100	93.2	97.7	98.3		97.2	93.4	95.5	98.3	97.7	70.4	83.5	96	97.2	100	74.1	75.8	93.8
9. PfrPIN19	64.8	92	99.4	98.3	92.6	98.9	99.4	98.3		93.4	97.7	95.5	98.9	71.9	83.5	98.3	98.9	97.2	74.1	74.7	94.9
10. PgrPIN20	63.5	89	96.1	95.6	90.6	95.6	96.1	95.6	95.6		93.4	92.8	93.4	72.1	80.2	92.8	92.8	93.4	72.3	74.9	93.4
11. PitPIN19	64.8	91.5	100	98.3	92.6	99.4	100	98.3	99.4	96.1		93.8	97.7	70.4	81.3	97.2	97.2	95.5	73.1	72.7	94.9
12. PnaPIN19	64.4	91.5	96.6	98.3	91.5	96	96.6	98.3	96.6	94.5	96.6		96	69.8	83	94.3	95.5	98.3	73.6	75.8	92.1
13. PnoPIN19	64.8	91.5	100	98.3	92.6	99.4	100	98.3	99.4	96.1	100	96.6		71.4	82.4	98.3	98.3	97.7	74.1	74.7	94.9
14. PocPIN21	59.8	77.8	80.4	79.9	78.4	80.4	80.4	79.9	80.9	81.4	80.4	79.4	80.4		69.9	71.9	71.9	70.4	61.6	69.8	71.6
15. PoxPIN19	**65.7**	97.2	92	92.6	92	92	92	92.6	92.6	89.5	92	92.6	92	78.4		82.4	83	83.5	78.6	83.5	83.6
16. PpoPIN19	64.8	90.3	99.4	97.7	91.5	98.9	99.4	97.7	98.9	95.6	99.4	96	99.4	79.9	90.9		97.7	96	74.1	73.7	94.4
17. ProPIN19	64.8	92	98.9	98.3	93.2	98.9	98.9	98.3	98.9	95	98.9	96.6	98.9	80.4	92	98.3		97.2	73.6	74.2	94.4
18. PruPIN19	64.8	92	98.3	100	93.2	97.7	98.3	100	98.3	95.6	98.3	98.3	98.3	79.9	92.6	97.7	98.3		74.1	75.8	93.8
19. PstPIN21	**57.7**	82.1	83.7	83.2	80.1	83.2	83.7	83.2	83.2	83.2	83.7	82.7	83.7	76.5	82.1	82.7	82.7	83.2		73.3	74.2
20. PsuPIN21	57.7	89.2	82	82.5	84.5	82	82	82.5	82.5	83	82	82.5	82	79.9	86.6	80.9	82.5	82.5	82.7		75.4
21. PvaPIN19	**66.7**	91.5	98.3	97.7	93.8	97.7	98.3	97.7	97.7	96.1	98.3	96	98.3	79.4	91.5	97.7	97.2	97.7	84.2	83	

Similarity

Identity

Supplementary Table S8a: Percentage Identity and similarity of orthogroup PenPTPA01

	1	2	3	4	5	6	7	8	9	10	11	12	13	14	15	16	17	18	19	20	21	22	23	24	25
1. YPA1		33.1	33.5	34.4	31.5	31.9	32.2	35.4	31.9	32.6	32.7	31.8	32.1	32.4	32.6	30.7	34.3	32.4	31.6	31.6	31.9	31.3	33.1	32.8	31.5
2. PanPTPA52	46.3		91.5	71.9	78.3	77.3	76.3	73.1	75.9	77.3	77.9	78.1	76.5	75.9	77.8	69.1	60	70.2	76.9	77.5	77.3	77.9	74.9	74.6	77.5
3. ParPTPA52	48.1	94.8		70.9	77.7	76.3	76.1	73.1	74.8	76.9	76.7	76.9	75.7	75.1	77.1	68.5	60	70.1	76.5	77.5	76.3	77.3	73.3	74	77
4. PbrPTPA51	46.7	83.1	82.2		73.1	72.8	72.9	77.3	71.9	72.5	73.1	73.5	72.4	72.5	73.1	64.8	58.4	81.3	73.3	72.8	72.8	73.1	78.4	91	72.5
5. PcaPTPA52	44.5	87.6	86.6	83.7		90.6	87.5	75.1	90.9	92.8	91	97.3	89.9	91.3	90.6	85.8	60.2	70.8	96.3	89.5	90.6	97.9	75.2	74.5	89.3
6. PchPTPA52	45.4	86.4	85.4	82.8	92.8		85.4	73.2	86.2	87.3	97.5	90.1	86.4	86.9	96.9	80.1	60	69.7	89.1	87.1	100	89.7	73.8	73.6	86.2
7. PcoPTPA51	46.4	85.5	85.5	83.5	92	89.1		71.8	87.4	85.2	85.8	87.5	91.4	84.4	85.7	77.8	60.5	69.5	87.1	83.8	85.4	86.9	72.2	74.5	85.4
8. PdePTPA51	48.2	83.5	82.2	86.9	83.3	81.3	83		72.7	72.7	73.2	73.8	73.1	72.9	72.4	66.5	61.6	75.2	74	74.9	73.2	75.1	78.5	78.1	74.1
9. PdiPTPA51	45.6	85.1	83.7	82.9	94.2	89.5	91.8	83.5		89.1	86.4	90.3	85.2	88	86.3	80.2	60.3	68.9	89.3	85.2	86.2	90.1	72.9	73.1	85.4
10. PexPTPA52	47	86.8	85.6	83.9	96.5	91.6	91.1	81.9	93.6		88.1	92.6	87.9	91.5	87.7	81	59.9	69.4	92	86.4	87.3	92.6	73.6	73.5	86.4
11. PflPTPA52	46.2	86.4	85.6	83.6	93.8	98.4	90.1	81.7	90.6	92.8		90.8	87.1	87.3	97.3	80.5	60.9	69.9	89.3	87.1	97.5	90.1	74.2	74.1	86.7
12. PfrPTPA52	45.6	87	86.4	83.9	98.4	93	91.8	82.9	94	96.5	94.3		89.1	90.5	90.6	85.4	60.9	70.3	97.5	88.5	90.1	96.5	74.7	74.5	88.5
13. PgrPTPA52	44.6	85.6	85	81.7	92.6	89.9	93.6	81.7	88.9	91.8	91	92		87.1	87.1	78.4	58.1	69.7	88.5	85.2	86.4	89.9	73.3	74.3	87.5
14. PitPTPA52	46	86.8	85.8	84.1	95.5	91.2	89.7	81.9	92.4	95.7	92.4	95.3	91		86.9	79.8	60	71	90.3	86	86.9	91.1	74.5	74.7	87.3
15. PnaPTPA52	46.3	86.3	85.9	82.8	92.8	97.5	89.8	81.1	89.8	91.8	98	93.2	90.6	91.4		79.7	60.5	69.5	89.1	86.5	96.9	89.8	73.6	74.3	86.5
16. PnoPTPA46-1	46.9	77.7	76.7	73.3	86.6	81.7	82.4	75.1	84.6	84.5	82.5	86.4	80.5	83.7	81.4		55.4	62.8	85.2	80	80.1	84.9	67	66.1	78.2
17. PocPTPA49	**49.2**	73.3	72.3	71.3	72	70.6	71.2	73.6	71.7	70.9	71.5	72.6	71	70.7	70.7	69.3		57.9	61	60	60	60.4	62	58.5	59.3
18. PoxPTPA51	45.4	82.2	82	88.8	82.3	81.1	82	84.5	81.8	81.9	81.7	82.1	81.5	82.1	81.1	72.6	70.5		69.7	70.5	70.1	70.8	75.3	82.3	69.4
19. PpoPTPA52	45.2	86.6	85.6	83.1	97.9	92.2	91.3	82.9	93.4	96.1	93	98.4	91.6	94.8	92	86.2	72.6	81		88.3	89.1	95.3	74.1	73.7	87.9
20. ProPTPA52	46.1	88	86.6	83.6	93.4	91	89.4	83.6	90.9	92.2	91.8	92.8	89.3	91.8	91	83.2	71.2	82.6	92.4		87.1	89.3	74	74.2	85
21. PruPTPA52	45.4	86.4	85.4	82.8	92.8	100	89.1	81.5	89.5	91.6	98.4	93	89.9	91.2	97.5	81.7	70.6	81.3	92.2	91		89.7	73.8	73.6	86.2
22. PsoPTPA52	44.5	87.2	86	84.1	99	92.8	91.5	83.9	94.2	96.9	93.8	98.1	93	95.9	92.8	86.4	72.4	82.3	97.7	93.2	92.8		75.8	74.5	89.5
23. PstPTPA52	45.5	85.5	84.3	89.6	84.5	83.2	83	87.4	83	83.7	83.8	84.1	83	84.5	83	75.4	72.7	85.7	83.9	84.7	83.2	85.2		79.6	74.6
24. PsuPTPA51	46.1	83.5	83.5	95	84.9	83.4	83.9	86.8	83.5	84.7	84.4	84.5	83.4	85.2	83.8	74.9	72.2	89.1	84.1	84.6	83.4	84.9	89.2		73.4
25. PvuPTPA52	**44.3**	87.2	86.2	83.5	93.4	90.3	91.1	83.7	90.3	92.6	91.4	93	92	92.6	90.6	81.6	72	81	93.2	90.3	90.3	93.4	84.5	83.9	

Similarity

Identity

Red color represents lowest and green shows highest percentage value.

Supplementary Table S8b: Percentage Identity and similarity of orthogroup PenPTPA02

	1	2	3	4	5	6	7	8	9	10	11	12	13	14	15	16	17	18	19	20	21	22	23
1. YPA2		40.8	41.8	39.3	39.8	39.6	39.7	39.1	40.2	40	39.6	40	40.2	40.2	40.5	35.3	38.1	40	39.8	39.6	39.4	38.6	40.2
2. PanPTPA46	56.4		96.1	78.4	87.8	86.9	87.9	79.7	86.2	89	86.4	88.3	88.8	86.9	88.1	66.8	77.3	88.3	85.2	86.9	87.8	78.1	88.3
3. ParPTPA46	56.4	97.3		77.7	88.3	87.8	87.9	80	86.6	89.3	87.6	88.8	89.5	87.1	88.1	67.1	76.2	89	85.2	87.8	88.3	77.4	88.5
4. PbrPTPA46	57	88.8	88.1		77.5	77.8	76.9	81.7	76.6	77.8	77.1	77.5	78.7	77.3	78	68.4	86.5	77.5	76.8	77.8	77.1	91.4	77.3
5. PcaPTPA46	55.4	91.6	92.4	86.9		93.3	94.8	77.6	91.9	96.2	92.4	98.1	94.5	93.8	95	65.9	75.3	97.9	92.4	93.3	97.9	76.1	93.8
6. PchPTPA46	56.1	92.1	92.8	87.4	95.2		92.6	78.6	91.2	93.6	98.3	93.3	94.3	92.1	91.6	66	75.4	93.6	89.8	100	93.1	76.4	91.6
7. PcoPTPA46	55	92.1	92.1	86.7	96.7	95.7		77.1	91.7	95.2	91.7	95.2	95.2	93.3	94.3	64.8	75.8	95.2	90.7	92.6	95	75.5	94.3
8. PdePTPA46	56.9	89.9	90.8	90.3	87.1	87.8	86.9		76.7	77.6	77.9	77.8	79	76.7	77.6	67.9	80	78.3	76.8	78.6	77.6	79.7	77.9
9. PdiPTPA46	54.9	92.1	92.1	88.1	95.5	95.9	96.4	87.4		94.5	90.9	92.8	92.8	92.8	91.9	65.2	72.7	92.6	89	91.2	91.9	74.6	91.6
10. PexPTPA46	55.4	93.6	93.6	87.9	97.1	95.9	97.9	88.1	97.6		92.6	96.7	96.4	96.2	94.7	66.6	74.6	96.7	93.3	93.6	96.2	75.9	95.9
11. PflPTPA46	55.8	91.4	92.6	86.7	95	99.3	95.5	87.4	95.9	95.7		92.4	93.3	90.9	91.6	66.4	74	92.6	88.8	98.3	92.1	75.7	90.7
12. PfrPTPA46	55.1	92.6	93.3	86.9	98.6	95.7	97.9	87.6	96.4	98.1	95.5		95.2	94.5	95.9	65.9	74.8	98.8	92.1	93.3	97.9	75.9	94.5
13. PgrPTPA46	55.6	92.4	92.8	88.1	95.9	96.2	97.6	88.1	96.4	98.1	95.9	97.1		94.5	93.8	66.4	75.5	95.2	91.4	94.3	95	76.4	95.9
14. PitPTPA46	55.1	92.6	92.4	88.4	95.9	95.5	96.9	87.8	96.4	97.6	95	97.1	96.9		92.4	64.8	74.1	94	91.4	92.1	93.8	75.7	93.3
15. PnoPTPA46-2	54.9	92.6	92.8	86.9	96.9	95.2	97.9	86.4	96.4	97.6	95	97.9	96.9	96.4		66.4	74.6	95.7	90.5	91.6	95.5	76.4	92.8
16. PocPTPA47	**53.1**	79.2	78.3	80.7	77.8	78.1	79	80.4	77.6	78.1	78.3	77.6	78.3	78.3	77.1		66.8	65.9	65.1	66	64.9	68.4	66.8
17. PoxPTPA46	55.5	86.7	86.7	91.5	85.8	87	85.5	89.6	85.5	85.3	86	85.5	86.3	87	85.3	80.2		75.3	74.9	75.6	74.6	86	75.5
18. PpoPTPA46	55.4	92.6	93.6	86.5	98.3	95.5	97.9	87.6	96.2	98.1	95.2	99.3	97.1	96.7	97.6	77.4	85.5		92.1	93.6	98.1	75.9	94.5
19. ProPTPA46	55.4	92.3	92.3	86.9	95.9	95	96	87.5	95.5	96.7	94.7	96.4	95.5	95.7	95.7	78.8	86.5	96.4		89.8	92.1	74.9	90.7
20. PruPTPA46	56.1	92.1	92.8	87.4	95.2	100	95.7	87.8	95.9	95.9	99.3	95.7	96.2	95.5	95.2	78.1	86.7	95.5	95		93.1	76.4	91.6
21. PsoPTPA46	54.9	92.4	93.1	86.9	98.6	95.7	97.9	87.6	96.2	97.9	95.5	99	97.1	96.4	97.6	77.6	85.8	99.3	96.7	95.7		74.9	94
22. PsuPTPA47	**57.4**	88.3	88.1	95.7	87.1	87.4	86.2	90.2	88.3	87.9	86.7	86.9	87.4	88.6	86.7	81.4	93.4	86.4	87.1	87.4	86.9		76.1
23. PvuPTPA46	56.1	92.4	92.6	87.2	95.7	94.7	97.1	87.1	96.2	97.9	94.5	96.9	97.4	96.7	96.7	78.3	86.7	96.9	95.2	94.7	96.7	87.6	

Identity

Similarity

Supplementary Table S9: Percentage identity and similarity of FKBPs of orthogroup PenFKBP03 with hFKBP13

	1	2	3	4	5	6	7	8	9	10	11	12	13	14	15	16	17	18	19	20	21	22	23	24	25
1. hFKBP13		53.1	53.8	52.4	52.8	51.4	42.8	52.1	54.2	53.5	51.4	52.8	52.8	53.5	51.4	52.1	49.7	52.4	53.5	51.4	51.4	52.8	37.7	53.1	53.5
2. PanFKBP14	66.9		97	84.8	90.2	89.4	69.2	84.8	89.4	88.6	89.4	89.4	90.2	88.6	90.2	90.2	66.4	81.8	87.1	88.6	89.4	89.4	55.3	84.8	90.2
3. ParFKBP14	66.9	99.2		83.3	90.2	89.4	69.2	84.1	90.2	89.4	89.4	90.9	90.2	89.4	90.2	90.2	67.9	83.3	88.6	90.2	89.4	89.4	54.8	84.8	90.2
4. PbrFKBP14	65.5	92.4	91.7		84.8	81.8	65.1	93.9	84.1	83.3	82.6	84.1	83.3	83.3	82.6	83.3	69.4	92.4	83.3	83.3	81.8	84.1	57.6	95.4	84.8
5. PcaFKBP13-2	66.2	94.7	93.9	91.6		96.9	76.6	85.6	95.4	96.2	96.9	99.2	97.7	96.2	97.7	97.7	70.9	83.3	96.2	96.9	96.9	99.2	54.8	84.8	96.2
6. PchFKBP13-2	65.5	93.9	93.2	90.8	99.2		74.3	82.6	93.9	94.7	98.5	96.2	96.2	94.7	99.2	96.9	68.7	81.8	93.1	95.4	100	96.2	52.8	81.8	93.1
7. PcoFKBP18	55.6	73.1	72.5	70.2	76.6	76		65.7	73.1	73.7	74.3	76	74.9	73.7	74.9	74.9	58.1	64	73.7	74.3	74.3	76	62.3	65.1	73.7
8. PdeFKBP14	64.8	90.9	90.2	96.2	91.6	90.8	70.2		82.6	83.3	83.3	84.8	85.6	82.6	83.3	84.1	71.6	88.5	83.3	84.1	82.6	84.8	58.6	92.4	84.8
9. PdiFKBP13-2	66.9	93.9	94.7	91.6	97.7	96.9	74.9	90.8		96.9	93.9	95.4	94.7	99.2	94.7	93.1	69.4	84.1	94.7	93.1	93.9	94.7	53.8	84.1	93.1
10. PexFKBP13-2	66.9	94.7	94.7	91.6	98.5	97.7	75.4	91.6	98.5		94.7	96.2	95.4	97.7	95.4	93.9	70.1	82.6	96.9	95.4	94.7	95.4	54.8	83.3	95.4
11. PflFKBP13-2	64.8	94.7	93.9	91.6	100	99.2	76.6	91.6	97.7	98.5		96.2	96.2	94.7	99.2	96.9	69.4	82.6	93.1	95.4	98.5	96.2	52.8	82.6	93.1
12. PfrFKBP13	66.2	93.9	94.7	90.8	99.2	98.5	76	90.8	98.5	98.5	99.2		96.9	96.2	96.9	96.9	70.9	82.6	96.9	97.7	96.2	98.5	54.8	84.1	95.4
13. PgrFKBP13-2	66.2	93.9	93.2	91.6	99.2	98.5	76	92.4	96.9	97.7	99.2	98.5		95.4	96.9	95.4	71.6	84.1	93.9	94.7	96.2	96.9	54.8	83.3	93.9
14. PitFKBP13-2	66.9	93.9	94.7	91.6	97.7	96.9	74.9	90.8	100	98.5	97.7	98.5	96.9		95.4	93.9	70.1	83.3	95.4	93.9	94.7	95.4	54.3	83.3	93.9
15. PnaFKBP13-2	64.8	94.7	93.9	91.6	100	99.2	76.6	91.6	97.7	98.5	100	99.2	99.2	97.7		97.7	69.4	82.6	93.9	96.2	99.2	96.9	53.3	82.6	93.9
16. PnoFKBP13-2	65.5	96.2	95.5	90.8	98.5	97.7	75.4	90.8	96.2	96.9	98.5	97.7	97.7	96.2	98.5		68.7	81.8	93.9	96.9	96.9	96.9	53.8	83.3	94.7
17. PocFKBP14	62.7	79.1	79.9	81.3	81.3	80.6	67.8	82.8	81.3	82.1	81.3	82.1	81.3	81.3	81.3	79.9		71.1	69.4	69.4	68.7	71.6	50	71.9	69.4
18. PoxFKBP14	66.9	91.7	90.9	96.9	90.8	90.1	69.6	94.7	90.8	90.8	90.8	90.1	90.8	90.8	90.8	90.1	82.1		81.1	81.1	81.8	82.6	54.5	92.4	82.6
19. PpoFKBP13-2	66.2	93.9	94.7	90.8	97.7	96.9	74.9	90.8	98.5	98.5	97.7	98.5	96.9	98.5	97.7	96.2	82.1	90.1		96.2	93.1	95.4	54.8	83.3	95.4
20. ProFKBP13-2	64.8	94.7	95.5	90.8	98.5	97.7	75.4	90.8	97.7	97.7	98.5	99.2	97.7	97.7	98.5	98.5	82.1	90.1	97.7		95.4	96.2	54.8	83.3	96.2
21. PruFKBP13-2	65.5	93.9	93.2	90.8	99.2	100	76	90.8	96.9	97.7	99.2	98.5	98.5	96.9	99.2	97.7	80.6	90.1	96.9	97.7		96.2	52.8	81.8	93.1
22. PsoFKBP13-2	65.5	94.7	93.9	91.6	100	99.2	76.6	91.6	97.7	98.5	100	99.2	99.2	97.7	100	98.5	81.3	90.8	97.7	98.5	99.2		54.3	84.1	95.4
23. PstFKBP21	49.5	59.6	60.1	61.6	59.1	58.6	69.2	61.1	59.6	59.6	59.6	59.6	58.6	59.6	59.1	58.6	56.6	60.6	59.6	59.6	58.6	59.1		56.1	55.3
24. PsuFKBP14	66.2	93.2	92.4	97.7	92.4	91.6	70.8	96.2	92.4	92.4	92.4	91.6	91.6	92.4	92.4	91.6	82.1	95.4	91.6	91.6	91.6	92.4	60.6		84.8
25. PvuFKBP13-2	67.6	97	96.2	93.1	97.7	96.9	74.9	93.1	96.9	97.7	97.7	96.9	96.9	96.9	97.7	97.7	82.1	92.4	96.9	97.7	96.9	97.7	59.6	93.9	

Identity

Similarity

Supplementary Table S10: Percentage identity and similarity of FKBPs of orthogroup PenFKBP04 with Fpr4

	1	2	3	4	5	6	7	8	9	10	11	12	13	14	15	16	17	18	19	20	21	22	23	24
1. Fpr4		39.9	38.9	39	39.4	38.4	39.4	39.4	38.6	37.5	39.3	37.9	40.3	38.6	38.8	38.5	38.4	39.4	39.3	38.8	39.8	38.6	39.8	39
2. PcoFKBP53	55		94.1	87.8	93.9	88.6	93.3	67.7	80.1	81.6	95.9	80.4	90.9	94.1	93.3	93.5	93.4	93.3	80.8	96.1	94.4	92.3	96.2	95.3
3. PpoFKBP53	54.8	98.2		87.4	92.3	88.8	93.5	67.1	78	79.7	94.7	79.5	92.8	99.6	93.7	97.8	93	93.5	79.9	97.8	95.2	93.3	92.4	98.6
4. PanFKBP51	54.8	94.3	94.1		88.3	97.7	89	66.9	80.4	81.8	86.9	80.8	84.4	87.4	88.4	87	88.6	89	80.8	88.2	86.5	85	87.2	87.3
5. PflFKBP53	54.1	97.5	96.5	94		89.6	97.8	67.7	81.4	81.6	91.5	80.6	88.7	91.9	96.1	91.7	91.8	97.8	81.3	93.9	91.2	89.3	93.7	93.1
6. ParFKBP52	54.3	94.5	94.3	99	94.2		90.2	67.5	80.8	82.8	87.9	81.8	85.1	88.8	89.6	88.5	89.6	90.2	81.9	89.6	87.1	85.6	88.2	88.7
7. PchFKBP53	54.1	97.5	97.5	94.1	98.8	94.3		67.8	80.3	80.8	91.9	79.9	89.5	93.5	97.6	92.1	91.2	100	81.3	93.7	91.1	90.4	92.4	93.1
8. PocFKBP52	53.8	79.9	79.8	81.6	80.2	81.4	80.1		66.4	66.6	67.7	65.9	66	66.9	67.7	66.9	68.1	68.1	67.9	67.3	65.6	66.5	66.8	66.8
9. PoxFKBP52	52.5	88.7	88.3	90.1	89.5	89.7	89.3	80		90.4	79	88.3	76.1	77.9	80.3	77.3	80.7	80.3	80.8	79.5	78	77.2	80.4	78.4
10. PsuFKBP51	53.3	88.7	87.9	90.8	89.1	91	88.9	81.2	94.6		79.9	97.1	77.1	79.9	81.5	79.1	82.2	80.8	82.6	80.8	79.3	78.2	81.9	79.7
11. PvuFKBP53	54.8	98	98.8	93.5	96.1	93.7	96.9	79.8	88	87.6		79.2	90.5	94.3	92.1	93.7	91.7	91.9	79.4	94.7	94.2	92.3	93.2	95.1
12. PbrFKBP51	53.1	88.5	88.3	90.8	89.1	91	88.7	81.8	93.4	99.2	87.8		76.5	79.3	80.3	78.4	81.8	79.9	82.4	79.8	78.4	77.8	80.7	78.8
13. PdiFKBP53	53.7	95.4	96.8	91.8	94	91.5	95	78.1	86.5	85.5	96.2	85.7		92.6	89.3	91.8	90.2	89.9	77.7	92.6	93.2	92.8	88.8	93.3
14. PfrFKBP53	55.2	98.2	99.6	94.1	96.5	94.3	97.5	79.6	88.5	88.1	98.4	88.1	96.6		93.7	98.2	92.6	93.5	79.5	97.8	95.2	93.3	92.4	98.6
15. PnaFKBP53	53.6	97.5	97.8	94.1	98	94.3	99	79.8	88.8	89	97.1	89	94.8	98		92.5	90.6	97.6	80.8	94.3	91.2	89.9	92.6	93.5
16. PnoFKBP53	55.8	97.4	98.6	93.9	96.1	94.1	96.9	79.6	88.6	87.8	98	87.8	96	99	97.4		92.1	92.1	79.3	97.1	94.5	92.1	91.8	98
17. ProFKBP51	56.4	96.3	96.7	95.4	95.7	95.6	95.5	81	88.8	89.5	95.7	90.2	93.8	96.3	95.3	95.3		91.2	81.6	93.6	91.1	90.6	92.7	92.9
18. PruFKBP53	54.1	97.5	97.5	94.1	98.8	94.3	100	80.3	89.3	88.9	96.9	88.7	95	97.5	99	96.9	95.5		81.3	93.7	91.1	90.4	92.4	93.1
19. PstFKBP51	54.3	89.5	89.2	91	89.1	90.8	89.5	82	89.5	90.6	88.8	91	86.5	88.8	89.2	88.4	90.9	89.5		80.6	79.1	79.6	80.9	79.7
20. PcaFKBP53	54	99.2	99	94.9	97.5	95.1	98	80.1	89.5	88.7	98.4	88.5	96	99	98	98.2	96.9	98	89.3		95.8	93.5	94.3	98.8
21. PexFKBP54	55.1	97.2	98.2	93.1	95.5	93.3	96.4	79.1	88.1	87.9	98.2	87.4	97	98.2	96.6	97.8	94.9	96.4	88.1	97.8		94.5	92	96.4
22. PitFKBP52	55.3	96.7	97.3	93.2	94.9	93.4	95.5	81	87.6	88	96.3	88.4	95.8	97.3	95.3	96.3	94.8	95.5	89.4	97.1	96.6		90	93.7
23. PgrFKBP53	55.2	97.8	96.9	93.5	97.1	93.7	97.1	78.8	89.2	88.6	96.7	88.4	94.4	96.9	97.4	96.5	95.3	97.1	89.6	97.6	96.4	94.7		93.6
24. PsoFKBP53	54.1	98.2	99.2	93.9	96.7	94.1	97.6	79.3	88.8	88	98.8	87.8	96.8	99.2	97.8	98.8	95.9	97.6	88.6	99	98.8	97	97.4	

Supplementary Table S11: List of primers used for real-time PCR analysis of genes encoding different cyclophilins, FK506-binding proteins (FKBPs), parvulins and protein phosphatase 2A phosphatase activators (PTPAs) in *Penicillium oxalicum*.

S. No	Name	Sequence
Cyclophilins		
1	*PoxCYP17* (forward)	GTGTCAAGAGTGGGATGCGA
	PoxCYP17 (reverse)	ACCCTTCCTCTACCACACGA
2	*PoxCYP19* (forward)	CAAAAACCAGAAGGGCCAGC
	PoxCYP19 (reverse)	CGTCAGCAAACTTCGTGGTG
3	*PoxCYP18* (forward)	CCGCATCAACTTCAACCTCTAC
	PoxCYP18 (reverse)	CCCTTGTAGCCCTCCTTCA
4	*PoxCYP22* (forward)	CGCCTCATCCCAGGCTTTAT
	PoxCYP22 (reverse)	TGATAGATGGATGTGCCGCC
5	*PoxCYP23* (forward)	AACAAGTTCAAGGACGAGAACTTCA
	PoxCYP23 (reverse)	TGGGAACGTTCTGGATCTTGTC
6	*PoxCYP41* (forward)	GATCCAAGGTGGTGACTTTACT
	PoxCYP41 (reverse)	GTGCTTGAGGTCGAAGTTCT
7	*PoxCYP54* (forward)	GCTTTTCGTGTGCAAGCTGA
	PoxCYP54 (reverse)	GGTTCGCTTGTCTCGGATGA
8	*PoxCYP62-2* (forward)	TTTGGGAGACTGGAAGGGGA
	PoxCYP62-2 (reverse)	CGACTTCGCAAGAGGTCACT
9	*PoxCYP62-1* (forward)	GCGACGTTCAAGCGATTACC
	PoxCYP62-1 (reverse)	CTTGATCCACGGCAGGATGT
10	*PoxCYP69* (forward)	TCATGGAGATGCAGCAAGCA
	PoxCYP69 (reverse)	TTGACCTTACTTCGGGTCGC
FK506-binding proteins (FKBPs)		
11	*PoxFKBP12-2* (forward)	CTACGATGCCAAGAAGGCCA
	PoxFKBP12-2 (reverse)	TGATGACCTGTCCAACACCG
12	*PoxFKBP12-1* (forward)	GCACTACACTGGCTGTCTGT
	PoxFKBP12-1 (reverse)	ACCCTCATCCCAACCCTTGA
13	*PoxFKBP14* (forward)	TGCGTCTCACTACGCTCTTC
	PoxFKBP14 (reverse)	GTCACCGTTTTGGGTCTTGC
14	*PoxFKBP52* (forward)	CAAGGGTGTCGCTGCTAAGA
	PoxFKBP52 (reverse)	TGCCGAGCTTGAAGGTGAAA
Parvulins		
15	*PoxPAR14* (forward)	TGCGACATATCCTGTGCGAG
	PoxPAR14 (reverse)	CCTTGTCGGGCCTTATCCTC
16	*PoxPIN19* (forward)	CCGCCGATACCGATACAGAG
	PoxPIN19 (reverse)	CACGGTGCTTCACAAGGAGA
Protein phosphatase 2A phosphatase activators (PTPAs)		
17	*PoxPTPA51* (forward)	CCCGGCCTTTTTGACCTCTA
	PoxPTPA51 (reverse)	ATTGAACAGCCTCCGACTCC
18	*PoxPTPA46* (forward)	CTCCGCGCAAACAGTAATCG
	PoxPTPA46 (reverse)	GACCGGGCGTGAGTATTGAT
Reference gene		
19	*ACTIN* (forward)	GATTTGGCACCACACTTTCTAC
	ACTIN (reverse)	TCTTCTCACGGTTGGACTTG
